# Malignant Hepatoblast‐Like Cells Sustain Stemness via IGF2‐Dependent Cholesterol Accumulation in Hepatoblastoma

**DOI:** 10.1002/advs.202407671

**Published:** 2025-04-24

**Authors:** Miao Ding, Siwei Mao, Han Wu, Sijia Fang, Ni Zhen, Tianshu Chen, Jiabei Zhu, Xiaochen Tang, Xiaoyang Wang, Fenyong Sun, Guoqing Zhu, Qiuhui Pan, Ji Ma

**Affiliations:** ^1^ Department of Clinical Laboratory Shanghai Children's Medical Center Shanghai Jiao Tong University School of Medicine Shanghai 200120 P. R. China; ^2^ Department Laboratory Medicine Shanghai Tenth People's Hospital of Tongji University Shanghai 200072 P. R. China; ^3^ Faculty of Medical Laboratory Science College of Health Science and Technology Shanghai Jiao Tong University School of Medicine Shanghai 200000 P. R. China; ^4^ Shanghai Key Laboratory of Clinical Molecular Diagnostics for Pediatrics Shanghai 200120 P. R. China; ^5^ Sanya Women and Children's Hospital Managed by Shanghai Children's Medical Center Sanya 572029 P. R. China; ^6^ Shanghai Key Laboratory of Clinical Molecular Diagnostics for Pediatrics Shanghai 200127 P. R. China

**Keywords:** cholesterol, collagen 1, fibroblast, IGF2, malignant hepatoblast‐like cells, SREBF2, stemness

## Abstract

Hepatoblastoma, the most aggressive childhood liver tumor, poses significant challenges due to limited knowledge of its pathogenesis, particularly in poorly differentiated advanced tumors where the prognosis is dismal. Single‐cell sequencing provides an in‐depth exploration at the single‐cell level and offers a deep understanding of tumor heterogeneity. Herein, single‐cell transcriptomics analysis is used to identify a unique malignant‐hepatoblast (HB)‐like cell subpopulation as the possible origin of poorly differentiated hepatoblastoma. These cells are associated with an unfavorable clinical prognosis in hepatoblastoma patients. The malignant‐HB‐like cell subpopulation generated insulin‐like growth factor 2 (IGF2) to sustain stem‐like features by promoting abnormal cholesterol accumulation via SREBF2. IGF2 also stimulated fibroblast 2 to secrete collagen 1, intensifying tumor malignancy via the collagen 1/integrin α1 signaling pathway. This suggests that targeting malignant HB‐like cells by inhibiting IGF2‐induced pathways can lead to promising treatments for hepatoblastoma. Additionally, serum IGF2 levels may serve as a diagnostic biomarker for advanced hepatoblastoma. In summary, these findings provide valuable insight into the genesis and malignancy of hepatoblastoma and a foundation for more effective diagnostic tools and therapeutic strategies for this challenging disease.

## Graphical abstract

Hepatoblastoma, an aggressive childhood liver tumor, origins from malignant hepatoblast‐like cells. Single‐cell analysis reveals IGF2‐driven pathways promoting malignancy. Targeting these pathways offers promising treatments, with serum IGF2 as a potential diagnostic biomarker.

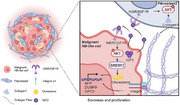

## 1 Introduction

Hepatoblastoma is the most common malignant liver tumor in children, accounting for up to 70% of pediatric liver cancers.^[^ ^1^ ^]^ Hepatoblastoma cells are morphologically similar to immature hepatocytes and are believed to originate from hepatoblasts（HB） or embryonic liver progenitor cells.^[^ ^2^ ^]^ Less differentiated HB, or cancer stem cells, may contribute to tumor aggressiveness and recurrence and have high metastatic ability. Therefore, identifying HB‐like cells and the mechanisms by which normal hepatocyte cells acquire cancer stem cell features are critical for elucidating the pathogenesis of hepatoblastoma. Furthermore, the liver tumorigenesis origin cells are associated with a more aggressive phenotype and contribute to poor patient outcomes, significantly impacting prognosis.^[^ ^3^, ^4^ ^]^ Currently, the primary treatments for hepatoblastoma include traditional adjuvant chemotherapy, hepatectomy, and liver transplantation.^[^ ^3^, ^5^, ^6^ ^]^ However, advanced and refractory cancers have high mortality rates and poor prognosis. Our limited understanding of its molecular and cellular bases impedes the development of treatment for hepatoblastoma.

The tumor microenvironment, which includes the interactions between tumor cells and surrounding liver‐resident cells, has been recognized as crucial in tumor initiation and progression.^[^ ^7^ ^]^ Single‐cell sequencing is a powerful technology that allows for a comprehensive understanding of the heterogeneous microenvironment within tumors and the identification of novel cell populations. Zhu et al.^[^ ^8^ ^]^ used single‐cell transcriptome sequencing technology to reveal that activated hepatic stellate cells in the tumor microenvironment play a decisive role in hepatoblastoma metastasis. Tang^[^ ^9^ ^]^ analyzed hepatoblastoma using single‐cell transcriptome sequencing and identified the existence of an HB1 stem cell‐like hepatocyte subpopulation, discovering that this subpopulation was strongly correlated with poor patient prognosis. However, these studies mainly relied on atlas analysis, and relatively little in‐depth exploration of the communication mechanisms between different subtypes of cells in the tumor microenvironment was conducted. Moreover, the way in which various types of cells maintain the stemness of HB tumor cells has not been elucidated. Therefore, it is crucial to deepen our understanding of hepatoblastoma tumorigenesis and develop more effective diagnosis and treatment plans.^[^ ^3^ ^]^

Insulin‐like growth factor 2 (IGF2) has a pivotal role in fetal development. Studies have revealed elevated IGF2 expression levels in various cancers. The overexpression of IGF2 is attributed to diverse mechanisms, including the reactivation of fetal IGF2 promoters, the hypermethylation of the paternal H19 locus, the loss of imprinting of the maternal IGF2 allele, and the reduced expression of tumor suppressors such as WT1 and p53.^[^ ^10^, ^11^, ^12^, ^13^ ^]^ Recent research reported that fetal liver‐like methylation patterns of IGF2 promoters were observed in the tumorous livers of children diagnosed with hepatoblastoma.^[^ ^14^ ^]^ Furthermore, patients with high levels of IGF2 hepatoblastoma are predisposed to developing more progenitor‐like tumors, which results in a marked reduction in their relapse‐free survival period.^[^ ^15^ ^]^ However, the relationship between IGF2 and tumor progenitor cells in hepatoblastoma is not clear, and the intricate mechanism of how IGF2 sustains tumor development requires further exploration.

In this study, we used single‐cell sequencing to uncover a unique hepatocyte subpopulation, malignant HB‐like cells. These cells were identified as origin cells for hepatoblastoma and were significantly associated with the poor clinical prognosis of hepatoblastoma patients. Notably, the malignant HB‐like cells generated IGF2 to maintain their stem‐like characteristics by abnormal cholesterol accumulation. Furthermore, IGF2 was shown to stimulate fibroblast 2 cells to secrete collagen 1 to intensify hepatoblastoma malignancy. We also evaluated IGF2 levels in the serum of patients with hepatoblastoma. Overall, our study findings provide a deeper understanding of poorly differentiated hepatoblastoma tumorigenesis and a foundation for the development of diagnostic and treatment approaches.

## 2 Results

### 2.1 Overall Study Design and a Single‐Cell Transcriptomic Atlas for Primary Hepatoblastoma

**Figure 1:**
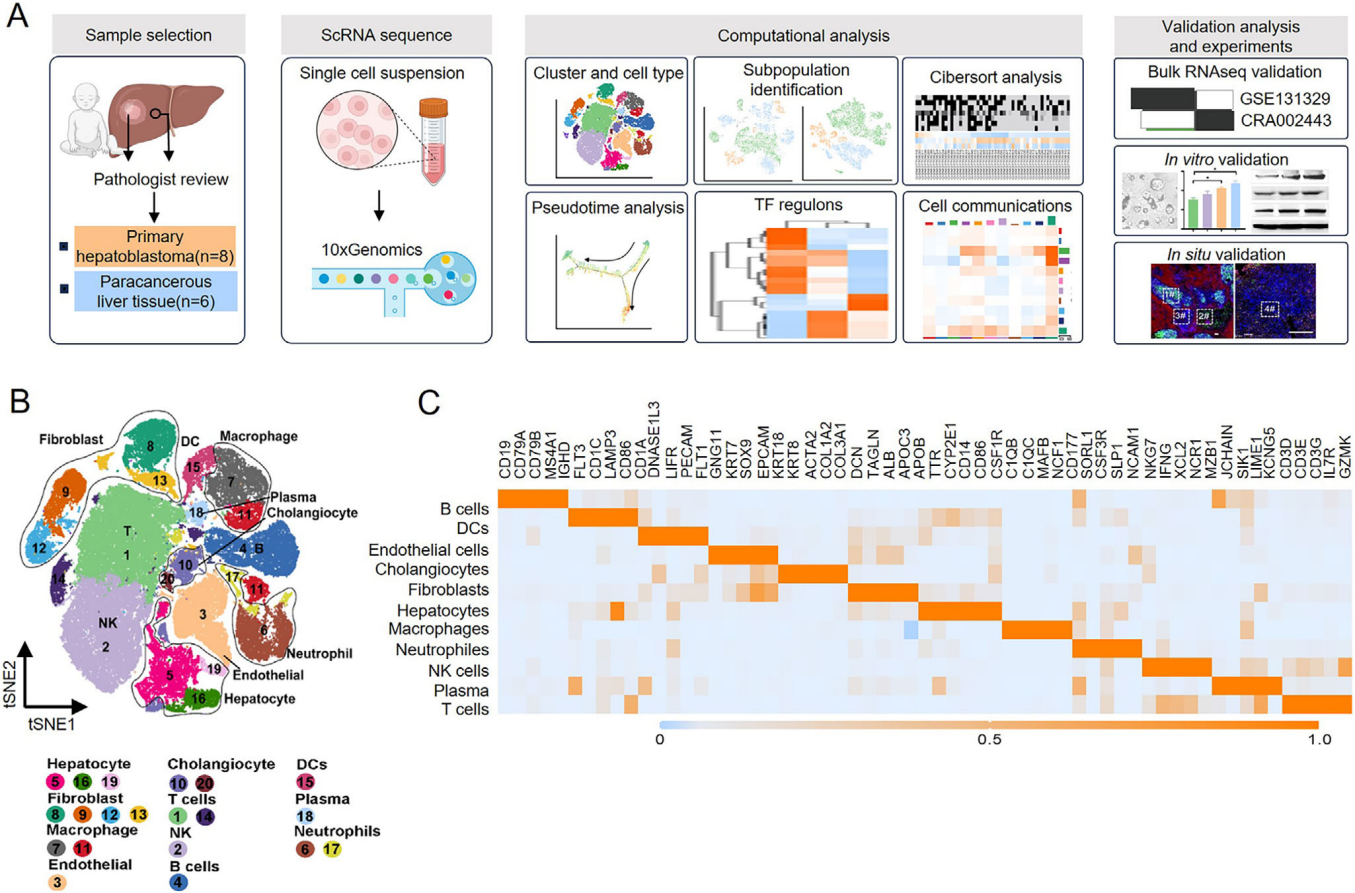
Single‐cell sequencing and cell type identification in hepatoblastoma tissue. A) Graphic overview of the study design. B) Clustering of 83 589 cells from tumorous (*n* = 8) and paracancerous (*n* = 6) pediatric liver samples, showing 20 clusters in the plot. Each cluster is shown in a different color and annotated by cell type: NK cells, DCs. C) Heatmap showing the expression levels of marker genes in the indicated cell types.

As shown in **Figure** **1**A, we analyzed hepatoblastoma and adjacent liver tissues using single‐cell RNA sequencing (scRNA‐seq) to understand hepatoblastoma's cellular diversity and molecular traits. Clustering analysis and copy number variation (CNV) analysis were employed to identify malignant HB‐like cells. Pseudotime trajectory analysis and single‐cell regulatory network inference and clustering (SCENIC) showed the enriched signaling pathways in malignant HB‐like cells. Cell communication analysis was conducted to explore intracellular support for malignant HB‐like cells from the tumor microenvironment. Deconvolution analysis was performed on public bulk RNA‐seq data to classify tumors based on their cellular composition and decipher the relationships between cellular diversity and divergent tumor biology or clinical behavior. Further validation of these potential interactions was performed using in vitro functional assays and high‐dimensional multiplex in situ analysis to provide in‐depth insight into the unique characteristics of malignant HB‐like cells.

For scRNA‐seq analysis, we obtained 14 fresh surgical resections of hepatoblastoma tumors and adjacent tissues from eight untreated patients at Shanghai Children's Medical Center, including six paired primary hepatoblastoma tissues and two additional ones. Detailed information on the patients included in this study is provided in Table S1 (Supporting Information). After quality control, 83 210 cells were analyzed, with 48 049 from tumor tissues and 35 161 from normal liver tissues. Clustering analysis identified 20 clusters representing 11 major cell types, including hepatocytes, cholangiocytes, endothelial cells, B cells, T cells, natural killer (NK) cells, macrophages, dendritic cells (DCs), fibroblasts, plasmas, and neutrophils (Figure 1B). The signature genes are detailed in Figure 1C and Table S2 (Supporting Information).

### 2.2 Malignant HB‐Like Cells Identified in Hepatocytes are Associated with an Unfavorable Clinical Prognosis in Hepatoblastoma Patients

CNV analysis was employed to distinguish malignant and non‐malignant cells. In this study, we utilized the “inferCNV” package to calculate CNVs for the 12 major cell types.^[^ ^16^ ^]^ Immune cells (CD45^+^) were employed as reference cells. Our results revealed that hepatocytes exhibited notably higher CNV levels compared to the reference cells, and liver‐stromal cells (mainly endothelial cells and fibroblasts) exhibited CNV levels similar to the reference cells (Figure S1A,B, Supporting Information).

**Figure 2:**
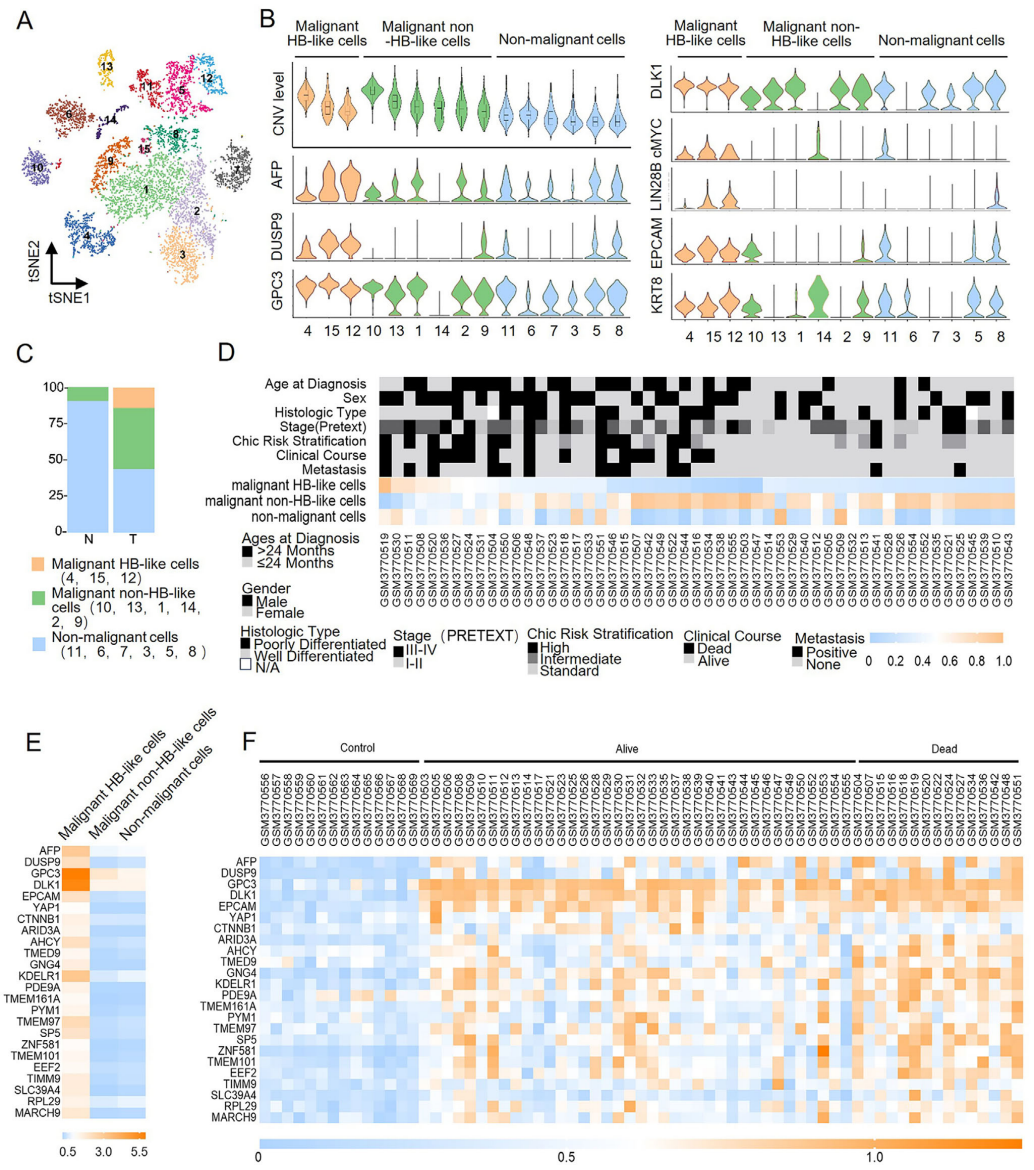
Malignant HB‐like cells identified in hepatocytes are associated with an unfavorable clinical prognosis in hepatoblastoma patients. A) T‐distributed stochastic neighbor embedding (tSNE) plot showing 15 clusters in hepatocytes from primary hepatoblastoma tumors and paired paracancerous normal liver tissues. B) Violin plots showing CNV levels and expression levels of HB‐associated genes in 15 hepatocyte clusters. C) The proportions of the three hepatocyte cell types from tumorous or paired tissue with normal origin are shown as bar plots. D) Heatmap of 67 samples from hepatoblastoma patients in GSE131329 with clinical information that underwent deconvolution analysis against the three hepatocyte subpopulations using CIBERSORTx. E) Heatmap of marker genes for the malignant HB‐like cell population in the three hepatocyte cell types. F) Heatmap of marker genes for the malignant HB‐like cell population in 67 samples from hepatoblastoma patients in GSE131329 with clinical course information.

We identified 15 clusters in hepatocytes using dimension reduction and clustering techniques (**Figure** **2**A). HBs are a cluster of embryonic liver progenitor cells, which are considered the origin of hepatoblastoma with stemness and strong positivity for alpha‐fetoprotein (AFP), dual specificity phosphatase 9 (DUSP9), Glypican 3 (GPC3), Delta like non‐canonical Notch ligand 1(DLK1), MYC proto‐oncogene (cMYC), Lin‐28 homolog B (LIN28B), Epithelial cell adhesion molecule (EPCAM), and Keratin 8 (KRT8).^[^ ^9^, ^14^, ^17^, ^18^, ^19^ ^]^ For each cluster, we plotted the CNV values and the expression levels of HB‐associated genes using violin plots (Figure 2B; Figure S2, Supporting Information).^[^ ^9^, ^14^, ^17^, ^18^, ^20^, ^21^, ^22^, ^23^, ^24^ ^]^ Then, the hepatocytes were divided into three groups: malignant HB‐like cells (clusters 4, 15, and 12, marked by high levels of CNVs and HB‐associated genes), malignant non‐HB‐like cells (clusters 10, 13, 1, 14, 2, and 9, marked by high levels of CNVs and low levels of HB‐associated genes) and non‐malignant cells (clusters 11, 6, 7, 3, 5, and 8, marked by low levels of CNVs and HB‐associated genes). Figure 2B shows that malignant HB‐like cells appeared to exhibit malignant and original stemness tendencies compared to the other two groups. Figure 2C shows a bar chart of the proportion of the three hepatocyte subpopulations in the grouped tissues.

We also investigated the association between the three hepatocyte subpopulations and the clinical characteristics of hepatoblastoma patients. We accessed a public bulk RNA‐seq dataset (GSE131329) from GEO, comprising 53 hepatoblastoma tissues and 14 noncancerous liver tissues from hepatoblastoma patients. Then, we performed a deconvolution analysis of the public data using CIBERSORTx, using the three hepatocyte subpopulations as a reference to estimate their respective proportions in each hepatoblastoma tissue sample.^[^ ^25^ ^]^ This approach enabled us to predict the composition of these hepatocyte subpopulations in hepatoblastoma tissues (Figure 2D and Table S3, Supporting Information).

We classified hepatoblastoma tissues into two groups based on the predicted proportions of malignant HB‐like cells: those with a high proportion (>25.4%, which was the mean of proportion predicted from tissues in the GSE131329 dataset) and those with a low proportion (<25.4%). Our analysis revealed significant differences between the two groups in the stage (PRETEXT, *p* = 0.025), Chic risk stratification (*p* = 0.006), clinical course (*p* = 0.007), and metastasis (*p* = 0.010) (**Table** **1**). Hepatoblastoma tissues with a higher proportion of malignant HB‐like cells indicated a more advanced stage of hepatoblastoma, implying a poorer prognosis for patients and a greater likelihood of metastasis. We observed that the expression levels of the top marker genes of malignant HB‐like cells were higher in hepatoblastoma patients with a worse prognosis (Figure 2E,F and Table S4, Supporting Information).

**Table 1:** Statistical analysis of correlations between the proportions of malignant HB‐like cells and clinical characteristics in hepatoblastoma tissues in GSE131329.

	Malignant‐HB like cells%				
	Low	High	Total	χ^2^ value	*p*‐value
Age at diagnosis					
≥24 month	14	12	26	0.332	0.564
<24 month	25	16	41		
Gender					
Male	17	17	34	1.912	0.167
Female	22	11	33		
Histologic Type					
Well‐differentiated	27	17	44	0.823	0.802
Poorly differentiated	11	10	21		
other	1	1	2		
Stage (PRETEXT)					
Normal	13	1	14	11.112	0.025
I	5	4	9		
II	9	6	14		
III	7	11	18		
IV	5	6	11		
Chic risk stratification					
High	5	9	14	12.383	0.006
Intermediate	6	2	8		
Standard	15	16	31		
NA	13	1	14		
Clinical course					
Dead	6	9	15	10.014	0.007
Alive	20	18	38		
NA	13	1	14		
Metastasis					
YES	6	7	13	9.422	0.010
NO	20	20	40		
NA	13	1	14		
Total	39	28	67		

Additionally, we searched for malignant HB‐like cells in 28 hepatoblastoma tissues with prognostic information using DUSP9 and GPC3 immunohistochemical staining to further determine the correlation between malignant HB‐like cells and the prognosis of hepatoblastoma patients (Table S1, Supporting Information). Due to the high heterogeneity of hepatoblastoma, the expression distribution of DUSP9 and GPC3 exhibited both homogeneity and heterogeneity (Figure S3A, Supporting Information). The expression intensities of DUSP9 and GPC3 vary across regions. Some tissues exhibit good homogeneity with no obvious regional differences in expression, while others display marked differences between high and low expression levels. In hepatoblastoma tissues with heterogeneous DUSP9 expression, cells with high expression of DUSP9 are considered malignant HB‐like cells. The results showed that the proportion of heterogeneous DUSP9‐positive cells in hepatoblastoma tissues with poor prognosis was significantly higher than that in hepatoblastoma tissues with good prognosis (Table S1 and Figure S3B, Supporting Information). However, the proportion of heterogeneous GPC3‐positive cells showed little difference between tissues in patients with good and poor prognoses. We speculate that differences in mRNA transcription and protein translation may make the DUSP9 protein a more representative marker of malignant HB‐like cells than the GPC3 protein. Table S5 (Supporting Information) presents the statistical analysis of correlations between the proportions of heterogeneous DUSP9/GPC3‐positive cells and the clinical course associated with the hepatoblastoma tissues. Due to the limited number of samples, a trend toward significant difference with a P value < 0.1 has been observed. These findings support the notion that malignant HB‐like cells possess a malignant tendency and play a crucial role in the tumorigenesis of hepatoblastoma.

**Table 2:** Statistical analysis of clinical characteristics correlations between the control group and hepatoblastoma group.

	Control		Hepatoblastoma			
	Counts	Percentage (%)	Counts	Percentage (%)	χ^2^ test value	*p*‐value
Age						
≤24 months	17	50.00	16	48.50	0.15	0.901
>24 months	17	50.00	17	51.50		
Gender						
Male	19	55.88	19	58.82	0.06	0.806
Female	15	44.12	14	41.18		
Total	34	100.00	33	100.00		

We also evaluated the correlation between malignant HB‐like cells and pathological subtypes by analyzing 18 samples, comprising six epithelial‐mesenchymal mixed histology type samples, six embryonic histology types, and six fetal histology types (Table S1, Figure S4A–C, Supporting Information), using DUSP9 and GPC3 immunohistochemical staining. Figure S4D (Supporting Information) displays the staining patterns of DUSP9 and GPC3 in two representative samples from the three pathological tissue types: epithelial‐mesenchymal mixed histology type, fetal histology type, and embryonic histology type. The results showed no significant differences between the different pathological subtypes in our database.

Hepatoblastoma is believed to originate from hepatoblasts or embryonic liver progenitor cells.^[^ ^2^ ^]^ The origin of hepatoblastoma was investigated by performing an integrated analysis of a public fetal and infant liver single‐cell transcriptome dataset (CRA002443, Figure S5A, Supporting Information).^[^ ^26^ ^]^ Hepatocytes were identified based on the expression levels of HNFF4A and AFP (Figure S5B,C, Supporting Information) and grouped into 26 clusters (Figure S5D, Supporting Information), with nine major cytotypes (Figure S5E, Supporting Information). Then, we integrated and clustered the hepatocytes from CRA002443 and our scRNA‐seq, as shown in Figure S5F (Supporting Information). Pearson's correlation analysis showed that malignant HB‐like cells were more similar to early fetal liver than the other two subpopuations (i.e., non‐malignant cells and malignant non‐HB‐like cells), especially the population from week 6 (Figure S5G, Supporting Information), suggesting that malignant HB‐like cells exhibit stemness tendency in hepatoblastoma.

Overall, these results revealed the malignant HB‐like cells with stemness characteristics were linked to poor prognosis and hepatoblastoma origin, indicating their crucial role in disease progression and potential as a prognostic factor.

### 2.3 Malignant HB‐Like Cells are Highly Activated in the Insulin Signaling and Cholesterol Metabolism Pathways

**Figure 3:**
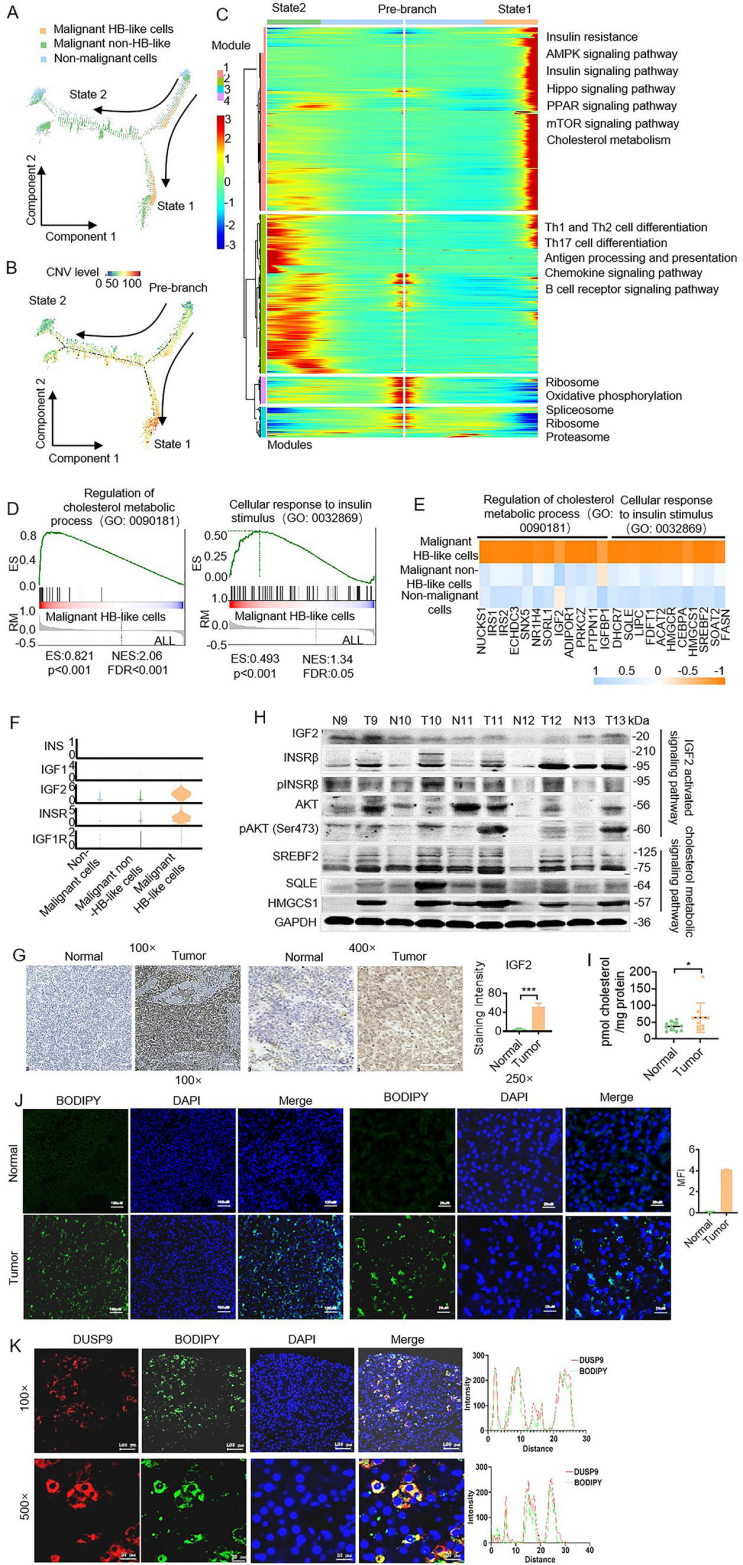
The insulin signaling pathway and cholesterol metabolism pathway in malignant hepatoblast‐like cells. A,B) Developmental trajectory of hepatocyte cells inferred by monocle2, colored by cell subtypes (A) and CNV levels (B). C) Representative signaling pathways during the developmental trajectory of hepatocyte cells inferred by monocle2. Heatmap showing the dynamic changes in gene expression along the pseudotime trajectory in branch 1 and branch 2. D) Gene set enrichment analysis (GSEA) of malignant HB‐like cells versus all hepatocyte cells in the indicated gene sets. ES, enrichment score; RM, ranked metric; NES, normalized enrichment score; FDR, false discovery rate. E. Heatmap of key gene expression related to the regulation of cholesterol metabolic process (GO: 0090181) and cellular response to insulin stimulus (GO: 0032869) in the three hepatocyte cell types. F) Violin plots showing INS, IGF1, IGF2, INSR, and IGF1R expression levels in the three hepatocyte cell populations. G) Representative IHC images of IGF2 staining of tissue sections. H) The expression levels of key proteins for the insulin signaling and cholesterol metabolic pathways in five pairs of hepatoblastoma and paracancerous tissues. I) Cholesterol concentrations were analyzed in primary hepatoblastoma tumors and paired paracancerous normal liver tissues. J) Lipid droplets (LDs) in primary hepatoblastoma tumors and paired paracancerous liver tissues were stained with BODIPY 493/503. K) BODIPY 493/503 and DUSP9 antibody co‐staining in primary hepatoblastoma tumors.

We performed pseudotime trajectory analysis. Hepatocytes were traced to two distinct trajectory branches originating from pre‐branch cells, indicating diverse developmental terminal states.^[^ ^27^ ^]^ The malignant HB‐like cells were predominantly distributed in pre‐branch and state 1 along the trajectory curve (**Figure** **3**A). The CNV levels gradually increased along the trajectory curve of state 1 (Figure 3B). We performed branched expression analysis modeling (BEAM), and gene expression changes were observed in four expression modules during the transitions (Figure S6A and Table S6, Supporting Information). Figure 3C presents the dynamic gene expression changes using a heatmap. Gene expression module 1 was mainly enriched in the state 1 stage, including insulin resistance, AMPK, the insulin signaling pathway, Hippo, PPAR, the mTOR signaling pathway, and cholesterol metabolism. State 2 stage, including Th1 and Th2 cell differentiation, Th17 cell differentiation, antigen processing and presentation, the chemokine signaling pathway, and the B cell receptor signaling pathway, were mainly observed in gene expression module 2. The pre‐branch stage, including ribosome, oxidative phosphorylation, spliceosome, and the proteasome signaling pathway, were chiefly observed in gene expression modules 3 and 4.

A gene set enrichment analysis (GSEA) was performed to confirm the enrichment of the state 1 signaling pathway in malignant HB‐like cells. The results revealed that the regulation of cholesterol metabolic processes (GO: 0090181) and cellular responses to insulin stimuli (GO: 0032869) were significantly enriched in malignant HB‐like cells (Figure 3D).^[^ ^28^ ^]^ This finding was further corroborated by the upregulation of key genes associated with cholesterol metabolism and insulin signaling in malignant HB‐like cells, suggesting a heightened activation of the insulin signaling and cholesterol metabolism pathways (Figure 3E).

In our comprehensive analysis of insulin signaling pathway ligand expression patterns, including INS, IGF1, and IGF2, in various cell types, we found that IGF2 was predominantly expressed in hepatocytes compared to the INS and IGF1 patterns (Figure S7A, Supporting Information). Specifically, IGF2 was mainly expressed in malignant HB‐like hepatocyte cells, which also exhibited high levels of INSR, the IGF2 receptor (Figure 3F). This suggests that IGF2 produced by these cells could activate the cells via autocrine mechanisms. Immunohistochemical (IHC) staining and western blot analysis revealed significantly elevated IGF2 and activated signaling pathway‐related protein levels in hepatoblastoma compared to normal liver tissues (Figure 3G,H), indicating that the IGF2 signaling pathway was activated in malignant HB‐like cells.

Hepatoblastoma exhibited significantly higher levels of cholesterol synthase (SREBF2, SQLE, HMGCS1) compared to normal liver tissues, which indicated higher metabolic activity (Figure 3H). Figure 3I shows the increased cholesterol levels in hepatoblastoma tissues compared to normal liver tissues. This was also corroborated by lipid droplet (LD) staining in patient tissues, which revealed a substantial accumulation of LDs in tumor tissues (Figure 3J; Figure S8A, Supporting Information). LDs are organelles that primarily store cholesterol esters, and their accumulation reflects abnormal cholesterol metabolism.^[^ ^29^ ^]^ DUSP9^+^ cells represent malignant HB‐like cells, and the co‐localization of LD staining in these cells further supports the presence of abnormal cholesterol metabolism in malignant HB‐like cells (Figure 3K; Figure S8B, Supporting Information).

### 2.4 IGF2 Sustains the Stemness of Hepatoblastoma via Abnormal Cholesterol Accumulation

**Figure 4:**
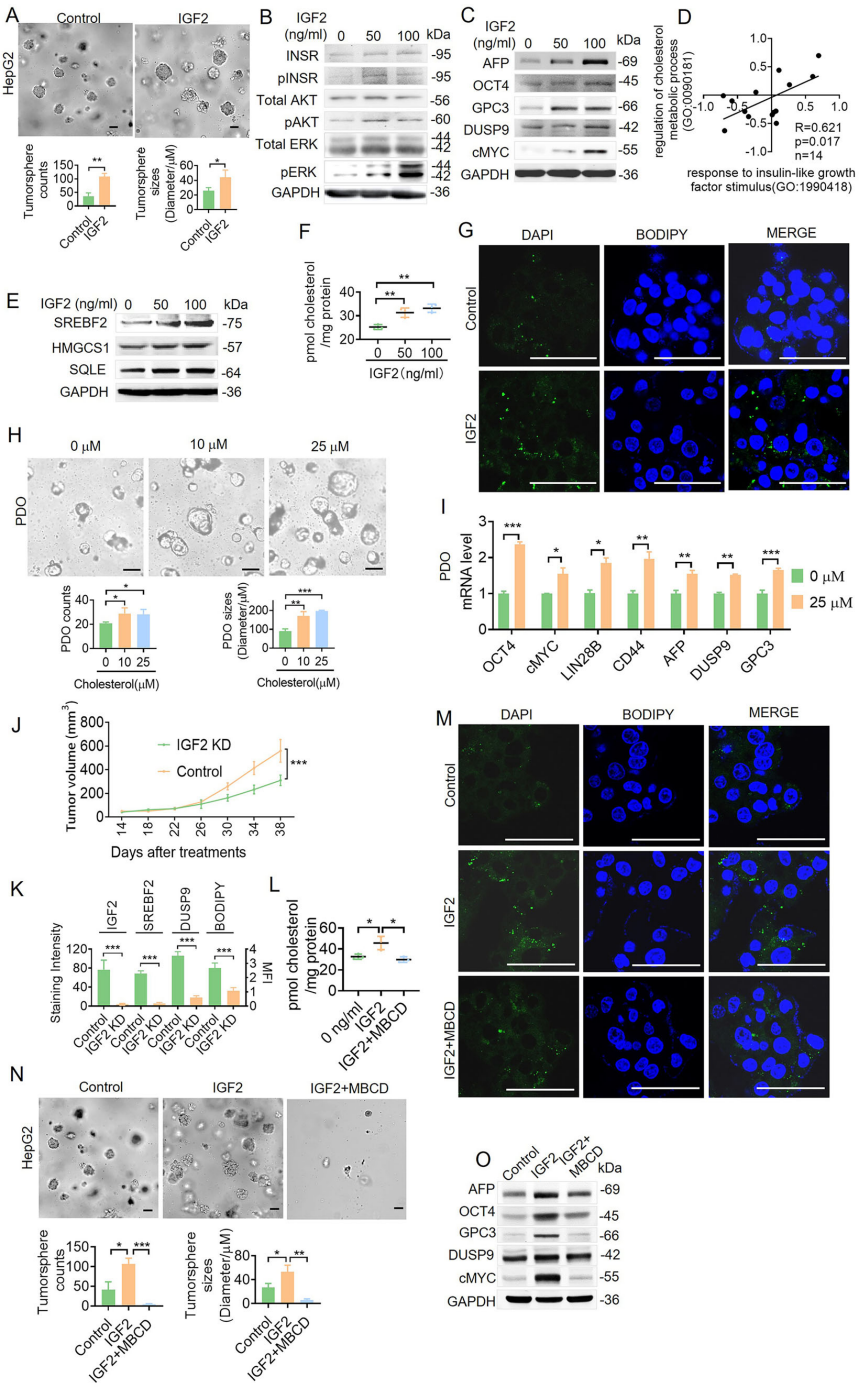
IGF2 sustains hepatoblastoma stemness via abnormal cholesterol accumulation. A) Effects of IGF2 on the tumorsphere formation of HepG2 cells. Scale bar, 50 µm. Means ± SD are shown (*n* = 3). B) The expression level of IGF2‐activated signaling pathway‐related proteins in HepG2 cells treated with IGF2 for five days was analyzed by western blots. C) The expression levels of HB stemness‐related proteins in HepG2 cells treated with IGF2 for five days were analyzed by western blots. D) Pearson's correlation analysis shows the correlation between response to insulin‐like growth factor stimulus (GO: 1990418) and the regulation of cholesterol metabolic process (GO: 0090181) in hepatoblastoma and normal tissue pairs using scRNA sequencing (*n* = 14). E) The expression levels of cholesterol metabolic pathway‐related proteins in HepG2 cells treated with IGF2 were analyzed by western blots. F) Cholesterol concentrations were analyzed in HepG2 cells treated with IGF2 for 5 days. G) Lipid droplets (LDs) in HepG2 cells treated with IGF2 for 5 days were stained with BODIPY 493/503. Scale bar, 50 µm. H) Representative images of organoids treated with cholesterol at 0, 10, and 25 µm on day 5 are shown. Scale bar, 50 µm. I) The expression of HB stemness‐related genes in hepatoblastoma PDOs with or without cholesterol treatment was analyzed by qRT‐PCR. Means ± SD (*n* = 3) are shown. J) HepG2‐control cells/HepG2‐IGF2 KD cells were subcutaneously injected into nude mice. Tumor volume changes were examined, and the mice were euthanized 40 days after tumor cell injection. K) The expression levels of IGF2, SREBF2, DUSP9, and LDs in mice tumors were detected by IHC analysis. *p < 0.05, **p < 0.01, ***p < 0.001 by the Student's *t*‐test. L) Cholesterol concentrations were measured in HepG2 cells treated with IGF2 (100 µm) and MBCD (1 mm). M) LDs in HepG2 cells treated with IGF2 (100 µm) and MBCD (1 mm) treatment were stained with BODIPY 493/503. Scale bar, 50 µm. N) Tumorsphere formation assay comparing the effect of IGF2 (100 µm) and MBCD (1 mm) treatments on HepG2 cells. Means ± SD are shown (*n* = 3). Scale bar, 50 µm. O) The expression of HB stemness‐related proteins in HepG2 cells treated with IGF2 (100 µm) and MBCD (1 mm) was analyzed by western blots.

We first performed a transcriptomic evaluation of the two well‐recognized hepatoblastoma cell lines, HepG2 and HUH6, using Pearson's analysis with gene signatures in the public GSE168997 dataset to elucidate IGF2″s role in hepatoblastoma (AFP, DUSP9, GPC3, DLK1, MYC, EPCAM, KRT8, and LIN28B; Figure S9A, Supporting Information). HUH6 and HepG2 cells exhibited stronger transcriptomic activation of AFP, DUSP9, GPC3, DLK1, MYC compared to other non‐hepatoblastoma cell lines. Next, we performed GSVA analysis with the three identified hepatocyte subpopulations (Figure S9B, Supporting Information). HepG2 cells exhibited stronger transcriptomic characteristics of malignant HB‐like cells than HUH6 cells. Then, we investigated the impact of IGF2 on tumorigenesis. The results revealed that IGF2 significantly enhanced cell proliferation and tumorsphere formation (**Figure** **4**A; Figure S10A–C, Supporting Information). The Western blot analysis of IGF2‐treated cells demonstrated increased activation of the IGF2‐related signaling pathway (Figure 4B; Figure S10D, Supporting Information) and the increased expression of HB‐associated proteins, including AFP, OCT4, GPC3, DUSP9, and cMYC, indicating IGF2's role in sustaining hepatoblastoma stemness (Figure 4C; Figure S10E, Supporting Information).

Given that both the IGF2‐related signaling pathway and cholesterol metabolism pathway in malignant HB‐like cells were activated, we examined their correlation. The Pearson correlation analysis revealed a significant positive association between the response to IGF2 stimulus (GO: 1990418) and regulation of the cholesterol metabolic process (GO: 0090181) in the hepatoblastoma and normal tissue pairs (Figure 4D and Table S7, Supporting Information), suggesting that IGF2 may induce the activation of cholesterol metabolism. Western blot analysis showed the upregulation of key cholesterol synthetic enzymes, including SREBF2, HMGCS1, and SQLE, in IGF2‐treated HepG2 cells (Figure 4E; Figure S10F, Supporting Information). Figure 4F and Figure S10G (Supporting Information) show elevated cholesterol levels in IGF2‐treated cells. We also performed LD staining, which revealed a significant increase in HepG2 cells treated with IGF2 (Figure 4G; Figure S10H, Supporting Information). These results suggest that IGF2 regulates cholesterol metabolism and elevates cholesterol levels in hepatoblastoma cells.

We investigated the impact of cholesterol on tumorigenesis to further elucidate IGF2's role in hepatoblastoma. Hepatoblastoma cell lines treated with cholesterol exhibited enhanced growth, as evidenced by tumorsphere assays and the CCK8 assay (Figure S11A–C, Supporting Information). Western blot analysis of cholesterol‐treated cells showed the increased expression of HB‐associated proteins (AFP, OCT4, GPC3, DUSP9, and cMYC; Figure S11D, Supporting Information). Similarly, patient‐derived organoids (PDOs) treated with cholesterol exhibited enhanced growth, as evidenced by the Cell‐Titer‐Glo assay (Figure 4H). qRT‐PCR analysis revealed that cholesterol significantly upregulated HB‐associated gene expression (Figure 4I). These findings indicate that cholesterol significantly enhances HB stemness characteristics.

We constructed an IGF2 knocked down (IGF2 KD) HepG2 cell line to further determine the function of IGF2. Cell proliferation was slower in IGF2 KD cells compared to HepG2 control cells (Figure S12A, Supporting Information). Western blot analysis showed the decreased expression of HB‐associated genes like AFP, OCT4, GPC3, and DUSP9 in IGF2 KD HepG2 cells, indicating that decreasing IGF2 could reduce hepatoblastoma stemness (Figure S12B, Supporting Information). We established a HepG2 xenograft model in nude mice to further determine the function of IGF2 in vivo. We observed that the downregulation of IGF2 could reduce tumor growth (Figure 4J; Figure S12C–E, Supporting Information). We also found that SREBF2, DUSP9, and BODIPY decreased with IGF2 knocked down (Figure 4K; Figure S12F, Supporting Information). In all, our results indicated that IGF2 downregulation significantly reduced cholesterol accumulation and stemness maintenance in vitro and in vivo.

IGF2 elevated cholesterol levels in hepatoblastoma cells, which significantly accelerated HB stemness characteristics. However, when cholesterol was depleted from IGF2‐treated hepatoblastoma cells using methyl‐b‐cyclodextrin (MBCD, which is extensively used as a cholesterol‐depleting reagent based on its property of solubilizing non‐polar substances Figure 4L,M; Figure S13A,B, Supporting Information), IGF2's effects were significantly diminished (Figure 4N,O; Figure S13C–F, Supporting Information). These findings underscore the crucial role of cholesterol in IGF2‐induced tumorigenesis and stemness in sustaining hepatoblastoma.

### 2.5 SREBF2 Mediates Abnormal Cholesterol Accumulation in the Activated IGF2 Signaling Pathway

**Figure 5:**
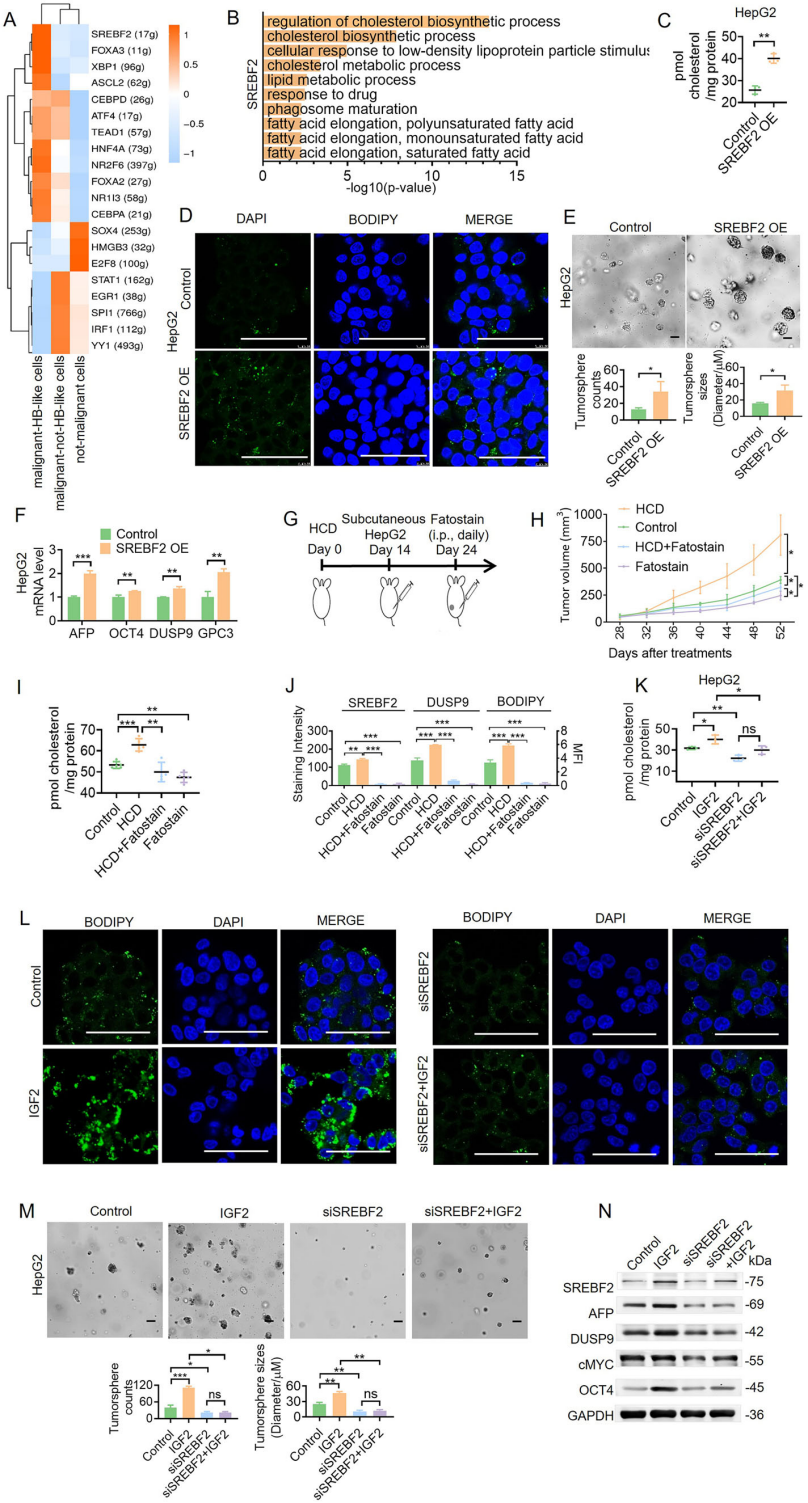
SREBF2 mediates abnormal cholesterol accumulation in the activated IGF2 signaling pathway. A) Regulon activity scores of three hepatocyte cell types were calculated using SCENIC. B) GO enrichment analysis for SREBF2 target genes calculated using SCENIC. C) Cholesterol concentrations were analyzed in HepG2 cells with SREBF2 expression. D) LDs were stained with BODIPY 493/503 in HepG2 cells with SREBF2 overexpression. Scale bar, 50 µm. E) Tumorsphere formation assay comparing the effect of SREBF2 on HepG2 cells. Means ± SD are shown (*n* = 3). F) The expression of HB stemness‐related proteins was analyzed in HepG2 cells with SREBF2 overexpression using RT‐PCR. G) Schematic diagram of HCD and Fatostain combined treatment. Mice were injected with HepG2 tumor cells subcutaneously after 14 days of HCD feeding. Ten days later, the tumor‐bearing mice were treated with Fatostain at 15 mg kg^−1^ daily. H) Tumor volume changes were examined, and the mice were euthanized 52 days after treatment. I) Mice were euthanized, and the cholesterol concentrations were measured in tumor tissues. Data represent the mean ± SD of five samples in each group per time point. J) The expression levels of SREBF2, DUSP9, and LDs in mice tumors were analyzed using IHC staining. *p < 0.05, **p < 0.01, ***p < 0.001 by the Student's *t*‐test. K) Cholesterol concentrations were analyzed in HepG2 cells treated with IGF2 and SREBF2 siRNA. L) LDs in HepG2 cells with the indicated treatments were stained with BODIPY 493/503. Scale bar, 50 µm. M) Tumorsphere formation assay comparing the effect of the indicated treatments on HepG2 cells. Means ± SD are shown (*n* = 3). N) The expression of HB stemness‐related proteins in HepG2 cells with the indicated treatments was analyzed by western blots.

We utilized the single‐cell regulatory network inference and clustering (SCENIC) method to uncover the pivotal transcription factors regulating malignant HB‐like cells.^[^ ^30^, ^31^ ^]^ The findings identified SREBF2 as the top‐activated transcription factor in these cells (**Figure** **5**A). SREBF2 is a ubiquitously expressed transcription factor that controls cholesterol homeostasis by regulating the transcription of sterol‐regulated genes.^[^ ^32^ ^]^ Further, GO enrichment analysis also revealed the significant enrichment of cholesterol metabolic genes regulated by SREBF2 (Figure 5B and Table S8, Supporting Information).

We overexpressed SREBF2 in HepG2 cells to elucidate the role of SREBF2 in IGF2‐driven hepatoblastoma tumorigenesis (Figure S14A, Supporting Information). RT‐PCR analysis revealed a significant upregulation of cholesterol metabolism genes, including FASN, LCAT, NPC1L1, SQLE, and HMGCS1 (Figure S14B, Supporting Information). This upregulation correlated with increased cholesterol levels and enhanced LD staining (Figure 5C,D; Figure S14C, Supporting Information). SREBF2 significantly accelerated cell proliferation and tumorsphere formation, as shown in the CCK8, colony formation, and tumorsphere formation assays (Figure 5E; Figure S14D,E, Supporting Information). RT‐PCR analysis showed the increased expression of HB‐associated genes like AFP, GPC3, and DUSP9, indicating that SREBF2 could enhance hepatoblastoma stemness (Figure 5F). However, silencing SREBF2 in HepG2 and HUH6 cells significantly reduced cholesterol accumulation and stemness maintenance (Figure S15A–H, Supporting Information).

Fatosatin is an SREBF2 activation inhibitor. It significantly inhibited cell proliferation in HepG2 and HUH6 cells (Figure S16A,B, Supporting Information). Western blot analysis showed that Fatostain treatment decreased the expression of HB‐associated genes like AFP, OCT4, GPC3, and DUSP9, indicating that SREBF2 inhibition could reduce hepatoblastoma stemness (Figure S16C, Supporting Information). We investigated the tumorigenesis‐promoting function of SREBF2 and cholesterol in vivo by establishing a HepG2 xenograft model in nude mice using a high‐cholesterol diet (HCD) and Fatostain treatment, as outlined in Figure 5G. We observed that the HCD could contribute to tumorigenesis while Fatostain significantly inhibited tumor growth (Figure 5H). The mice were bled when they were euthanized, and the concentrations of cholesterol in tumor tissue and serum were measured (Figure 5I; Figure S16D, Supporting Information). The HCD could partially prevent inhibition by Fatostain treatment (Figure S16E–G, Supporting Information). We also found that Fatostain treatment decreased DUSP9 and BODIPY (Figure 5J; Figure S16H, Supporting Information). In all, our results indicated that SREBF2 inhibition significantly reduced cholesterol accumulation and stemness maintenance in vitro and in vivo.

Our experiments demonstrated that IGF2 treatment elevated SREBF2 levels in HepG2 cells (Figure 4E). The role of SREBF2 in IGF2‐induced hepatoblastoma was further investigated by silencing SREBF2 in IGF2‐treated HepG2 cells. This silencing significantly abrogated the effects of IGF2 on cholesterol accumulation (Figure 5K,L; Figure S17A, Supporting Information) and stemness maintenance (Figure 5M,N; Figure S17B,C, Supporting Information). Our findings indicate that SREBF2 facilitates abnormal cholesterol accumulation and stemness maintenance in the activated IGF2 signaling pathway.

We employed an AKT pathway inhibitor, LY294002, which selectively inhibits PI3‐kinase and blocks the PI3‐kinase/Akt signaling pathway to further confirm that the regulation of SREBF2 by IGF2 is mediated through the pAKT pathway.^[^ ^11^, ^12^ ^]^ When cells were treated with IGF2 and LY294002 (Figure S18A, Supporting Information), the pAKT pathway was blocked, and consequently, SREBF2 induction was prevented (Figure S18B, Supporting Information). The corresponding cellular phenotypes were also observed (Figure S18C–G, Supporting Information). These findings provide more evidence supporting that IGF2 increases SREBF2 through the pAKT pathway.

Additionally, we simultaneously detected the expression levels of IGF2, SREBF2, DUSP9, and LDs in consecutive sections of 16 hepatoblastoma patient tissues. The detailed clinical information is provided in Table S1 (Supporting Information). Among them, IGF2 and SREBF2 represent the activated IGF2 signaling pathway, DUSP9 represents malignant HB‐like cells, and the LD dye BODIPY 493/503 represents abnormally accumulated cholesterol. Figure S19A,B (Supporting Information) present representative tissue section images and positive area analysis. We classified the areas into strong‐positive (Strong‐S), moderate‐positive (Moderate‐M), and weak‐positive (Weak‐W) based on the intensity of the positive signals. The results demonstrated the co‐localization of IGF2, SREBF2, DUSP9, and LDs. Figure S19C and Table S9 (Supporting Information) also show a significant correlation of the expression levels of IGF2, SREBF2, DUSP9, and LDs across the 16 hepatoblastoma tissues. The above results prove that abnormally accumulated cholesterol is a relatively significant characteristic in malignant HB‐like cells with an activated IGF2 signaling pathway.

### 2.6 Distinct Stromal Cell Subpopulations in Hepatoblastoma Tissues

Fibroblasts, liver endothelial cells, and macrophages are the three main stromal cells in hepatoblastoma tissues. The three distinct fibroblast subpopulations were identified in hepatoblastoma. Fibroblasts, as mesenchymal cells, crucially contribute to liver differentiation, homeostasis, and proliferation by extracellular matrix (ECM) production.^[^ ^33^, ^34^, ^35^ ^]^ Our study clustered 10934 fibroblast cells into three subpopulations, each exhibiting unique transcriptomic signatures (Figure S20A, Supporting Information). Fibroblast 2 cells notably expressed ECM markers, including COL1A1, COL1A2, LUM, FN1, and ACTA2 (Figure S20B, Supporting Information). The proportions of these subtypes in tumorous and adjacent tissues are depicted in Figure S20C,D (Supporting Information), indicating heterogeneous fibroblasts in hepatoblastoma. GO analysis for this subtype revealed their association with the ECM, collagen binding, and cytokine‐mediated signaling pathway (Figure S20E and Table S10, Supporting Information).^[^ ^36^ ^]^

Fibroblast 1 cells comprised 57% of the fibroblast population, resembling fibroblast 2 cells with high ECM markers such as COL1A1, COL1A2, and LUM. Fibroblast 3 cells, primarily derived from adjacent tissues, exhibited myovascular signatures, including ACTA2, RGS5, and GJA4. GO analysis revealed enrichment in type I interferon signaling, hypoxia response, and muscle contraction, consistent with their microvascular signatures.

Monocle2 was used to map the differentiation pseudotime trajectory of the three fibroblast populations.^[^ ^27^ ^]^ Fibroblast 3 cells were defined as pre‐branch cells due to their higher proportions in normal liver tissues. Figure S20F (Supporting Information) depicts their developmental trajectory, color‐coded by the identified three subtypes. BEAM analysis revealed four dynamic gene expression change modules during the transitions (Figure S20G and Table S11, Supporting Information), highlighting focal adhesion, ECM‐receptor interaction, and PI3K/Akt signaling in fibroblast 2 cells, as well as ribosomes, the cell cycle, and proteasome functions during the transition from fibroblast 1 cells.

Three different liver endothelial cells (LSECs) were identified in hepatoblastoma (Figure S21A, Supporting Information). These cells, comprising central venous LSECs, periportal LSECs, and portal endothelial cells, form a dynamic barrier between the blood and the liver microenvironment. Based on established markers, we classified them as central venous LSECs (LYVE1, CD32, CD14, STAB2, and CD299), periportal LSECs (LYVE1, CD32, CD14, STAB2, CD299, VWF, and CD31), and portal endothelial cells (VWF and CD31; Figure S21B, Supporting Information).^[^ ^37^, ^38^ ^]^ Figure S21C,D (Supporting Information) illustrates the three endothelial cell populations in the tumor and paired normal tissues. GO analysis revealed enrichment in ECM and collagen‐related terms in these endothelial cells (Figure S21E and Table S12, Supporting Information).

Tumor‐associated macrophages exhibit polarization states favoring pro‐tumoral functions. Macrophages were identified by high CD163 and CD68 expression (Figure S22A, Supporting Information).^[^ ^39^ ^]^ These macrophages were further distinguished as M1 or M2 subtypes using FCGR3A, CCR2, IL1A, IL1B (M1), and CD163 markers (M2; Figure S22B, Supporting Information).^[^ ^40^, ^41^ ^]^ The high proportion of M2 macrophages in tumorous tissues suggests a pro‐tumoral polarization state (Figure S22C,D, Supporting Information). We further compared the changes during tumorous transformation by differential gene analysis and KEGG enrichment, which revealed significant enrichment in cholesterol metabolism in tumor‐derived macrophages (Figure S22E and Table S13, Supporting Information).

### 2.7 Contribution of Fibroblast 2 Cells to Hepatoblastoma‐Like Cell Malignancy and Stemness via the Collagen 1/Integrin α1 Axis

**Figure 6:**
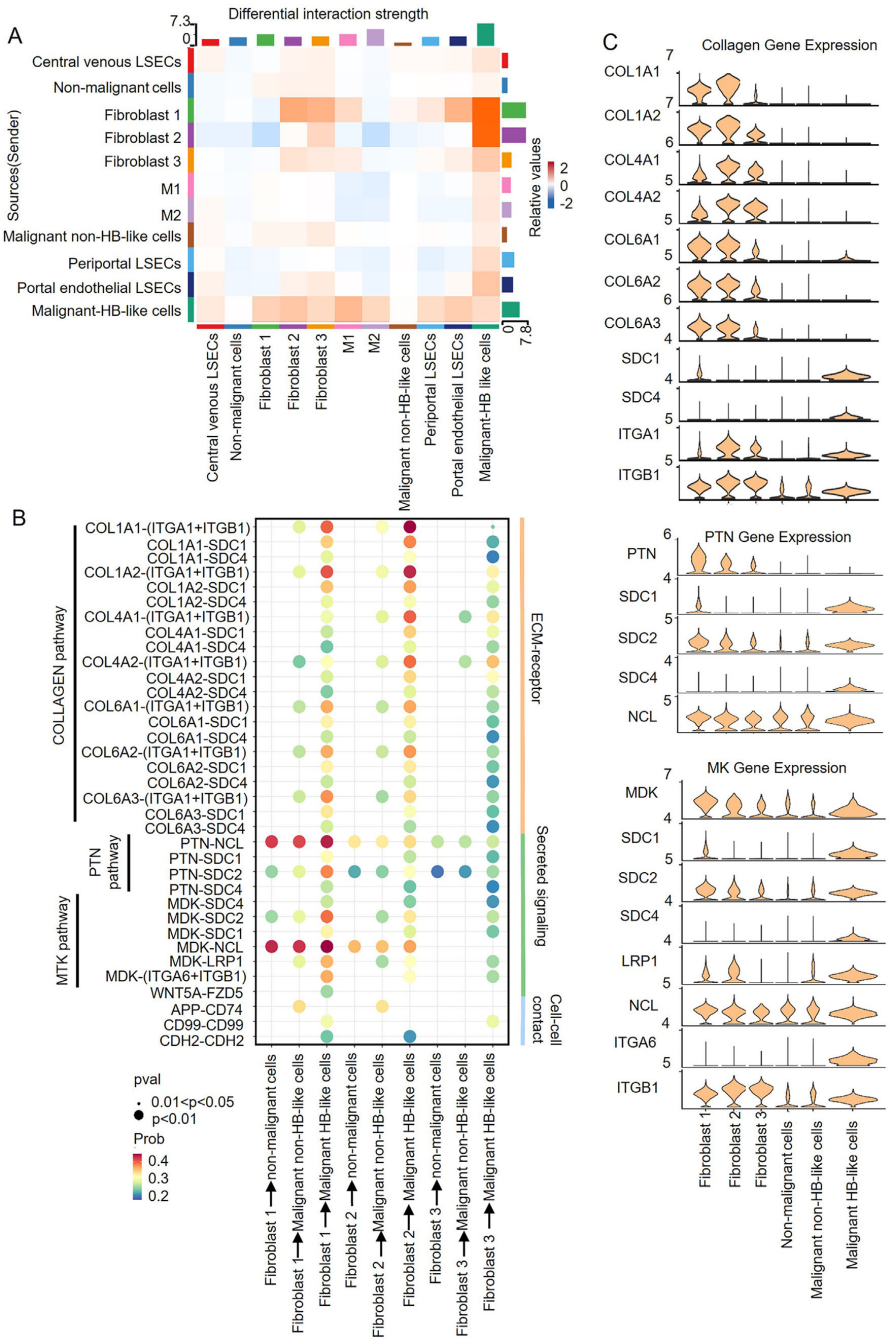
Intercellular crosstalk in hepatoblastoma. A) Heatmap showing the number and strength of interactions between cell subtypes in hepatoblastoma. The color represents the communication probability in hepatoblastoma tumors compared to normal paracancerous liver tissues. B) Significant ligand‐receptor pairs that contribute to signaling sending between hepatocytes and fibroblasts. The dot color and size represent the calculated communication probability and the *p*‐value. C) Significant ligand‐receptor expression levels that contribute to signaling sending between hepatocytes and fibroblasts.

The tumor microenvironment significantly impacts tumorigenesis and progression.^[^ ^7^, ^42^ ^]^ Our intercellular interaction analysis utilizing ligand‐receptor pairs aimed to elucidate the dynamic regulations between malignant HB‐like cells and stromal cell subpopulations.^[^ ^43^ ^]^ We identified 392 ligand‐receptor pairs mediating 121 cell cluster interactions. The number of interactions was comparable between tumor tissue and adjacent normal liver tissue (Figure S23A, Supporting Information). Figure S23B (Supporting Information) shows that the strength of these interactions was marginally stronger in tumor tissue than in adjacent normal tissue. The heatmaps illustrated robust interactions between malignant HB‐like cells and fibroblast 1 and 2 cells (**Figure** **6**A; Figure S23C, Supporting Information). Further analysis revealed concentrated collagen‐associated pathways in the communication between fibroblast 1 and 2 cells and malignant HB‐like cells, particularly robust collagen 1/integrin α1 interactions (Figure 6B). This was also validated by violin plots, which showed elevated collagen 1 and integrin α1 (ITGA1) expression in fibroblast 1 and 2 cells (Figure 6C).

Collagen 1, a key extracellular matrix component, is often overexpressed during tumorigenesis (Figures S20G, Supporting Information).^[^ ^44^ ^]^ We suggest that fibroblast 2 cells secrete collagen 1 via IGF2‐induced PI3K/AKT pathway activation, supported by PI3K/AKT enrichment and IGF2 receptor (IGF1R) expression (Figure S20H,I, Supporting Information).^[^ ^45^, ^46^, ^47^, ^48^ ^]^ Integrin signaling plays a pivotal role in orchestrating stem cell functions, including tumor initiation, epithelial plasticity, and metastatic reactivation.^[^ ^49^, ^50^ ^]^ Thus, we cultured HepG2 cells with exogenous collagen 1 and observed accelerated proliferation as shown by the CCK8 and colony formation assays (Figure S24A–C, Supporting Information) and upregulated HB‐related genes like AFP, Lin28B, cMYC, OCT4, DUSP9, and GPC3 (Figure S24D, Supporting Information). However, silencing integrin a1 (ITGA1) in HepG2 cells significantly abrogated these effects. These findings suggest that blocking the collagen 1/integrin a1 axis disrupts fibroblast‐malignant cell interactions, offering a potential therapeutic strategy for hepatoblastoma.

**Figure 7:**
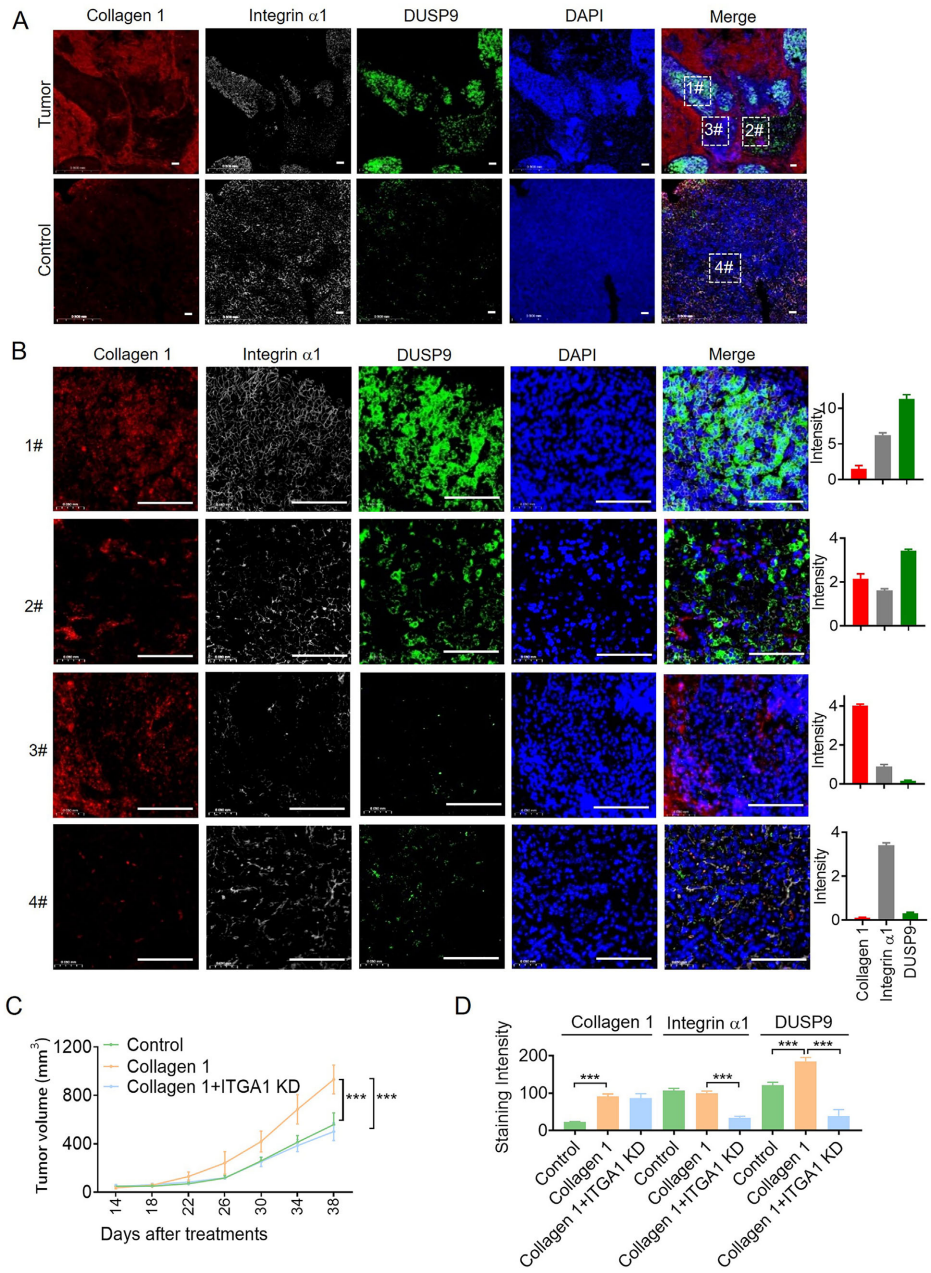
Contribution of fibroblast 2 cells to hepatoblast‐like cell malignancy and stemness via the collagen 1/integrin α1 axis. A) Representative immunofluorescence images of human hepatoblastoma and noncancerous liver tissue. DUSP9 (green), integrin α1 (gray), collagen 1 (red), and DAPI (blue) in merged, and individual channels are shown. B) Representative immunofluorescence images of human hepatoblastoma tissue. DUSP9 (green), integrin α1 (gray), collagen 1 (red), and DAPI (blue) in merged, and individual channels are shown. For fluorescence quantification, three to five representative images were quantified for each sample using ImageJ. Scale bar, 100 µm. *p < 0.05, **p < 0.01, ***p < 0.001. C. HepG2‐control cells or HepG2‐ITGA1 KD cells were subcutaneously injected into nude mice with and without collagen 1 treatment. Tumor volume changes were examined, and the mice were euthanized 40 days after tumor cell injection. D. The expression levels of integrin α1, collagen 1, and DUSP9 in mice tumors were detected by ICH analysis. *p < 0.05, **p < 0.01, *** *P* < 0.001 by the Student's *t* test.

Integrin α1, a subunit of integrin, and DUSP9^+^ cells, representing malignant HB‐like cells, were investigated using multiplexed immunofluorescent staining (MIF) of hepatoblastoma tissues. The upper panel in **Figure** **7**A shows hepatoblastoma tissue rich in collagen 1 and integrin α1 with more DUSP9^+^ cells, and the lower panel depicts hepatoblastoma tissue low in collagen 1 and high in integrin α1, resulting in fewer DUSP9^+^ cells. More detailed information is shown in Figure 7B, where the proportion of DUSP9^+^ cells was positively correlated with integrin α1 expression in a collagen 1‐rich environment. This indicates that collagen 1 and integrin α1 jointly contribute to DUSP9^+^ cell development, supporting their intercellular interactions.

We constructed HepG2 cells with integrin α1 knock‐down (ITGA1 KD) and established a HepG2 xenograft model in nude mice to further determine the tumorigenesis‐promoting function of collagen 1 and integrin α1 in vivo (Figure 7C; Figure S25A, Supporting Information). We observed that collagen 1 treatment contributed to HepG2 tumorigenesis, while the treatment of HepG2‐ITGA1 KD cells with collagen 1 did not (Figure S25B–D, Supporting Information). We also found that DUSP9^+^ cells were positively correlated with integrin a1 expression in a collagen 1‐rich environment (Figure 7D; Figure S25E, Supporting Information).

### 2.8 Serum IGF2 is a Potential Diagnostic Biomarker for Hepatoblastoma Patients

**Figure 8:**
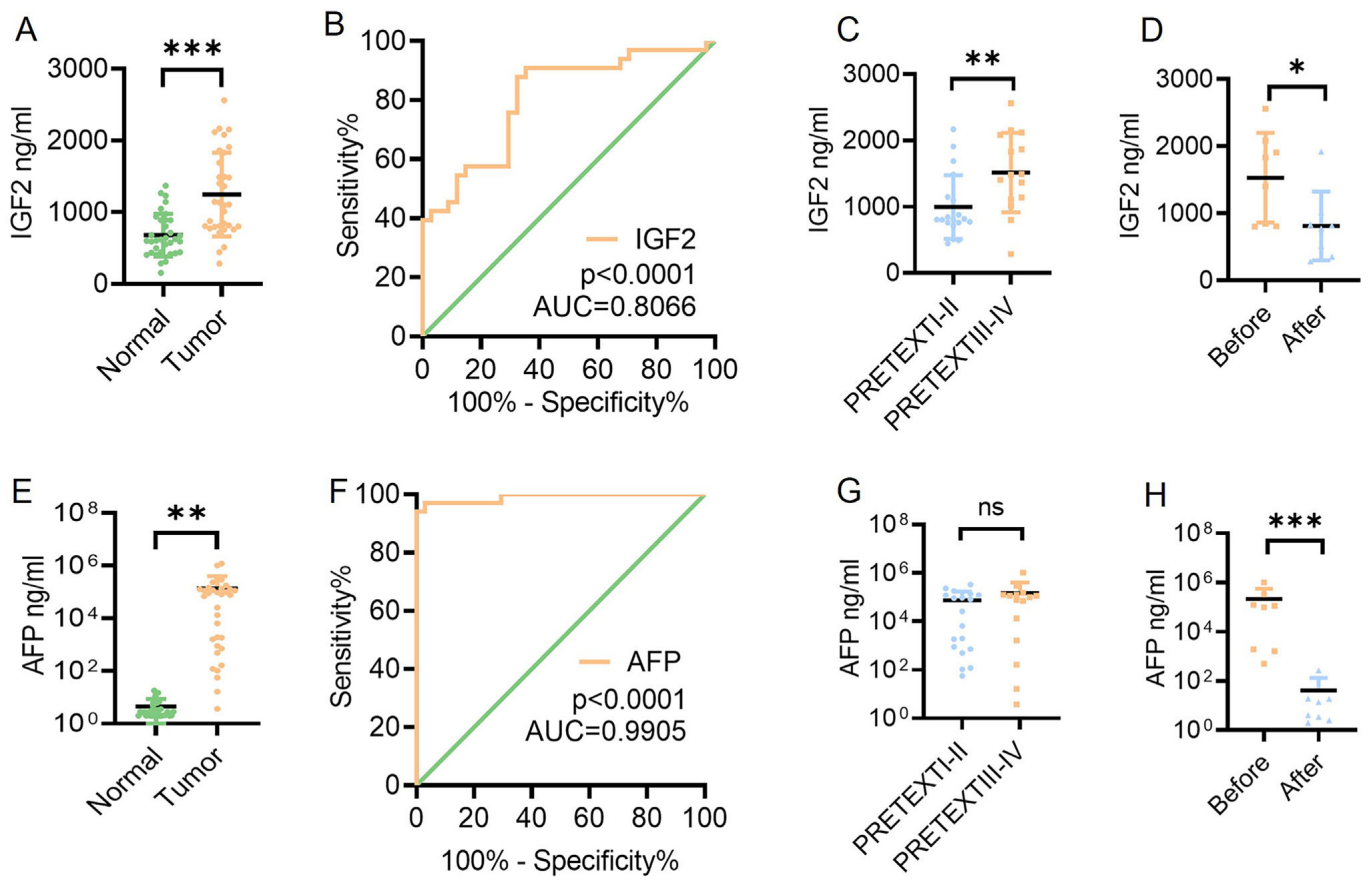
Serum IGF2 is a potential diagnostic biomarker for hepatoblastoma patients. A) The column scatter plot shows IGF2 concentrations in serum in the hepatoblastoma group and the control group of children. B) The ROC curve for using serum IGF2 as a diagnostic biomarker. C) The column scatter plot shows serum IGF2 concentration in the PRETEXTI‐II hepatoblastoma group and the PRETEXT III‐IV hepatoblastoma group. D) The column scatter plot shows serum IGF2 concentrations in the before‐treatment hepatoblastoma group and the after‐treatment hepatoblastoma group. E) The column scatter plot shows serum AFP concentrations in the hepatoblastoma group and the control group of children. F) The ROC curve for using serum AFP levels as a diagnostic biomarker. G) The column scatter plot showed serum AFP concentrations in the PRETEXTI‐II hepatoblastoma group and the PRETEXT III‐IV hepatoblastoma group. H) The column scatter plot shows serum AFP concentrations in the before‐treatment hepatoblastoma group and the after‐treatment hepatoblastoma group. *p* < 0.05, **p < 0.01, ***p < 0.001.

Serum IGF2 expression levels in hepatoblastoma patients were measured using an ELISA assay. The study comprised 34 healthy individuals and 33 hepatoblastoma patients, whose general characteristics are outlined in Table 2 and Figure S14 (Supporting Information). Notably, the results revealed a significant upregulation of serum IGF2 levels in the hepatoblastoma group (**Figure** **8**A). A ROC curve analysis of IGF2 was also conducted to evaluate its potential as a diagnostic marker for distinguishing hepatoblastoma patients from healthy individuals. As shown in Figure 8B, IGF2 exhibited a significant diagnostic value (AUC = 0.8066; *p* < 0.0001). IGF2 levels above 735.7 ng mL^−1^ indicated a high probability of hepatoblastoma, with 87.88% sensitivity and 69.70% specificity.

Next, we classified hepatoblastoma patients into two groups based on IGF2 levels: high (≥1000 ng mL^−1^) and low (<1000 ng mL^−1^) groups. Our analysis revealed a significant difference in the stage between these two groups (PRETEXT, *p* = 0.036), whereas no differences were noted for other clinicopathological factors (**Table** **3**). Patients with PRETEXT III‐IV hepatoblastoma exhibited significantly elevated serum IGF2 levels compared to those with PRETEXT I‐II hepatoblastoma (*p* = 0.0086; Figure 8C). This finding suggests that IGF2 levels may be linked to the progression of hepatoblastoma. Additionally, post‐treatment hepatoblastoma patients displayed reduced IGF2 levels compared to their pre‐treatment counterparts, indicating that serum IGF2 levels could potentially serve as a biomarker for treatment response (Figure 8D).

**Table 3:** Statistical analysis of correlations between clinical characteristics and IGF2 levels in serums from hepatoblastoma patients.

Hepatoblastoma (*n* = 33)	IGF2		χ^2^ test value	*p*‐value
	Low(*n* = 14)	High(*n* = 19)		
Age				
≤24 months	6 (42.9%)	12 (63.2%)	1.340	0.247
>24 months	8 (57.1%)	7 (36.8%)		
Gender				
Female	4 (28.6%)	10 (52.6%)	1.910	0.167
Male	10 (71.4%)	9 (47.4%)		
PRETEXT				
I‐II	11 (78.6%)	8 (42.1%)	4.388	0.036
III‐IV	3 (21.4%)	11 (57.9%)		
Metastasis				
Yes	6 (42.9%)	4 (21.1%)		0.257
No	8 (57.1%)	15 (78.9%)		
AFP				
≤2000 ng mL^−1^	6 (42.9%)	5 (26.3%)	0.992	0.319
>2000 ng mL^−1^	8 (57.1%)	14 (73.7%)		
Histology				
Epithelial	4 (28.6%)	6 (31.6%)	0.849	0.792
MIX	7 (50%)	11 (57.9%)		
NA	3 (21.4%)	2 (10.5%)		
Total	14 (100%)	19 (100%)		

Concurrently, we conducted a comparative analysis of serum AFP levels in the hepatoblastoma group. Serum AFP levels are considered the most significant diagnostic marker for hepatoblastoma, as elevations are seen in 90% of tumor patients.^[^ ^51^ ^]^ Consistent with our predictions, serum AFP levels were significantly elevated in hepatoblastoma patients compared to healthy individuals (Figure 8E). The subsequent ROC curve analysis further validated the diagnostic value of AFP in distinguishing hepatoblastoma patients from healthy individuals, with an AUC of 0.9905 (*p* < 0.0001, Figure 8F). We classified hepatoblastoma patients into two groups based on the AFP levels.^[^ ^44^ ^]^ Table S15 (Supporting Information) revealed no significant differences in clinicopathological factors between these two groups. Additionally, no significant differences in serum AFP levels were seen between PRETEXT III‐IV and PRETEXT I‐II hepatoblastoma patients (Figure 8G). These findings underscore the pivotal role of IGF2 in hepatoblastoma self‐sustaining regulation. Figure 8H reveals a notable decrease in AFP levels in post‐treatment hepatoblastoma patients compared to their pre‐treatment counterparts.

## 3 Discussion

**Figure 9:**
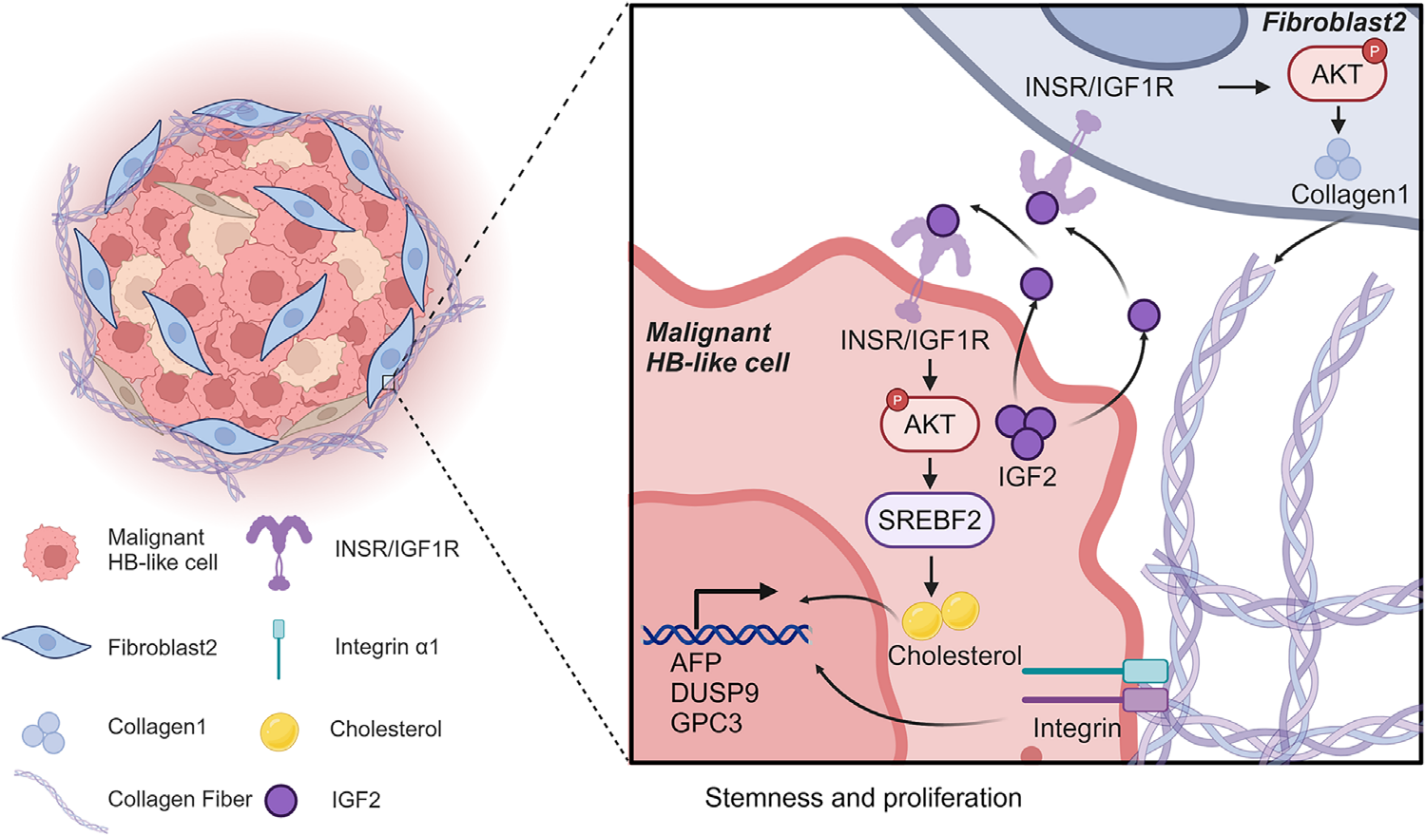
Schematic diagram of malignant HB‐like cells demonstrating self‐sustaining ability via IGF2‐dependent mechanisms in hepatoblastoma.

In this study, we utilized single‐cell RNA sequencing to comprehensively investigate the transcriptomic landscape of human hepatoblastoma. We uncovered a unique IGF2‐related malignant HB‐like cell self‐sustaining ecosystem in primary hepatoblastoma (**Figure** **9**). The identified malignant HB‐like cells generate IGF2 to maintain their stem‐like characteristics by fostering abnormal cholesterol accumulation mediated by SREBF2. We found that IGF2 stimulates fibroblasts to secrete collagen 1, which subsequently intensifies hepatoblastoma malignancy through the collagen 1/integrin α1 signaling axis. Additionally, we found that serum IGF2 levels may serve as a diagnostic biomarker for advanced hepatoblastoma patients.

The present study discovered that hepatocyte malignant HB‐like cells displayed aberrant cholesterol metabolism via activated SREBF2 (Figures 3D and 5A). Cholesterol is synthesized via the mevalonate pathway, an enzymatic cascade mainly controlled by the SREBP family of transcription factors. Mature SREBF migrates into the nucleus and activates the expression of cholesterol biosynthetic genes, such as hydroxyl‐methyl glutaryl‐coenzyme A synthase 1 (HMGCS1).^[^ ^52^ ^]^ Cholesterol, essential for membrane components and metabolites, plays a vital role in the survival and growth of mammalian cells.^[^ ^29^ ^]^ Cholesterol metabolism reprogramming confers tumor ferroptosis resistance, M2 macrophage polarization, and effector T‐cell exhaustion.^[^ ^53^, ^54^ ^]^ Previous studies have demonstrated that high cholesterol levels are significant risk factors in many cancers and are closely associated with tumor malignancy and prognosis.^[^ ^55^, ^56^, ^57^, ^58^, ^59^ ^]^ Preclinical and clinical studies showed that reduced cholesterol metabolism could inhibit tumor growth.^[^ ^56^, ^60^, ^61^ ^]^ Schwabe revealed the mevalonate‐cholesterol‐TAZ‐TEAD2‐Anln/Kif23 pathway as a novel tumor‐specific target for hepatocyte tumor therapy.^[^ ^62^ ^]^ Lin Ng reported the novel role of the Ephrin‐A3/EphA2 axis as a hypoxia‐sensitive modulator of hepatocyte cell cholesterol metabolism and a key target for inhibiting cancer initiation and progression.^[^ ^63^ ^]^ Adult liver tumors are mostly influenced by dietary cholesterol.^[^ ^64^ ^]^ However, the specific mechanism of cholesterol metabolism in hepatoblastoma, a malignant pediatric liver tumor originating from the embryonic liver, has not yet been elucidated. In the present study, we found that the accumulation of cholesterol in hepatoblastoma tumors could be used as a potential prognostic indicator in patients with hepatoblastoma. We also discovered that cholesterol metabolic disorders fuel the proliferation and malignancy of HB‐like cells, highlighting their role in the self‐sustaining mechanism. The study findings could facilitate the development of novel therapeutic strategies for hepatoblastoma from a metabolic perspective.

Multiple oncogenic pathways (AMPK/Wnt/ β‐catenin) have been shown to contribute to hepatoblastoma oncogenesis.^[^ ^23^, ^65^, ^66^ ^]^ The progression of hepatoblastoma is associated with progenitor cells.^[^ ^3^ ^]^ The identified malignant HB‐like cells exhibited liver progenitor cell characteristics. We observed a correlation between malignant HB‐like cells and the survival of patients with hepatoblastoma, suggesting that these cells could predict the prognosis of patients with hepatoblastoma. Furthermore, understanding how normal progenitor cells transform into malignant progenitor cells is crucial for elucidating hepatoblastoma's molecular and cellular foundations. Analysis revealed that signaling pathways, like insulin resistance, AMPK, insulin signaling, Hippo, PPAR, mTOR, and cholesterol metabolism, are enriched during the transition to a CNV highly malignant state. β‐catenin, encoded by CTNNB1, is a key player in hepatoblastoma oncogenesis.^[^ ^67^ ^]^ AMPK, an energy sensor, interacts with β‐catenin to promote cell migration gene transcription.^[^ ^68^ ^]^ PPAR and PI3K/Akt/mTOR pathways regulate critical functions such as proliferation, EMT, metabolism, and angiogenesis.^[^ ^69^, ^70^ ^]^ Additionally, insulin signaling, mTOR signaling, and cholesterol metabolism, which are related to nutrient sensing, are more active in hepatoblastoma.^[^ ^71^, ^72^, ^73^ ^]^ We believe that insulin affects cholesterol metabolism through mTOR/SREBF2 and that nutritional disorders promote proliferation and malignancy in HB‐like cells. These insights deepen our understanding of the molecular basis of malignant HB‐like cell self‐sustaining ability and could aid in developing hepatoblastoma therapies.

IGF2 has a pivotal role in fetal development and could potentially serve as a biomarker for hepatoblastoma diagnosis. IGF2, secreted by malignant HB‐like cells, belongs to the insulin and IGF signaling pathway and functions as an extracellular ligand through autocrine or paracrine mechanisms. IGF2 signaling is primarily transmitted through three receptor complexes on the cell membrane: the IGF1 receptor (IGF1R), the INSR, and the IGF1R‐INSR hybrid receptor. After binding to receptor complexes, IGF2 induces the autophosphorylation of the β‐subunit and activation of the PI3K/Akt pathway.^[^ ^74^ ^]^ Our study found that both malignant HB‐like cells and fibroblast 2 subpopulations exhibited notably high expression of IGF2 receptors (INSR or IGF1R). Further analysis revealed that the PI3K/AKT signaling pathway in these cells was significantly activated (Figure 3; Figure S7, Supporting Information). Based on these observations, we hypothesize that IGF2 is capable of activating malignant HB‐like cells and fibroblast 2 subpopulations. We also noted that the activation of the PI3K/AKT signaling pathway in fibroblasts could lead to an upregulation of collagen 1.^[^ ^46^, ^47^, ^48^ ^]^ Integrating this information, we speculate that collagen 1 secretion by fibroblasts is likely triggered by the paracrine effect of IGF2. This finding provides a new perspective for understanding the self‐sustaining ability of malignant HB‐like cells through a paracrine effect of the IGF2/collagen 1 axis and could impact future treatment strategies.

Our data revealed fibroblast heterogeneity in hepatoblastoma, with fibroblast 2 cells secreting collagen 1 after IGF2‐induced PI3K/AKT activation.^[^ ^45^, ^46^, ^47^, ^48^ ^]^ The interactions between tumor cells and the surrounding stroma play a crucial role in tumorigenesis.^[^ ^42^ ^]^ Among the various cell types that constitute the tumor stroma, fibroblasts have gained significant attention as they serve as both components and regulators.^[^ ^75^, ^76^ ^]^ During tumorigenesis, collagen 1 is often overexpressed and involved in tumor development and progression.^[^ ^44^ ^]^ Multiple studies have highlighted the antitumor potential of targeting collagen 1, further underscoring the pivotal role of collagen 1 in tumor progression. Our findings also demonstrate that IGF2‐induced collagen 1 enhanced hepatoblastoma proliferation and hepatoblast‐related marker expression (Figure 6F–H), indicating its importance in malignant HB‐like cell self‐sustainment.

In summary, single‐cell sequencing showed that malignant HB‐like cells demonstrate an IGF2‐induced self‐sustaining ability in hepatoblastoma. This suggests that targeting malignant HB‐like cells by inhibiting the IGF2‐induced pathway could be a promising therapeutic strategy for hepatoblastoma. Overall, our research provides valuable insights into the genesis and malignancy of hepatoblastoma, potentially paving the way for more effective diagnostic tools and therapeutic strategies for this challenging disease.

## 4 Experimental Section

### Patients and Samples

All samples were obtained from the Shanghai Children's Medical Center. Eight primary hepatoblastoma tumor tissues along with matched 6 adjacent normal liver tissues, were involved in scRNA‐seq. None of the patients had received radiotherapy or chemotherapy prior to surgery. Patients provided informed consent for this work. This study was approved by the Ethics Committee of Shanghai Children's Medical Center of Shanghai Jiao Tong University of Medicine (SCMC1RB‐K2023076‐1). Detailed information of the Chinese hepatoblastoma cohort is provided in Table S1 (Supporting Information).

### Single‐Cell Suspension Preparation

Fresh hepatoblastoma tissues were collected from patients with a surgical scissors. Under aseptic conditions, the tissue was washed twice with pre‐cooled RPMI 1640 + 0.04% BSA medium. Surgical scissors were used to fully cut the tissue to about 0.5 mm^3^ blocks, which were then placed in freshly prepared enzymatic solution and digested in a constant temperature incubator at 37 °C for 30–60 min, with inverse mixing every 5–10 min. The digested cell suspension was filtered using a BD 40 µm cell strainer and then centrifuged at 4 °C and 300 g for 5 min. After suspending the precipitate in moderate medium, an equal volume of red blood cell lysate was added, mixed, and incubated for 10 min before centrifuging at 4 °C and 300 g for 5 min with the supernatant discarded. The precipitate was rinsed in the medium once and then centrifuged at 300 g for 5 min with the supernatant removed. Dead cell removal was performed according to the MACS Dead Cell Removal Kit (130‐090‐101) operating instructions. The cell precipitation was suspended in 100 µL medium, and cell concentration and viability were determined using a Luna fluorometer.

### ScRNA‐Seq Data Processing and Quality Control

The constructed libraries were sequenced using the Illumina Nova seq 6000 PE150 platform. Raw reads generated from high‐throughput sequencing were in FASTQ format files. The quality control of the raw data was assessed and the alignment to the reference genome was performed using CellRanger (10 x Genomics, version 5.0.0). The software effectively quantified high‐throughput single‐cell transcriptome data. It accomplished this by identifying the barcode marker that differentiates cells in the sequence, as well as the UMI marker of distinct mRNA molecules within each cell. This process resulted in the production of quality control statistics, including high‐quality cell counts, gene median value, sequence saturation, among others. The Seurat package was utilized for further quality control with the following steps: Cells were filtered by 1) gene numbers (gene numbers < 200), 2) UMI (UMI <1000), 3) log10 Genes Per UMI (log10 Genes Per UMI < 0.7), 4) percentage of mitochondrial RNA UMIs (proportion of UMIs mapped to mitochondrial genes > 30%) and 5) percentage of hemoglobin RNA UMIs (proportion of UMIs mapped to hemoglobin genes > 5%).^[^ ^77^ ^]^

### Unsupervised Clustering and Differential Expression Gene Analysis

The FindVariableGenes function in the Seurat package was used to screen highly variable genes (HVGs). To remove the batch effects in single‐cell RNA‐sequencing data, the mutual nearest neighbors (MNN) presented by Haghverdi et al was performed with the R package batchelor (version 1.6.3).^[^ ^78^ ^]^ The results were visualized using t‐SNE. Differentially expressed genes (DEGs) were selected using the function FindMarkers (test.use = presto). *p*‐value < 0.05 and log2 fold change > 0.58 was set as the threshold for significantly differential expression. GO enrichment and KEGG pathway enrichment analysis of DEGs were respectively performed using R (version 4.0.3) based on the hypergeometric distribution. The detailed DEG lists of each cell type used in this article can be found in Table S2 (Supporting Information). Gene Set Enrichment Analysis (GSEA) was used to complete GO and KEGG term enrichment analysis with the Molecular Signatures Database (MSigDB) C5 GO gene sets and C2 KEGG gene sets (version 7.2) separately.^[^ ^28^ ^]^ The GSVA package (v1.30.0) was utilized to score the pathway activity of individual cells.^[^ ^79^ ^]^

### Estimation of CNVs in Cells

To identify malignant cells with clonal large‐scale chromosomal copy number variations, the inferCNV (v1.0.4) package was used based on the level of gene expression in the single‐cell transcriptome data.^[^ ^16^ ^]^ The immune cells were designated as normal cells. Genes were sequenced according to chromosome position, and 101 genes were utilized as sliding window size to calculate the average gene expression, and normal cell expression was employed as control.

### Deconvolution Analysis and Integration Analysis

CIBERSORTx was used to perform the deconvolution analysis of the published public data (GSE131329) against the signatures of 3 hepatocyte cell types calculated from the single‐cell RNA‐seq data.^[^ ^25^ ^]^ The microarray data and single‐cell RNA‐seq data were used as the input mixture. The microarray data employed probe signal values and was subjected to quantile normalization for data standardization. In single‐cell RNA‐seq data, the gene expression values of three hepatocyte subpopulations were averaged. The harmony package was employed to remove batch effect for integrated analysis with the public fetal and infant liver single‐cell transcriptome dataset.^[^ ^80^ ^]^

### Pesudotemperal Trajectory Analysis

Pseudotime analysis was performed for cell differentiation trajectory inference using Monocle2 (v2.9.0) R package. The specific steps were as follows: First, the Seurat object was converted into a CellDataSet object using the importCDS function of the Monocle2 package.^[^ ^27^ ^]^ Next, gene ordering was performed by genes that were differentially expressed across clusters and dispersed with a q value < 0.01 using differential Gene Test function, then the reduce Dimension function was used for dimensional reduction and clustering, and finally, the order cells function was used to infer the trajectory of cell differentiation.

### Single‐Cell Regulatory Network Inference and Clustering Analysis (SCENIC)

With default parameters, SCENIC analysis made use of RcisTarget's motifs database and GRNboost (SCENIC v1.1.2.2, RcisTarget v1.2.1, and AUCell v1.4.1).^[^ ^31^ ^]^ The process was as follows: 1) Based on co‐expression, determine each transcription factor's potential target genes. 2) Determine the true transcription factors and their corresponding target genes according to motif analysis by RcisTarget package. 3) The AUCell package was used to grade the level of activity of each regulon in each cell. The regulon specificity score (RSS) based on the Jensen‐Shannon dispersion (JSD) and the connection specificity index (CSI) for all regulons were calculated using the scFunctions (https://github.com/FloWuenne/scFunctions/) package to evaluate the cell type specificity of each regulon.^[^ ^30^ ^]^

### Cell Communication Analysis

CellChat (v1.1.3) package was employed for intercellular ligand‐receptor interaction analysis. ComputeCommunProb, FilterCommunication (min.cells = 10), and ComputeCommunProbPathway functions were used to determine potential receptor‐ligand pairs for subsequent analysis.^[^ ^43^ ^]^ Then aggregateNet function was applied to aggregate the intercellular communication network.

### Immunofluorescence Staining and Evaluation (IHC)

FFPE tissue blocks from hepatoblastoma patients were used for IHC staining, using: anti‐IGF2 (Abcam, ab9574), anti‐DUSP9 (Proteintech, 26718‐1‐AP), BODIPY 493/503 (MCE, 121207‐31‐6), integrin α1 (Cell signal technology, 15574T), collagen 1 (Abcam, 138492), GPC3 (Gentex, GT2473). The staining process was carried out on the IHC/ ISH System (BOND RX, Leica) following the manufacturer's instruction. The multiplex IHC staining method based on tyramine signal amplification (TSA) technology allows the detection of multiple markers on the same tissue slice using different colored dyes. Images were captured using the Aperio Versa digital pathology scanner system according to the manufacturer's protocol. Positive area was calculated by Image J (https://imagej.net/downloads).

### Hepatoblastoma Cell Lines Culture

Human hepatoblastoma cell line HUH6 were purchased from the Cell Bank of Type Culture Collection of the Chinese Academy of Sciences (Shanghai, China). Human hepatoblastoma cell line HepG2 was purchased from the American Type Culture Collection (ATCC) (Maryland, U.S.A). HepG2 cells were cultured in minimum Eagle's medium (MEM), while HUH6 cells were cultured in Dulbecco's modified Eagle's medium (DMEM), with the addition of 10% fetal bovine serum (FBS) and 1% antibiotic, in an incubator with 5% CO_2_ at 37 °C. In experiments using collagen 1, the surfaces of the cell culture plates were coated an acetic acid solution containing with 10 µg mL^−1^ collagen 1 (Sigma, C5483) for 2 h. Subsequently, the plates were washed with Phosphate Buffered Saline (PBS) to remove any excess collagen 1, prior to the seeding of the cells onto the plates.

### Colony Formation Assay and Cell Proliferation Assay

For the colony formation assay, cells were seeded into 12‐well plates at a density of 1000 cells per well. The cells in the plates were incubated for ≈7–10 days in fresh medium, washed with PBS, fixed with 4% PFA, and stained with 0.1% crystal violet. The counts of cell colonies in each well were presented in bar plot finally. For the cell proliferation assay, 1000 cells with the indicated treatment were seeded into 96‐well plates in each well. At the indicated time, the medium of each well was replaced with 100 µL fresh medium supplemented with 10 µL CCK8 reagent (Beyotime, Jiangsu, China), and the cells were incubated for another 2 h. A multiplate reader (Bio Tek, Vermont, USA) was used to determine the absorbance at a wavelength of 450 nm.

### Organoids Culture and Tumorsphere Formation Assay

The hepatoblastoma tissues were minced, digested and then suspended in Matrigel (Corning) and cultured in hepatoblastoma organoid medium (Advanced DMEM/F12 supplemented with Penicillin/Streptomycin, Glutamax, HEPES, B27 supplement, n‐Acetyl‐L‐cysteine, recombinant human R‐Spondin 1, nicotinamide ecombinant human Gastrin I, recombinant human EGF, recombinant human FGF10a and recombinant human HGF). After 5 days, Ac4ManNAz (50 µm) was added to medium for 2 consecutive days. The hepatoblastoma organoids were passaged and recultured for every 7–10 days. Tumorsphere formation was conducted by subculturing hepatoblastoma organoid and HepG2 cells in hepatoblastoma organoid medium. Cell culture was performed in Matrigel or ultra‐low attachment plates. Three thousand cells were plated, and tumorspheres were counted and analyzed after about 7 days.

### Western Blot Assay

Cells were lysed on ice in RIPA lysis buffer (Beyotime) supplemented with phosphatase and protease inhibitors, and then total protein was quantified and boiled in SDS. Afterward, 60 µg of protein samples were separated on an SDS‐PAGE gel and then electrophoretically transferred onto nitrocellulose membranes (GE Healthcare). Then, the membranes were blocked and subsequently incubated with primary antibodies against IGF2 (Abcam, ab9574), Insulin Recepter b (Cell Signal Technology, 23415), phospho‐Insulin Recepter b (Abcam, ab60946), AKT (PTM‐biolab, 6476) pAKT (Cell Signal Technology, 4060T), SQLE (Proteintech,12544‐1‐AP), HMGCS1 (Abcam, ab155787), ERK (PTM‐biolab, 6324), pERK (Cell Signal Technology, 4370T), AFP (PTM‐biolab, 6322), OCT4 (Abcam, ab200834), GPC3 (PTM‐biolab, 5669), DUSP9 (Proteintech, 26718), cMYC (Proteintech, 674471), SREBF2 (Proteintech, 28212), integrin α1 (Cell signal technology, 15574T). Secondary antibodies were fluorescently conjugated (Licor, USA). The bands on the membranes were visualized using an Odyssey instrument (Licor).

### Quantitative Real‐Time PCR Assay

Total RNA was extracted with TRIzol Reagent (Thermo Fisher Scientific;15596‐026) according to the manufacturers’ protocols. RNA was reversely transcripted into cDNA with PrimeScript RT reagent Kit with gDNA Eraser (Takara, RR047A). Quantitative PCR was applied on Bio‐rad CFX‐Connect Real‐Time PCR System with SYBR Green Premix Pro Taq HS qPCR Kit (Accurate Biology, AG11701). mRNA levels were normalized to 18s. qRT‐PCR primer sequences are listed in Table S16 (Supporting Information).

### Oligonucleotide Transfection

Small interfering RNAs (siRNAs) were chemically synthesized by GenePharma. The sequences are provided in Table S17 (Supporting Information). HepG2 and HUH6 cells were transfected with the oligonucleoides using Lipofectamine 2000 (Invitrogen) according to the manufacturer's instructions.

### Enzyme‐Linked Immunosorbent Assay (ELISA)

The IGF2 protein in the collected human serums were confirmed with enzyme‐linked immunosorbent assay (Cusabio, IGF2 human ELISA kit). This assay uses an antibody specific to human IGF2 coated on a 96‐well plate. IGF2 present in a sample was bound to the wells by the immobilized antibody after adding the sample to the wells. Biotinylated IGF2 antibody was added to the wells, forming an antibody‐analyte‐antibody complex. HRP‐conjugated avidin was pipetted to the wells and binds to the complex. A TMB substrate solution was added to the wells and color develops in proportion to the amount of IGF2 bound. The addition of Stop Solution changes the color from blue to yellow, and the intensity of the color was measured at 450 nm using a microplate reader.

### Mice and Treatments

Nude mice were purchased from Shanghai Model Organisms and were maintained in the animal facilities of Shanghai Children's Medical Center. The animal experiment was also approved by the Ethics Committee of Shanghai Children's Medical Center (SCMC‐LAWEC‐2021‐111). High cholesterol Diet (HCD) was achieved by feeding mice a dietary chow consisting of 1.5% cholesterol beginning at the age of 4–17 weeks. Control mice were sex‐ and age‐matched and fed standard chow ad libitum. The initial tumor implantation was conducted in animals at the age of 6–8 weeks. The cells inoculated subcutaneously were mixed with a neutralized collagen 1 solution (1 mg mL^−1^) when needed. Fatostain (Medchemexpress, MCE) was reconstituted in a solution of 45% PEG400 (Medchemexpress, MCE), 5% Tween80 (Medchemexpress, MCE), 50% physiological saline for a daily intraperitoneal injection of 15 mg kg^−1^. Tumor heights and widths were measured with a caliper every 3–4 days to calculate tumor volume (width^2^ × height × 0.5). Mice were bled when sacrificed and the concentrations of cholesterol in sera were detected with total cholesterol assay kit (Nanjing Jiancheng Bioengineering Institute).

### Statistical Analysis

The quantitative data obtained from three distinct experiments were presented as the mean ± standard deviation (SD). To quantify the fluorescence intensities of IGF2, SREBF2, BODIPY, DUSP9, collagen 1, and integrin α1, 3–5 representative images for each sample were analyzed using ImageJ software. Colocalization analysis was also conducted using ImageJ. For statistical analysis, SPSS 23.0 was employed, utilizing Pearson's χ^2^ test and Fisher's exact test to determine significant differences among grouped samples. GraphPad Prism 8.0 was used to perform Student's two‐tailed unpaired t‐test for pairwise comparisons, one‐way analysis of variance (ANOVA) for multiple comparisons, and two‐way ANOVA for comparisons involving two independent variables. Pearson's Correlation analysis was applied to calculate the correlation coefficient.

## Conflict of Interest

The authors declare no conflict of interest.

## Author Contributions

M.D., S.M., and H.W. contributed equally to this work. J.M., Q.P., and G.Z. contributed equally to this work. M.D., J.M., and G.Z. designed this study. Q.P. and F.S. supervised this study. S.M., T.C., and X.W. coordinated the sample collection. M.D., S.M., J.Z., and S.F. conducted IHC and in vitro experiments. N.Z. and X.T. performed H&E staining. M.D., G.Z., and H.W. conducted the data analysis. M.D. and H.W. wrote the original manuscript. J.M., Q.P., and G.Z. reviewed and polished the manuscript.

## Supporting information



Supporting Information

## Data Availability

The raw sequence data reported in this paper have been deposited in the Genome Sequence Archive (Genomics, Proteomics & Bioinformatics 2021) in National Genomics Data Center (Nucleic Acids Res 2022), China National Center for Bioinformation / Beijing Institute of Genomics, Chinese Academy of Sciences (GSA‐Human: HRA007089) that are publicly accessible at https://ngdc.cncb.ac.cn/gsa‐human. The data will be released on April 4, 2026.

## References

[1] G. Digiacomo , R. P. Serra , E. Turrini , A. Tiri , A. Cavazzoni , R. Alfieri , P. Bertolini , Biochem. Pharmacol. 2023, 207, 115373. 36513143 10.1016/j.bcp.2022.115373

[2] G. P. Fragulidis , K. Chondrogiannis , A. Vezakis , A. Melemeni , A. Kondi‐Pafiti , E. Primetis , A. Polydorou , D. C. Voros , Hepatol. Res. 2013, 43, 320. 23437913 10.1111/j.1872-034X.2012.01070.x

[3] D. Sia , A. Villanueva , S. L. Friedman , J. M. Llovet , Gastroenterology 2017, 152, 745. 28043904 10.1053/j.gastro.2016.11.048 PMC12160040

[4] L. Zhou , K. H. Yu , T. L. Wong , Z. Zhang , C. H. Chan , J. H. Loong , N. Che , H. J. Yu , K. V. Tan , M. Tong , E. S. Ngan , J. W. Ho , S. Ma , Gut 2022, 71, 1656. 34588223 10.1136/gutjnl-2021-324321

[5] M. Honda , K. Uchida , T. Irie , K. Hirukawa , M. Kadohisa , K. Shimata , K. Isono , N. Shimojima , Y. Sugawara , T. Hibi , Cancer Med. 2023, 12, 3909. 36394165 10.1002/cam4.5300 PMC9972171

[6] P. V. Wu , A. Rangaswami , Curr. Oncol. Rep. 2022, 24, 1209. 35438389 10.1007/s11912-022-01230-2

[7] K. E. de Visser , J. A. Joyce , Cancer Cell 2023, 41, 374. 36917948 10.1016/j.ccell.2023.02.016

[8] L. Fan , Q. Pan , W. Yang , S. C. Koo , C. Tian , L. Li , M. Lu , A. Brown , B. Ju , J. Easton , S. Ranganathan , S. Shin , A. Bondoc , J. J. Yang , J. Yu , L. Zhu , Hepatology 2022, 76, 1275. 35179799 10.1002/hep.32412 PMC9385889

[9] H. Huang , L. Wu , L. Lu , Z. Zhang , B. Qiu , J. Mo , Y. Luo , Z. Xi , M. Feng , P. Wan , J. Zhu , D. Yu , W. Wu , K. Tan , J. Liu , Q. Sheng , T. Xu , J. Huang , Z. Lv , Y. Tang , Q. Xia , Hepatology 2023, 77, 1911. 36059151 10.1002/hep.32775

[10] Q. S. Zuo , R. Yan , D. X. Feng , R. Zhao , C. Chen , Y. M. Jiang , M. Cruz‐Correa , A. G. Casson , X. D. Kang , F. Han , T. Chen , Mol. Carcinog. 2011, 50, 390. 21268128 10.1002/mc.20731

[11] Y. Zhang , H. Yan , Y. Jiang , T. Chen , Z. Ma , F. Li , M. Lin , Y. Xu , X. Zhang , J. Zhang , H. He , Exp. Biol. Med. (Maywood) 2021, 246, 371. 33175607 10.1177/1535370220966253 PMC7885054

[12] Y. Q. Gao , H. Y. Cheng , K. F. Liu , Eur. Rev. Med. Pharmacol. Sci. 2020, 24, 9239. 33015760 10.26355/eurrev_202009_22998

[13] J. Sun , J. Shu , D. Shi , W. Liu , Y. Zhang , B. Luo , Cancer Biomark. 2023, 38, 355. 37718779 10.3233/CBM-230105 PMC12412869

[14] G. Nagae , S. Yamamoto , M. Fujita , T. Fujita , A. Nonaka , T. Umeda , S. Fukuda , K. Tatsuno , K. Maejima , A. Hayashi , S. Kurihara , M. Kojima , T. Hishiki , K. Watanabe , K. Ida , M. Yano , Y. Hiyama , Y. Tanaka , T. Inoue , H. Ueda , H. Nakagawa , H. Aburatani , E. Hiyama , Nat. Commun. 2021, 12, 5423. 34538872 10.1038/s41467-021-25430-9 PMC8450290

[15] J. Abril‐Fornaguera , L. Torrens , C. Andreu‐Oller , J. Carrillo‐Reixach , A. Rialdi , U. Balaseviciute , R. Pinyol , C. Montironi , P. K. Haber , A. Del Rio‐Alvarez , M. Domingo‐Sabat , L. Royo , N. K. Akers , C. E. Willoughby , J. Peix , M. Torres‐Martin , M. Puigvehi , S. Cairo , M. Childs , R. Maibach , R. Alaggio , P. Czauderna , B. Morland , B. Losic , V. Mazzaferro , E. Guccione , D. Sia , C. Armengol , J. M. Llovet , Mol. Cancer Ther. 2023, 22, 485. 36780225 10.1158/1535-7163.MCT-22-0335 PMC10073300

[16] S. V. Puram , I. Tirosh , A. S. Parikh , A. P. Patel , K. Yizhak , S. Gillespie , C. Rodman , C. L. Luo , E. A. Mroz , K. S. Emerick , D. G. Deschler , M. A. Varvares , R. Mylvaganam , O. Rozenblatt‐Rosen , J. W. Rocco , W. C. Faquin , D. T. Lin , A. Regev , B. E. Bernstein , Cell 2017, 171, 1611. 29198524 10.1016/j.cell.2017.10.044 PMC5878932

[17] L. Zhang , N. Theise , M. Chua , L. M. Reid , Hepatology 2008, 48, 1598. 18972441 10.1002/hep.22516

[18] Y. Takashima , M. Terada , M. Udono , S. Miura , J. Yamamoto , A. Suzuki , Hepatology 2016, 64, 245. 26990797 10.1002/hep.28548

[19] E. Schmelzer , Differentiation 2019, 106, 9. 30826473 10.1016/j.diff.2019.02.005

[20] S. Cairo , C. Armengol , A. De Reynies , Y. Wei , E. Thomas , C. A. Renard , A. Goga , A. Balakrishnan , M. Semeraro , L. Gresh , M. Pontoglio , H. Strick‐Marchand , F. Levillayer , Y. Nouet , D. Rickman , F. Gauthier , S. Branchereau , L. Brugieres , V. Laithier , R. Bouvier , F. Boman , G. Basso , J. F. Michiels , P. Hofman , F. Arbez‐Gindre , H. Jouan , M. C. Rousselet‐Chapeau , D. Berrebi , L. Marcellin , F. Plenat , et al., Cancer Cell 2008, 14, 471. 19061838 10.1016/j.ccr.2008.11.002

[21] A. Roehrig , T. Z. Hirsch , A. Pire , G. Morcrette , B. Gupta , C. Marcaillou , S. Imbeaud , C. Chardot , E. Gonzales , E. Jacquemin , M. Sekiguchi , J. Takita , G. Nagae , E. Hiyama , F. Guerin , M. Fabre , I. Aerts , S. Taque , V. Laithier , S. Branchereau , C. Guettier , L. Brugieres , B. Fresneau , J. Zucman‐Rossi , E. Letouze , Nat. Commun. 2024, 15, 3031. 38589411 10.1038/s41467-024-47280-x PMC11001886

[22] A. Bondoc , K. Glaser , K. Jin , C. Lake , S. Cairo , J. Geller , G. Tiao , B. Aronow , Commun. Biol. 2021, 4, 1049. 34497364 10.1038/s42003-021-02562-8 PMC8426487

[23] T. Z. Hirsch , J. Pilet , G. Morcrette , A. Roehrig , B. J. E. Monteiro , L. Molina , Q. Bayard , E. Trepo , L. Meunier , S. Caruso , V. Renault , J. F. Deleuze , B. Fresneau , C. Chardot , E. Gonzales , E. Jacquemin , F. Guerin , M. Fabre , I. Aerts , S. Taque , V. Laithier , S. Branchereau , C. Guettier , L. Brugieres , S. Rebouissou , E. Letouze , J. Zucman‐Rossi , Cancer Discovery 2021, 11, 2524. 33893148 10.1158/2159-8290.CD-20-1809 PMC8916021

[24] K. B. Hooks , J. Audoux , H. Fazli , S. Lesjean , T. Ernault , N. Dugot‐Senant , T. Leste‐Lasserre , M. Hagedorn , B. Rousseau , C. Danet , S. Branchereau , L. Brugieres , S. Taque , C. Guettier , M. Fabre , A. Rullier , M. A. Buendia , T. Commes , C. F. Grosset , A. A. Raymond , Hepatology 2018, 68, 89. 29152775 10.1002/hep.29672

[25] A. M. Newman , C. B. Steen , C. L. Liu , A. J. Gentles , A. A. Chaudhuri , F. Scherer , M. S. Khodadoust , M. S. Esfahani , B. A. Luca , D. Steiner , M. Diehn , A. A. Alizadeh , Nat. Biotechnol. 2019, 37, 773. 31061481 10.1038/s41587-019-0114-2 PMC6610714

[26] X. Wang , L. Yang , Y. C. Wang , Z. R. Xu , Y. Feng , J. Zhang , Y. Wang , C. R. Xu , Cell Res. 2020, 30, 1109. 32690901 10.1038/s41422-020-0378-6 PMC7784864

[27] C. Trapnell , D. Cacchiarelli , J. Grimsby , P. Pokharel , S. Li , M. Morse , N. J. Lennon , K. J. Livak , T. S. Mikkelsen , J. L. Rinn , Nat. Biotechnol. 2014, 32, 381. 24658644 10.1038/nbt.2859 PMC4122333

[28] A. Subramanian , P. Tamayo , V. K. Mootha , S. Mukherjee , B. L. Ebert , M. A. Gillette , A. Paulovich , S. L. Pomeroy , T. R. Golub , E. S. Lander , J. P. Mesirov , Proc. Natl. Acad. Sci. USA 2005, 102, 15545. 16199517 10.1073/pnas.0506580102 PMC1239896

[29] J. Luo , H. Yang , B. L. Song , Nat. Rev. Mol. Cell Biol. 2020, 21, 225. 31848472 10.1038/s41580-019-0190-7

[30] S. Suo , Q. Zhu , A. Saadatpour , L. Fei , G. Guo , G. C. Yuan , Cell Rep. 2018, 25, 1436. 30404000 10.1016/j.celrep.2018.10.045 PMC6281296

[31] S. Aibar , C. B. Gonzalez‐Blas , T. Moerman , V. A. Huynh‐Thu , H. Imrichova , G. Hulselmans , F. Rambow , J. C. Marine , P. Geurts , J. Aerts , J. van den Oord , Z. K. Atak , J. Wouters , S. Aerts , Nat. Methods 2017, 14, 1083. 28991892 10.1038/nmeth.4463 PMC5937676

[32] G. T. Bommer , O. A. MacDougald , Cell Metab. 2011, 13, 241. 21356514 10.1016/j.cmet.2011.02.004 PMC3062104

[33] L. Bejarano , M. J. C. Jordao , J. A. Joyce , Cancer Discovery 2021, 11, 933. 33811125 10.1158/2159-8290.CD-20-1808

[34] A. De Boeck , P. Pauwels , K. Hensen , J. L. Rummens , W. Westbroek , A. Hendrix , D. Maynard , H. Denys , K. Lambein , G. Braems , C. Gespach , M. Bracke , O. De Wever , Gut 2013, 62, 550. 22535374 10.1136/gutjnl-2011-301393

[35] G. Ren , X. Zhao , Y. Wang , X. Zhang , X. Chen , C. Xu , Z. R. Yuan , A. I. Roberts , L. Zhang , B. Zheng , T. Wen , Y. Han , A. B. Rabson , J. A. Tischfield , C. Shao , Y. Shi , Cell Stem Cell 2012, 11, 812. 23168163 10.1016/j.stem.2012.08.013 PMC3518598

[36] B. H. Jenkins , J. F. Buckingham , C. J. Hanley , G. J. Thomas , Pharmacol. Ther. 2022, 240, 108231. 35718294 10.1016/j.pharmthera.2022.108231

[37] O. Strauss , A. Phillips , K. Ruggiero , A. Bartlett , P. R. Dunbar , Sci. Rep. 2017, 7, 44356. 28287163 10.1038/srep44356 PMC5347010

[38] S. A. MacParland , J. C. Liu , X. Z. Ma , B. T. Innes , A. M. Bartczak , B. K. Gage , J. Manuel , N. Khuu , J. Echeverri , I. Linares , R. Gupta , M. L. Cheng , L. Y. Liu , D. Camat , S. W. Chung , R. K. Seliga , Z. Shao , E. Lee , S. Ogawa , M. Ogawa , M. D. Wilson , J. E. Fish , M. Selzner , A. Ghanekar , D. Grant , P. Greig , G. Sapisochin , N. Selzner , N. Winegarden , O. Adeyi , et al., Nat. Commun. 2018, 9, 4383. 30348985 10.1038/s41467-018-06318-7 PMC6197289

[39] C. Wei , C. Yang , S. Wang , D. Shi , C. Zhang , X. Lin , Q. Liu , R. Dou , B. Xiong , Mol. Cancer 2019, 18, 64. 30927925 10.1186/s12943-019-0976-4 PMC6441214

[40] E. Azizi , A. J. Carr , G. Plitas , A. E. Cornish , C. Konopacki , S. Prabhakaran , J. Nainys , K. Wu , V. Kiseliovas , M. Setty , K. Choi , R. M. Fromme , P. Dao , P. T. McKenney , R. C. Wasti , K. Kadaveru , L. Mazutis , A. Y. Rudensky , D. Pe'er , Cell 2018, 174, 1293. 29961579 10.1016/j.cell.2018.05.060 PMC6348010

[41] D. H. Aggen , C. R. Ager , A. Z. Obradovic , N. Chowdhury , A. Ghasemzadeh , W. Mao , M. G. Chaimowitz , Z. A. Lopez‐Bujanda , C. S. Spina , J. E. Hawley , M. C. Dallos , C. Zhang , V. Wang , H. Li , X. V. Guo , C. G. Drake , Clin. Cancer Res. 2021, 27, 608. 33148676 10.1158/1078-0432.CCR-20-1610 PMC7980495

[42] D. C. Hinshaw , L. A. Shevde , Cancer Res. 2019, 79, 4557. 31350295 10.1158/0008-5472.CAN-18-3962 PMC6744958

[43] S. Jin , C. F. Guerrero‐Juarez , L. Zhang , I. Chang , R. Ramos , C. H. Kuan , P. Myung , M. V. Plikus , Q. Nie , Nat. Commun. 2021, 12, 1088. 33597522 10.1038/s41467-021-21246-9 PMC7889871

[44] D. De Martino , J. J. Bravo‐Cordero , Cancer Res. 2023, 83, 1386. 36638361 10.1158/0008-5472.CAN-22-2034 PMC10159947

[45] G. R. Grotendorst , H. Rahmanie , M. R. Duncan , FASEB J. 2004, 18, 469. 15003992 10.1096/fj.03-0699com

[46] X. Hu , Q. Xu , H. Wan , Y. Hu , S. Xing , H. Yang , Y. Gao , Z. He , Lab. Invest. 2020, 100, 801. 32051533 10.1038/s41374-020-0404-9

[47] P. G. Murphy , B. J. Loitz , C. B. Frank , D. A. Hart , Biochem. Cell Biol. 1994, 72, 403. 7605612 10.1139/o94-054

[48] Q. Zou , X. Wang , R. Yuan , Z. Gong , C. Luo , Y. Xiong , Y. Jiang , Int. J. Biol. Macromol. 2023, 226, 357. 36502948 10.1016/j.ijbiomac.2022.12.029

[49] J. Cooper , F. G. Giancotti , Cancer Cell 2019, 35, 347. 30889378 10.1016/j.ccell.2019.01.007 PMC6684107

[50] C. Li , S. Qiu , X. Liu , F. Guo , J. Zhai , Z. Li , L. Deng , L. Ge , H. Qian , L. Yang , B. Xu , Signal Transduct. Target Ther. 2023, 8, 247. 37369642 10.1038/s41392-023-01453-0 PMC10300038

[51] T. Malati , Clin. Biochem. 2014, 47, 734. 24854689 10.1016/j.clinbiochem.2014.05.035

[52] M. Jamal , Y. Lei , H. He , X. Zeng , H. I. Bangash , D. Xiao , L. Shao , F. Zhou , Q. Zhang , Front. Pharmacol. 2023, 14, 1257289. 37745085 10.3389/fphar.2023.1257289 PMC10512069

[53] J. Huang , H. Pan , J. Sun , J. Wu , Q. Xuan , J. Wang , S. Ke , S. Lu , Z. Li , Z. Feng , Y. Hua , Q. Yu , B. Yin , B. Qian , M. Zhou , Y. Xu , M. Bai , Y. Zhang , Y. Wu , Y. Ma , H. Jiang , W. Dai , J. Exp. Clin. Cancer Res. 2023, 42, 286. 37891677 10.1186/s13046-023-02865-0 PMC10612308

[54] S. Wang , R. Wang , N. Xu , X. Wei , Y. Yang , Z. Lian , B. Cen , C. Shen , W. Li , J. Wang , Z. Zhang , L. Tang , Q. Wei , D. Lu , X. Xu , Hepatology 2023, 78, 1064. 36626623 10.1097/HEP.0000000000000025

[55] E. R. Nelson , S. E. Wardell , J. S. Jasper , S. Park , S. Suchindran , M. K. Howe , N. J. Carver , R. V. Pillai , P. M. Sullivan , V. Sondhi , M. Umetani , J. Geradts , D. P. McDonnell , Science 2013, 342, 1094. 24288332 10.1126/science.1241908 PMC3899689

[56] B. Huang , B. L. Song , C. Xu , Nat. Metab. 2020, 2, 132. 32694690 10.1038/s42255-020-0174-0

[57] J. Hoppstadter , A. Dembek , M. Horing , H. S. Schymik , C. Dahlem , A. Sultan , N. Wirth , S. Al‐Fityan , B. Diesel , G. Gasparoni , J. Walter , V. Helms , H. Huwer , M. Simon , G. Liebisch , M. H. Schulz , A. K. Kiemer , EBioMedicine 2021, 72, 103578. 34571364 10.1016/j.ebiom.2021.103578 PMC8479395

[58] A. Ioannidou , E. L. Watts , A. Perez‐Cornago , E. A. Platz , I. G. Mills , T. J. Key , R. C. Travis , C. B. C. P. Practical consortium , K. K. Tsilidis , V. Zuber , PLoS Med. 2022, 19, e1003859. 35085228 10.1371/journal.pmed.1003859 PMC8794090

[59] X. Wang , B. Sun , L. Wei , X. Jian , K. Shan , Q. He , F. Huang , X. Ge , X. Gao , N. Feng , Y. Q. Chen , Neoplasia 2022, 24, 86. 34954451 10.1016/j.neo.2021.11.004 PMC8718564

[60] O. F. Kuzu , M. A. Noory , G. P. Robertson , Cancer Res. 2016, 76, 2063. 27197250 10.1158/0008-5472.CAN-15-2613 PMC5813477

[61] H. Xu , S. Zhou , Q. Tang , H. Xia , F. Bi , Biochim. Biophys. Acta Rev. Cancer 2020, 1874, 188394. 32698040 10.1016/j.bbcan.2020.188394

[62] Y. Saito , D. Yin , N. Kubota , X. Wang , A. Filliol , H. Remotti , A. Nair , L. Fazlollahi , Y. Hoshida , I. Tabas , K. J. Wangensteen , R. F. Schwabe , Gastroenterology 2023, 164, 1279. 36894036 10.1053/j.gastro.2023.02.043 PMC10335360

[63] A. Husain , Y. T. Chiu , K. M. Sze , D. W. Ho , Y. M. Tsui , E. M. S. Suarez , V. X. Zhang , L. K. Chan , E. Lee , J. M. Lee , T. T. Cheung , C. C. Wong , C. Y. Chung , I. O. Ng , J. Hepatol. 2022, 77, 383. 35227773 10.1016/j.jhep.2022.02.018

[64] X. Zhang , O. O. Coker , E. S. Chu , K. Fu , H. C. H. Lau , Y. X. Wang , A. W. H. Chan , H. Wei , X. Yang , J. J. Y. Sung , J. Yu , Gut 2021, 70, 761. 32694178 10.1136/gutjnl-2019-319664 PMC7948195

[65] W. Jia , L. Zhong , Q. Ren , D. Teng , L. Gong , H. Dong , J. Li , C. Wang , Y. X. He , J. Yang , Environ. Res. 2024, 249, 118402. 38309560 10.1016/j.envres.2024.118402

[66] Q. Wang , N. Liang , T. Yang , Y. Li , J. Li , Q. Huang , C. Wu , L. Sun , X. Zhou , X. Cheng , L. Zhao , G. Wang , Z. Chen , X. He , C. Liu , J. Hepatol. 2021, 75, 1142. 34217777 10.1016/j.jhep.2021.06.025

[67] V. Preda , S. J. Larkin , N. Karavitaki , O. Ansorge , A. B. Grossman , Endocr. Pathol. 2015, 26, 1. 25355426 10.1007/s12022-014-9341-8

[68] J. Chen , Q. Zhou , J. Feng , W. Zheng , J. Du , X. Meng , Y. Wang , J. Wang , Life Sci. 2019, 239, 116877. 31669575 10.1016/j.lfs.2019.116877

[69] A. Vallee , Y. Lecarpentier , Front. Immunol. 2018, 9, 745. 29706964 10.3389/fimmu.2018.00745 PMC5908886

[70] A. Barzegar Behrooz , Z. Talaie , F. Jusheghani , M. J. Los , T. Klonisch , S. Ghavami , Int. J. Mol. Sci. 2022, 23, 1353. 35163279 10.3390/ijms23031353 PMC8836096

[71] E. Larque , M. Ruiz‐Palacios , B. Koletzko , Curr. Opin. Clin. Nutr. Metab. Care 2013, 16, 292. 23416721 10.1097/MCO.0b013e32835e3674

[72] V. Panwar , A. Singh , M. Bhatt , R. K. Tonk , S. Azizov , A. S. Raza , S. Sengupta , D. Kumar , M. Garg , Signal Transduct. Target Ther. 2023, 8, 375. 37779156 10.1038/s41392-023-01608-z PMC10543444

[73] N. M. Templeman , C. T. Murphy , J. Cell Biol. 2018, 217, 93. 29074705 10.1083/jcb.201707168 PMC5748989

[74] C. Selenou , F. Brioude , E. Giabicani , M. L. Sobrier , I. Netchine , Cells 2022, 11, 1886. 35741015 10.3390/cells11121886 PMC9221339

[75] Y. Chen , K. M. McAndrews , R. Kalluri , Nat. Rev. Clin. Oncol. 2021, 18, 792. 34489603 10.1038/s41571-021-00546-5 PMC8791784

[76] S. Kidd , E. Spaeth , J. L. Dembinski , M. Dietrich , K. Watson , A. Klopp , V. L. Battula , M. Weil , M. Andreeff , F. C. Marini , Stem Cells 2009, 27, 2614. 19650040 10.1002/stem.187 PMC4160730

[77] C. S. McGinnis , L. M. Murrow , Z. J. Gartner , Cell Syst. 2019, 8, 329. 30954475 10.1016/j.cels.2019.03.003 PMC6853612

[78] L. Haghverdi , A. T. L. Lun , M. D. Morgan , J. C. Marioni , Nat. Biotechnol. 2018, 36, 421. 29608177 10.1038/nbt.4091 PMC6152897

[79] S. Hanzelmann , R. Castelo , J. Guinney , BMC Bioinformatics 2013, 14, 7. 23323831 10.1186/1471-2105-14-7 PMC3618321

[80] H. T. N. Tran , K. S. Ang , M. Chevrier , X. Zhang , N. Y. S. Lee , M. Goh , J. Chen , Genome Biol. 2020, 21, 12. 31948481 10.1186/s13059-019-1850-9 PMC6964114

