# Supplementary material for: Malignant Hepatoblast‐Like Cells Sustain Stemness via IGF2‐Dependent Cholesterol Accumulation in Hepatoblastoma
Source: Adv Sci (Weinh). 2025 Apr 24;12(20):2407671. doi: 10.1002/advs.202407671 (PMC12120738; doi:10.1002/advs.202407671)
Supplement: Supplementary file 1 — Supporting Information [file ADVS-12-2407671-s001.docx]

Supporting Information

Malignant Hepatoblast-like Cells Sustain Stemness via IGF2-dependent Cholesterol Accumulation in Hepatoblastoma

Author(s), and Corresponding Author(s)*

Miao Ding, Siwei Mao, Han Wu, Sijia Fang, Ni Zhen, Tianshu Chen, Jiabei Zhu, Xiaochen Tang, Xiaoyang Wang, Feiyong Sun, Guoqing Zhu*, Qiuhui Pan*, Ji Ma*


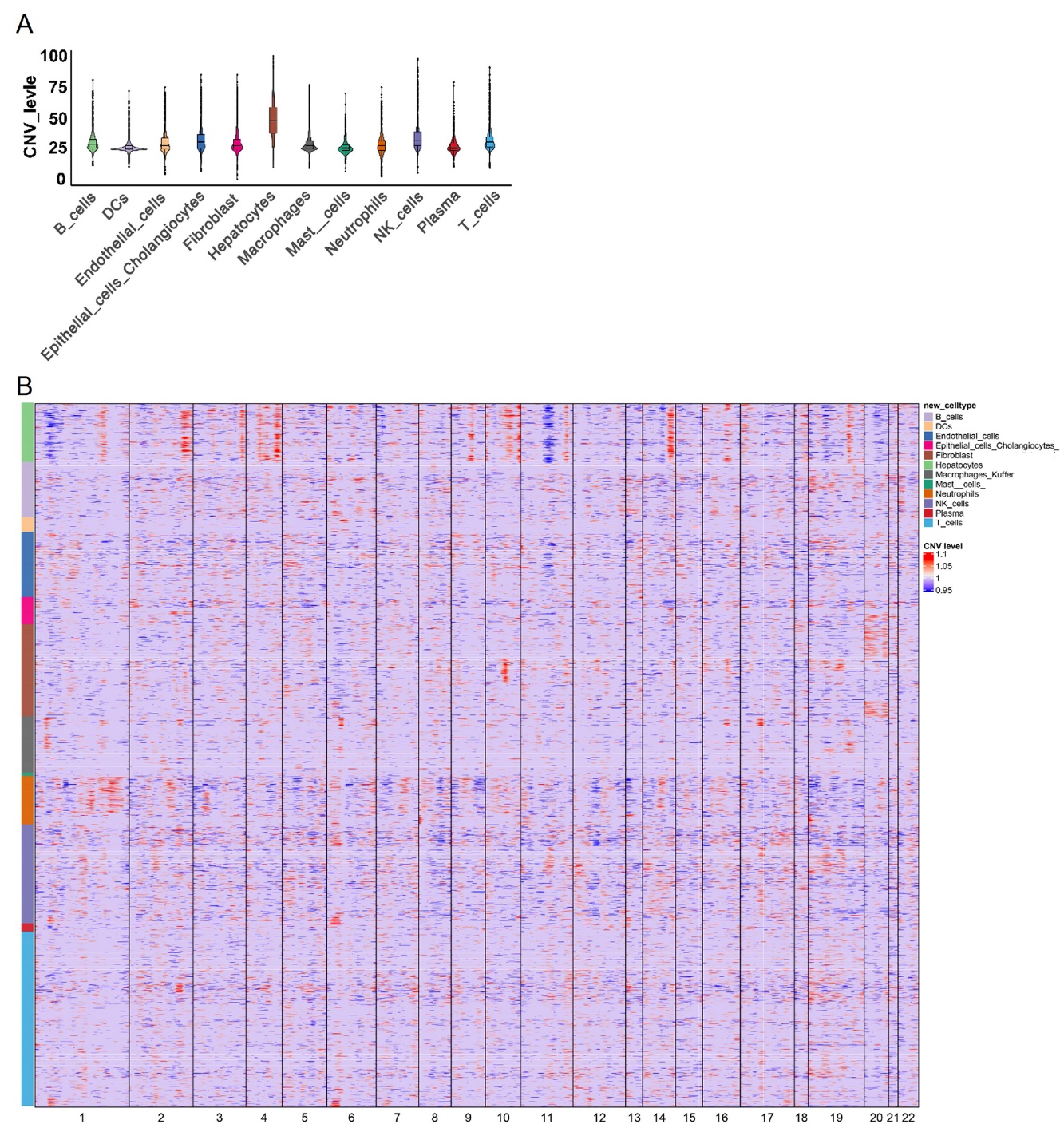


**Figure S1. The predicted inferCNV results for 12 major cell types in primary hepatoblastoma tumors and paired paracancerous normal liver tissues.**

1. The violin plot shows the predicted inferCNV results. B. Heatmap shows the predicted inferCNV results of 12 major cell types.


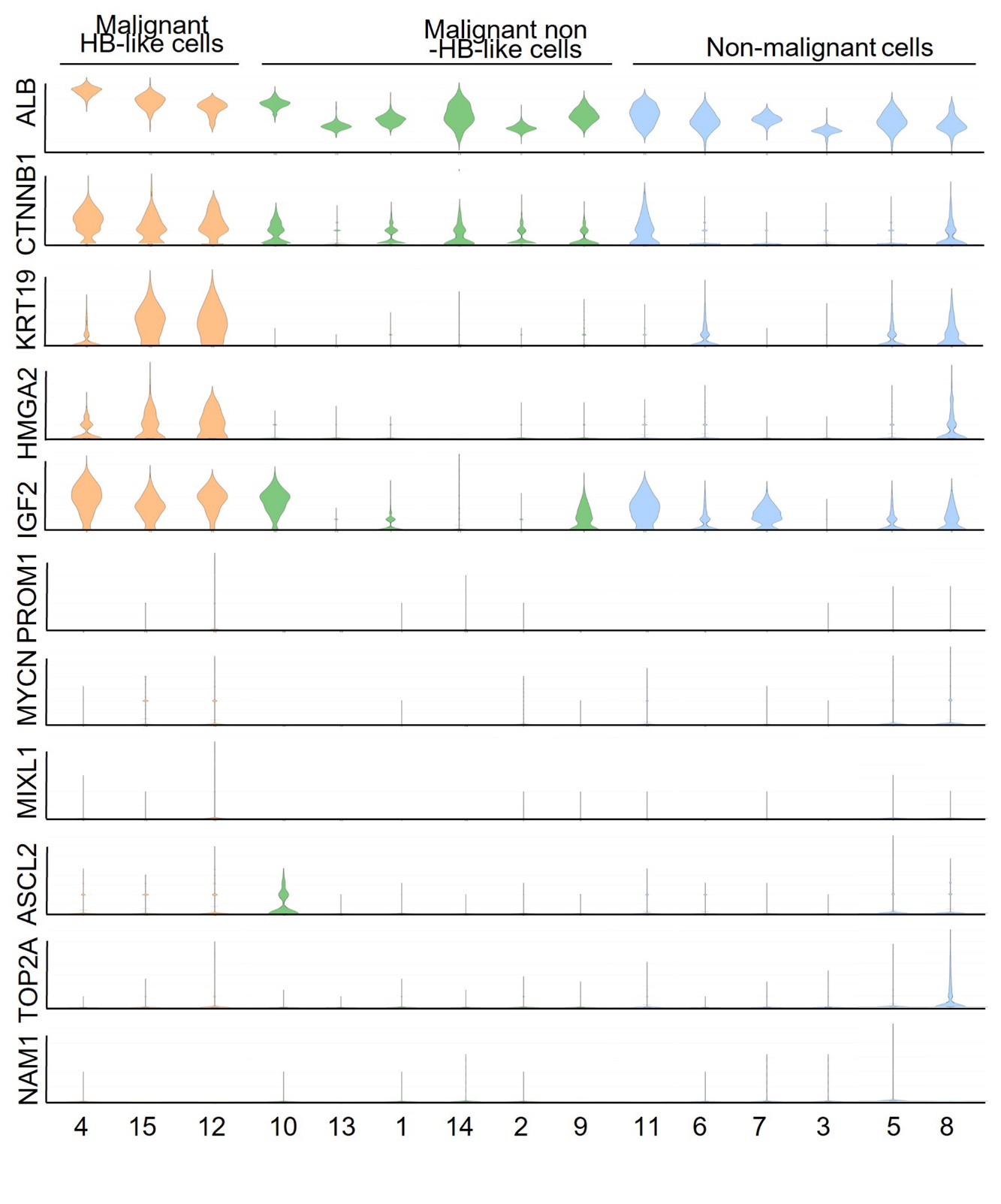


**Figure S2. Violin plots showing the expression levels of hepatoblast-associated genes in 15 hepatocyte clusters.**


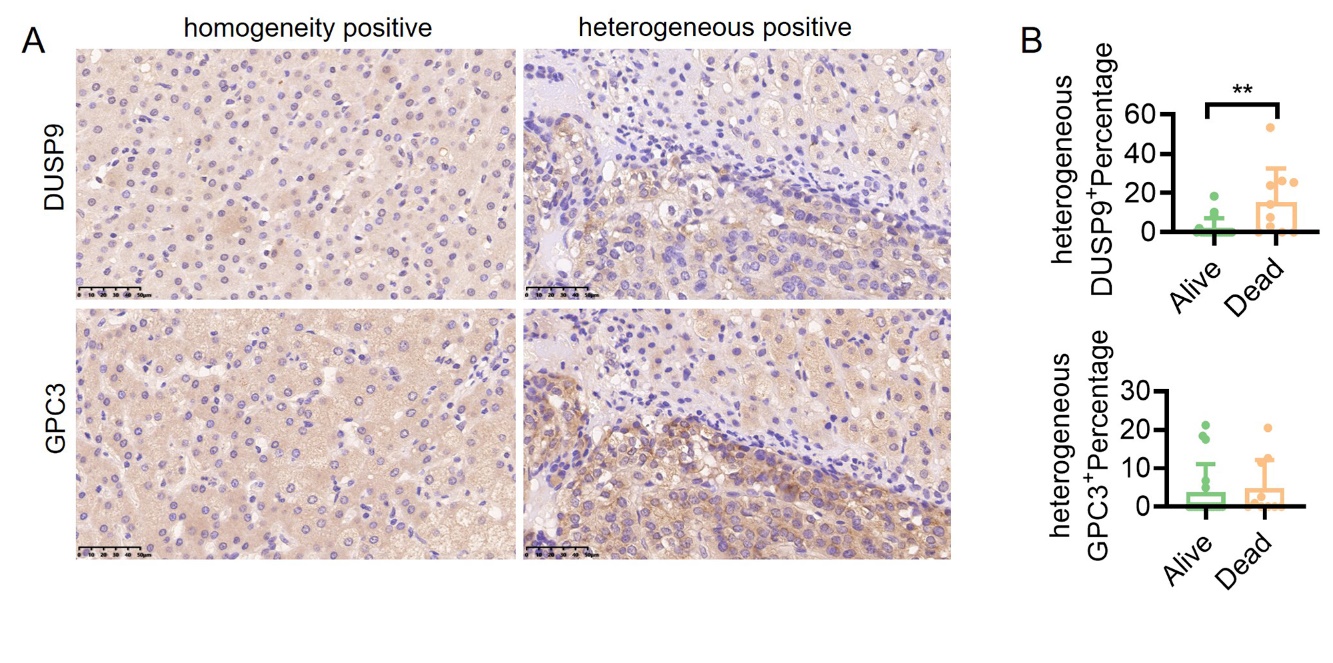


**Figure S3. Identification and statistical analysis of correlations between the proportions of heterogeneous DUSP9/ GPC3-positive cells in hepatoblastoma tissues and clinical course.**

A. Representative immunohistological images of homogeneous and heterogeneous DUSP9/GPC3-positive cells in human hepatoblastoma tissues. B. The proportion of heterogeneous DUSP9/GPC3-positive cells in hepatoblastoma tissues from different clinical course patients. C.Statistical analysis of the correlations between the proportion of heterogeneous DUSP9/GPC3-positive cells in hepatoblastoma tissues and clinical course.


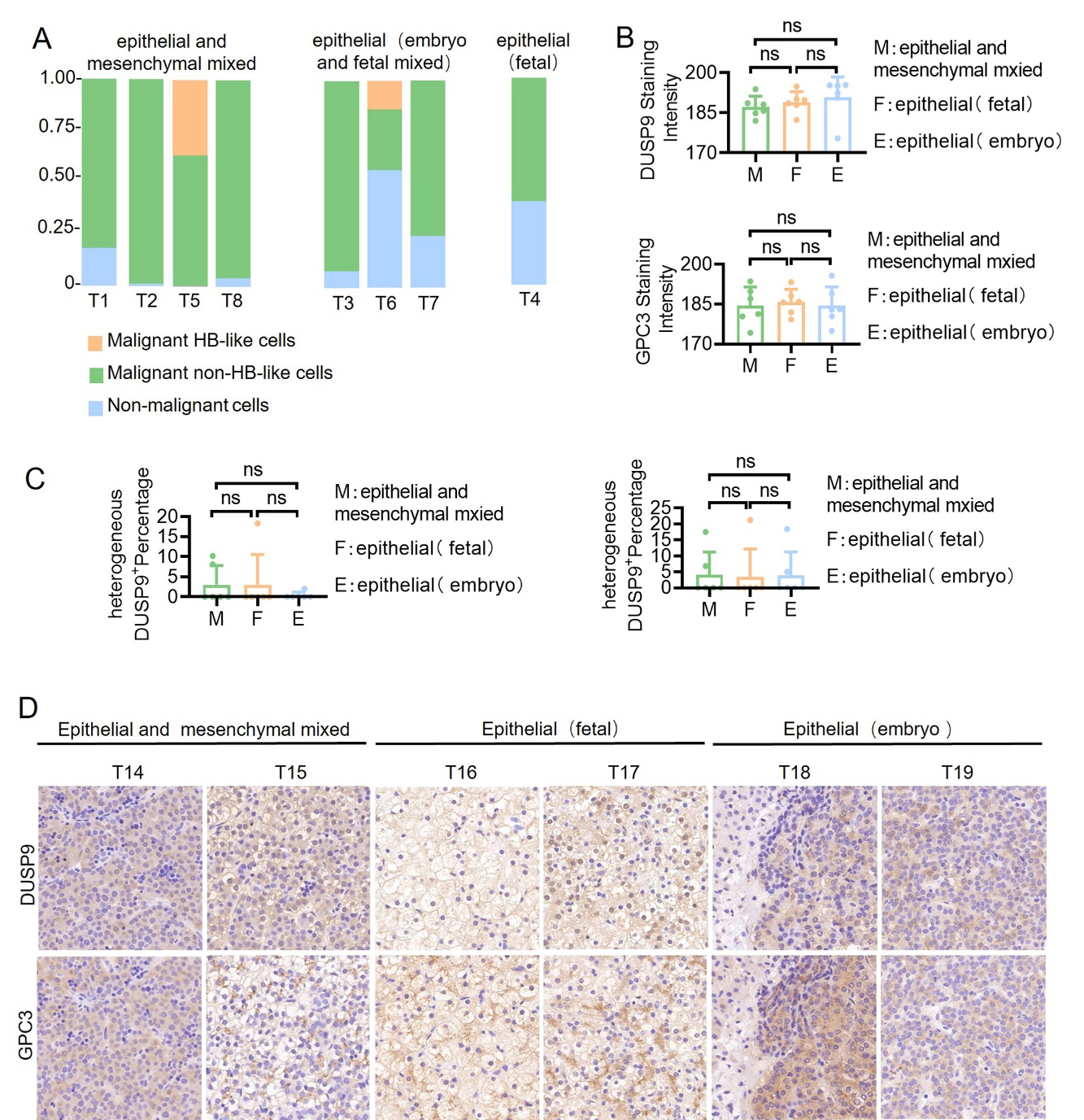


**Figure S4. Correlation analysis between the malignant HB-like cell subpopulation and histological subtypes.**

A. The proportions of the three hepatocyte cell types in tumorous tissues for scRNA-seq are shown as bar plots. B. Staining intensity of DUSP9 and GPC3 in tissue sections with different histological subtypes. C. The proportion of heterogeneous DUSP9/GPC3-positive cells in different histological hepatoblastoma tissues. D. Representative IHC staining images of DUSP9 and GPC3 in tissue sections with different histological subtypes.


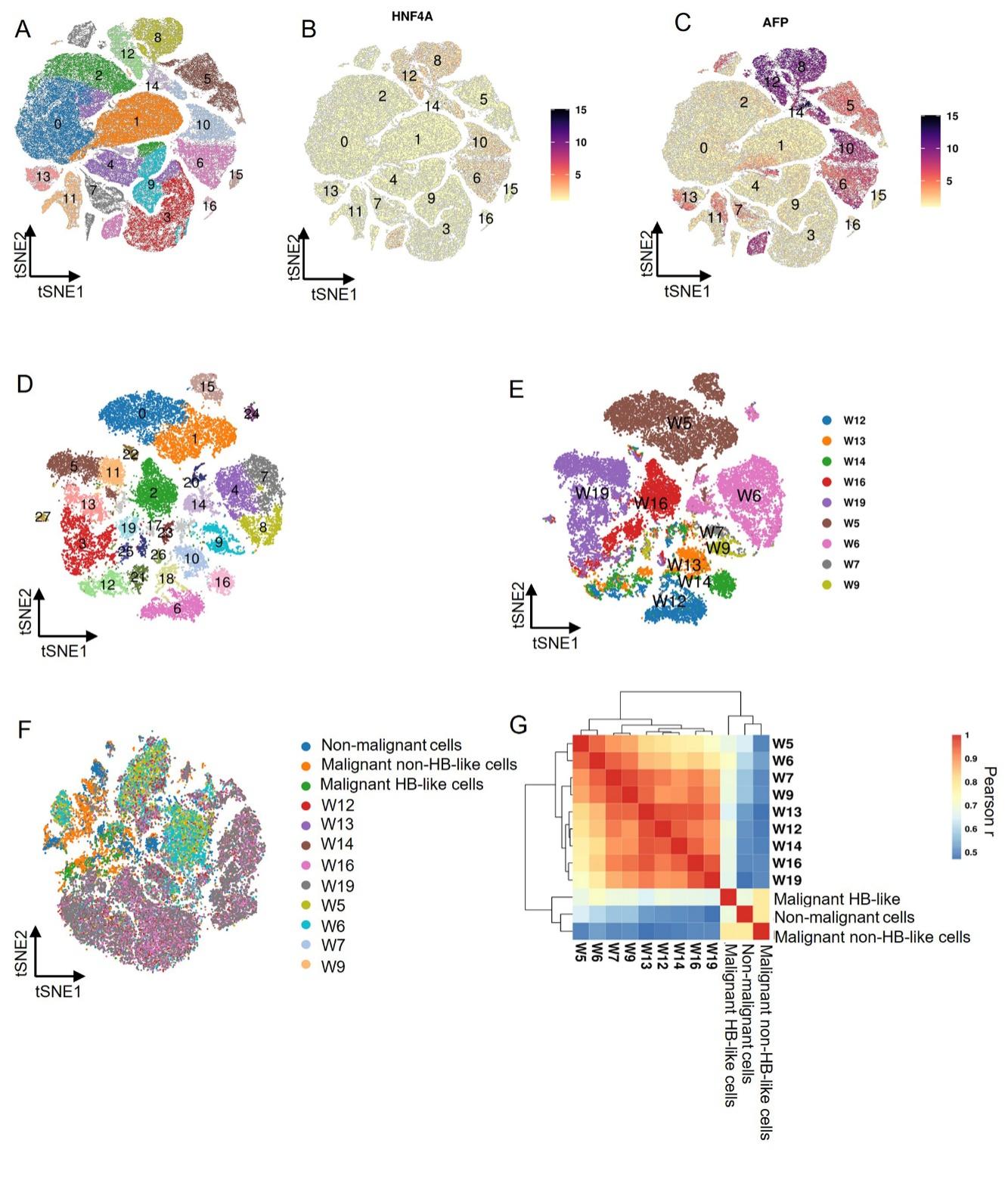


**Figure S5. Hepatocyte identification in fetal livers in the published scRNA-seq CRA002443 dataset.**

1. Clustering of cells from CRA002443 showing 13 clusters in the plot. Each cluster is shown in different colors. B-C. Expression levels of hepatocyte marker genes HNF4A and AFP in CRA002443. D. Clustering of hepatocytes in CRA002443, showing 26 clusters in the plot. Each cluster is shown in a different color. E. Clustering of hepatocytes from CRA002443, annotated by time points; w, week. Each time point is shown in a different color. F. TSNE plot showing cell types by integrating fetal and infant liver single-cell RNA seq datasets. G. Pearson’s correlation analysis shows the correlation between CRA002443 hepatocytes and the three hepatocyte subpopulations.


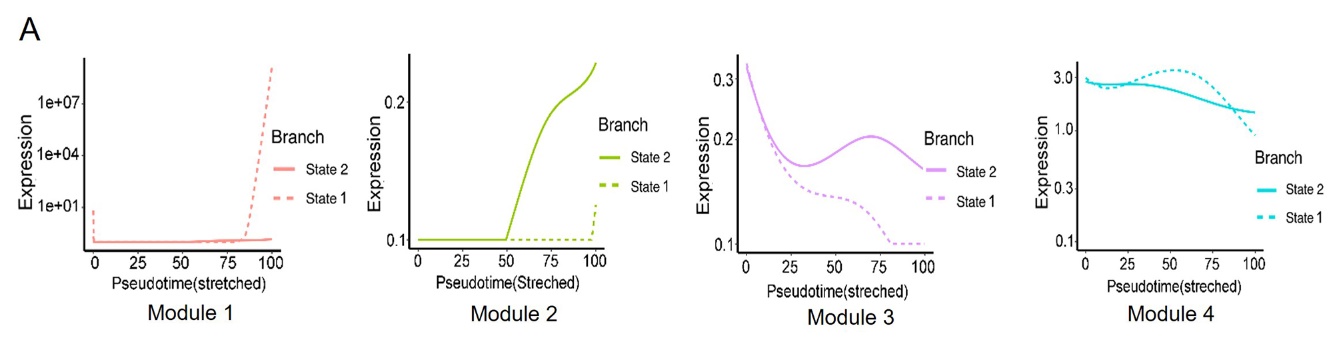


**Figure S6. Analysis of hepatocyte cell transition states in hepatoblastomas.**

1. Two-dimensional plots showing the different gene expression modules during transition along the pseudotime trajectory.


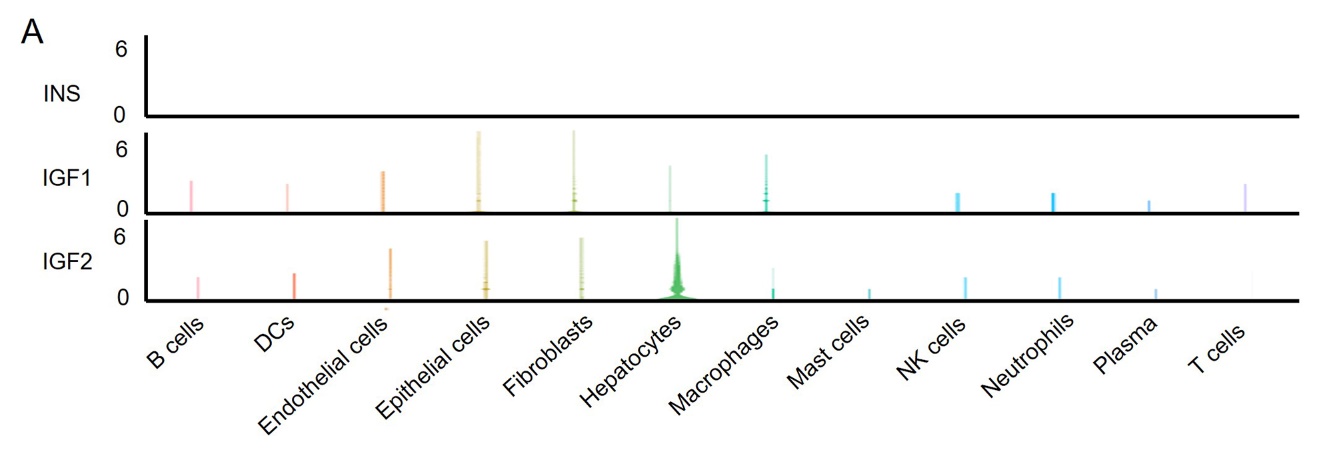


**Figure S7. Expression levels of Insulin, IGF1 and IGF2 in different cell types**

1. Violin plotting showed the expression levels of Insulin, IGF1 and IGF2 in the indicated cell types.


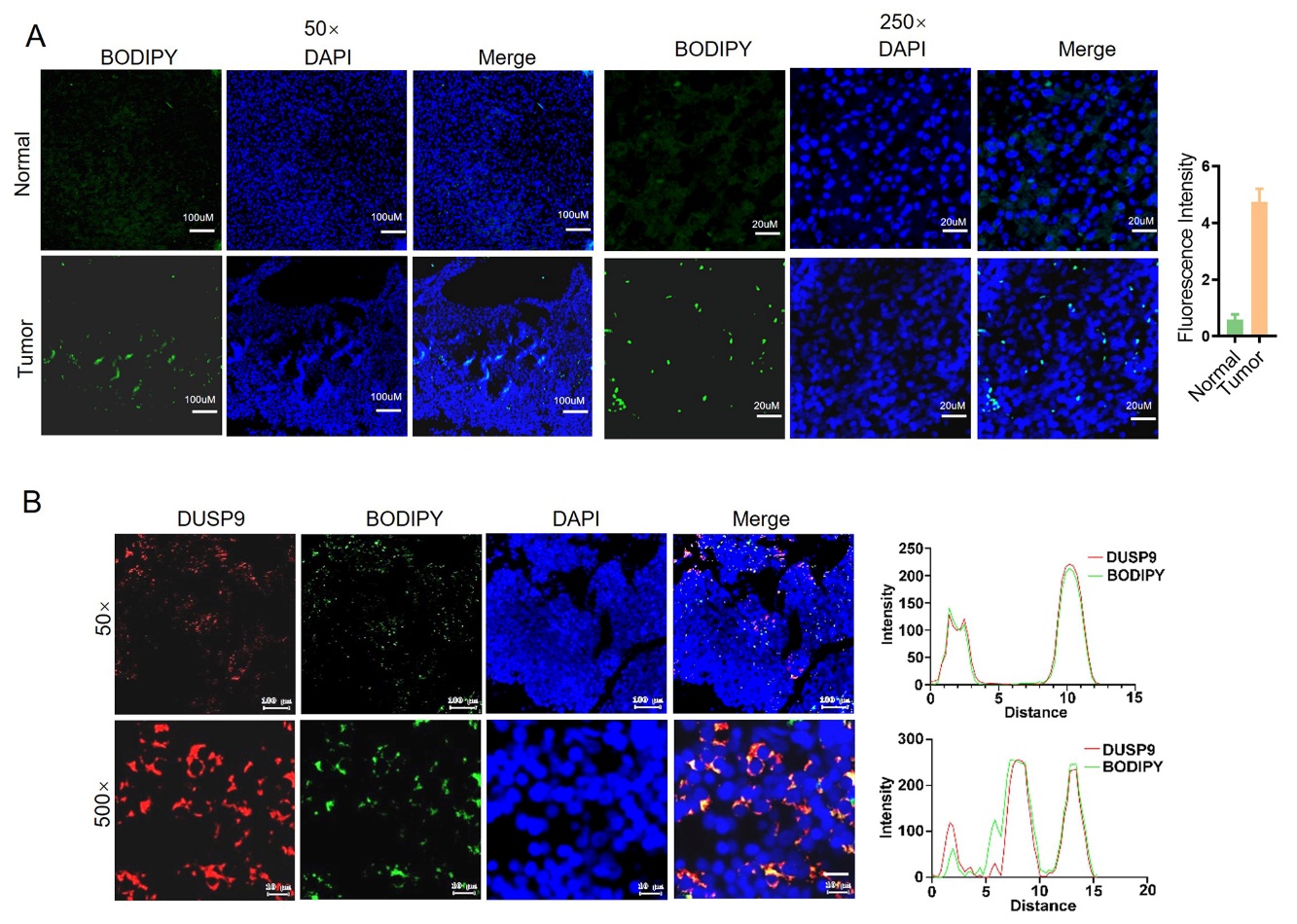


**Figure S8. Flurescences staining of primary hepatoblastoma tumors and matched paracancerous liver tissues.**

1. LDs were stained with BODIPY 493/503 in primary hepatoblastoma tumors and paired paracancerous liver tissues. B. BODIPY 493/503 and DUSP9 antibody were co-stained in hepatoblastoma tumors.


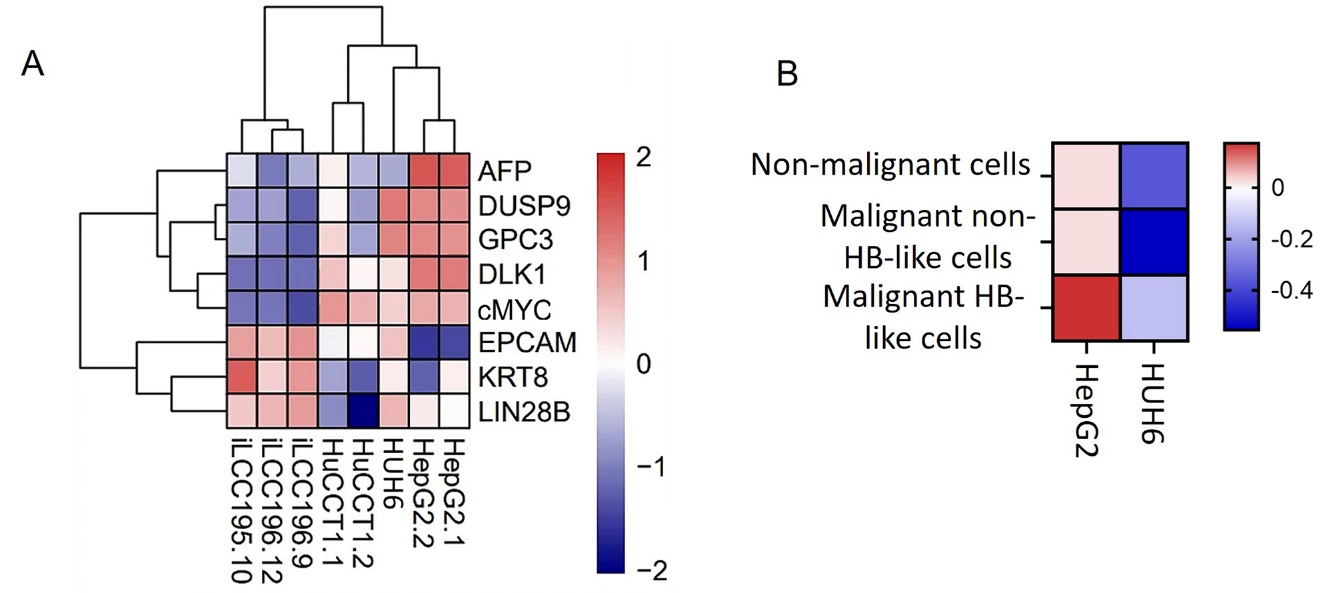


**Figure S9. Similarity analysis of hepatoblastoma tumor cell subpopulations and hepatoblastoma cell lines.**

A. Pearson’s correlation analysis of cell lines (x axis) based on eight malignant HB-like cells signatures in GSE168997. B. Similarity analysis of hepatoblastoma hepatocyte subpopulations (y axis) to cell lines (x axis), using gene set variation analysis (GSVA).


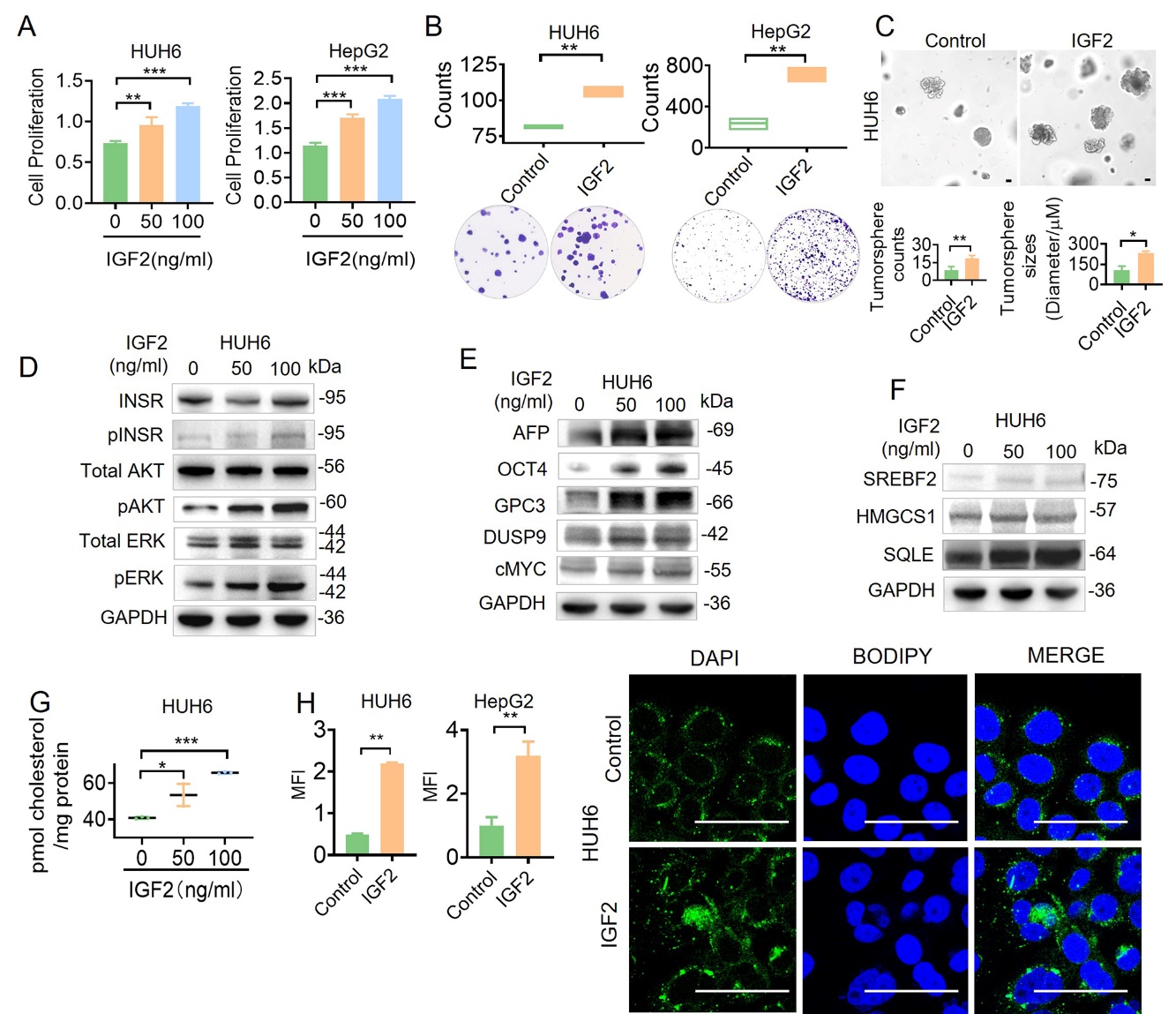


**Figure S10. IGF2 maitains cholesterol accumulation and stemness in hepatoblastoma cells.**

A-B. Effects of IGF2 on proliferation and viability as shown by CCK8 assay (A) and colony formation assays (B) at the indicated concentrations. Shown are means ± SD (n = 3). C. Tumorsphere formation assays of HUH6 cells with IGF2 are shown. Scale bar, 50mm. Shown are means ± SD (n = 3). D. The expression level of IGF2 activated signaling pathway related proteins were analyzed in HUH6 cells with IGF2 treatment by western blot. E. The expression level of HB stemness related proteins were analyzed in HUH6 cells with IGF2 treatment by western blot. F. The expression level of cholesterol metabolic pathway related proteins were analyzed in HUH6 cells with IGF2 treatment by western blot. G. The cholesterol concentrations were tested in HepG2 cells with IGF2 treatment for 5 days. H. Lipid droplets (LDs) were stained with BODIPY 493/503 in HUH6 and HepG2 cells with IGF2 treatment for 5 days. Scale bar, 50mm.


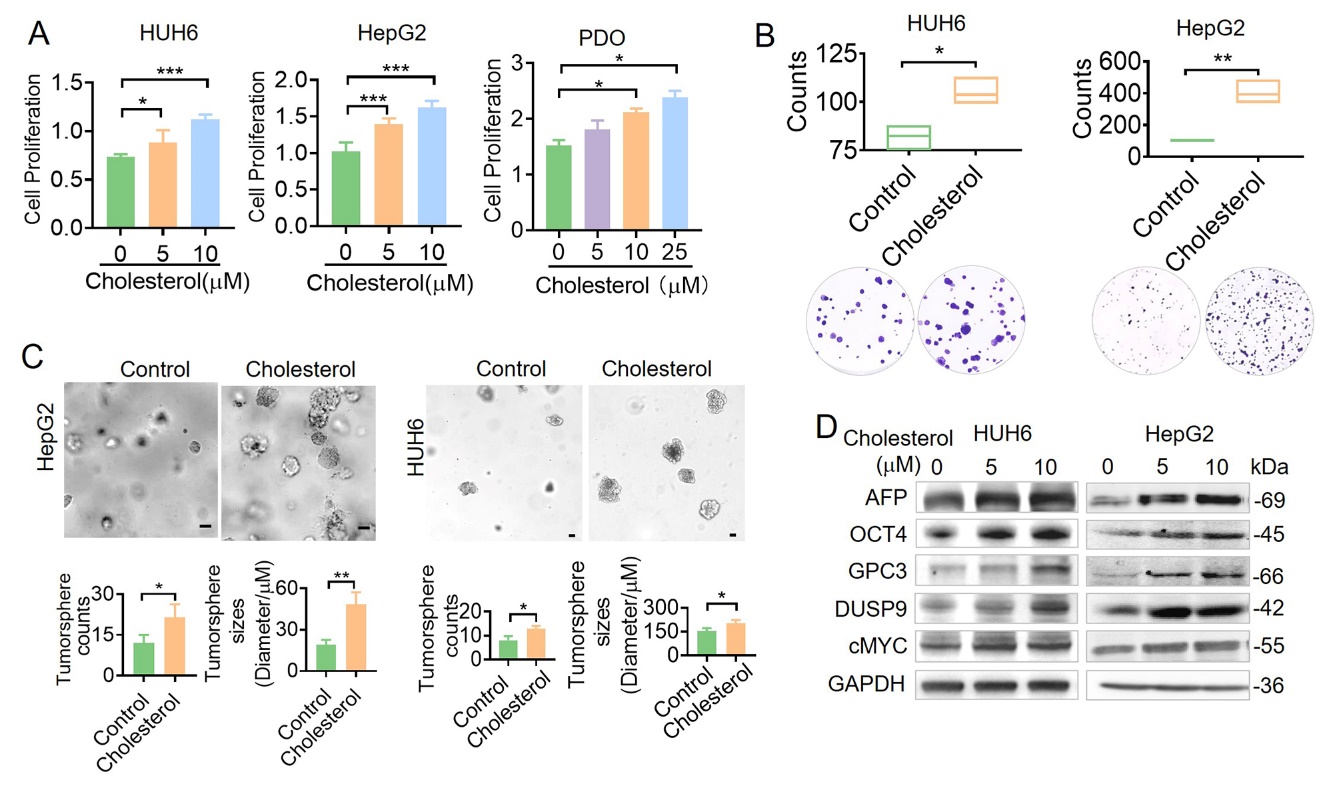


**Figure S11. Cholesterol sustains stemness of hepatoblastoma cells.**

A-B. Effects of cholestrol on proliferation and viability as shown by CCK8 assay (A) and colony formation assays (B) at the indicated concentrations. Shown are means ± SD (n = 3). C. Representative images of HUH6 and HepG2 cells treated with cholesterol on day 5 are shown. Scale bar, 50mm. D. The expression levels of hepatoblast stemness related proteins were analyzed in HUH6 and HepG2 cells with or without cholesterol treatment by western blot.


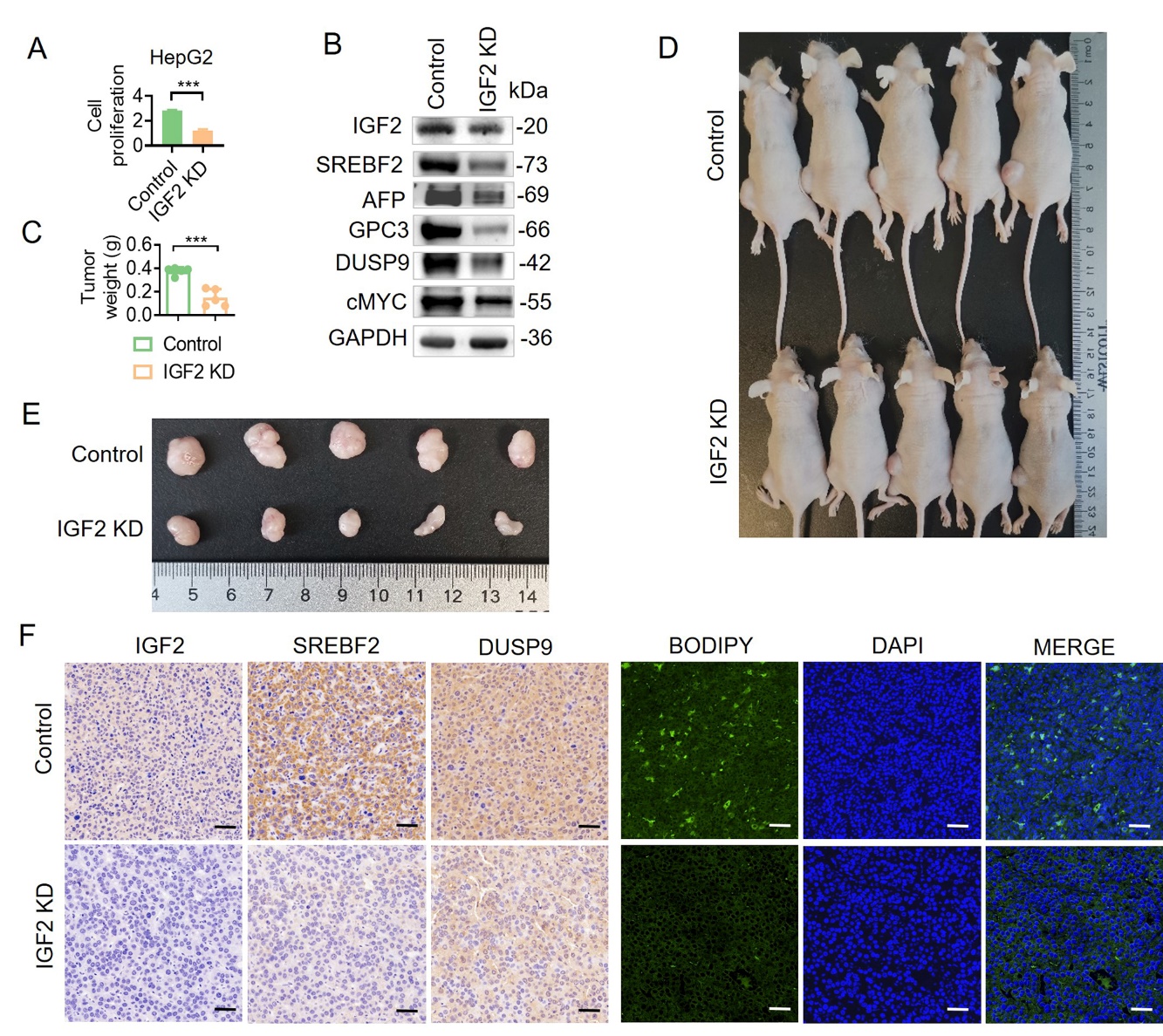


**Figure S12. Down-regulation of IGF2 diminishes hepatoblastoma cell stemness and reduce tumor growth in vivo.**

1. The HepG2-IGF2 KD cell proliferation ability was shown by CCK8 assay. B. The expression level of SREBF2 and HB stemness related proteins were analyzed in HepG2-IGF2 KD cells by western blot. C-E. The represent image and weights of tumors from mice. F. The represent IHC image of IGF2, SREBF2, DUSP9 and LDs in the mice tumors. Scale bar, 50 μm.


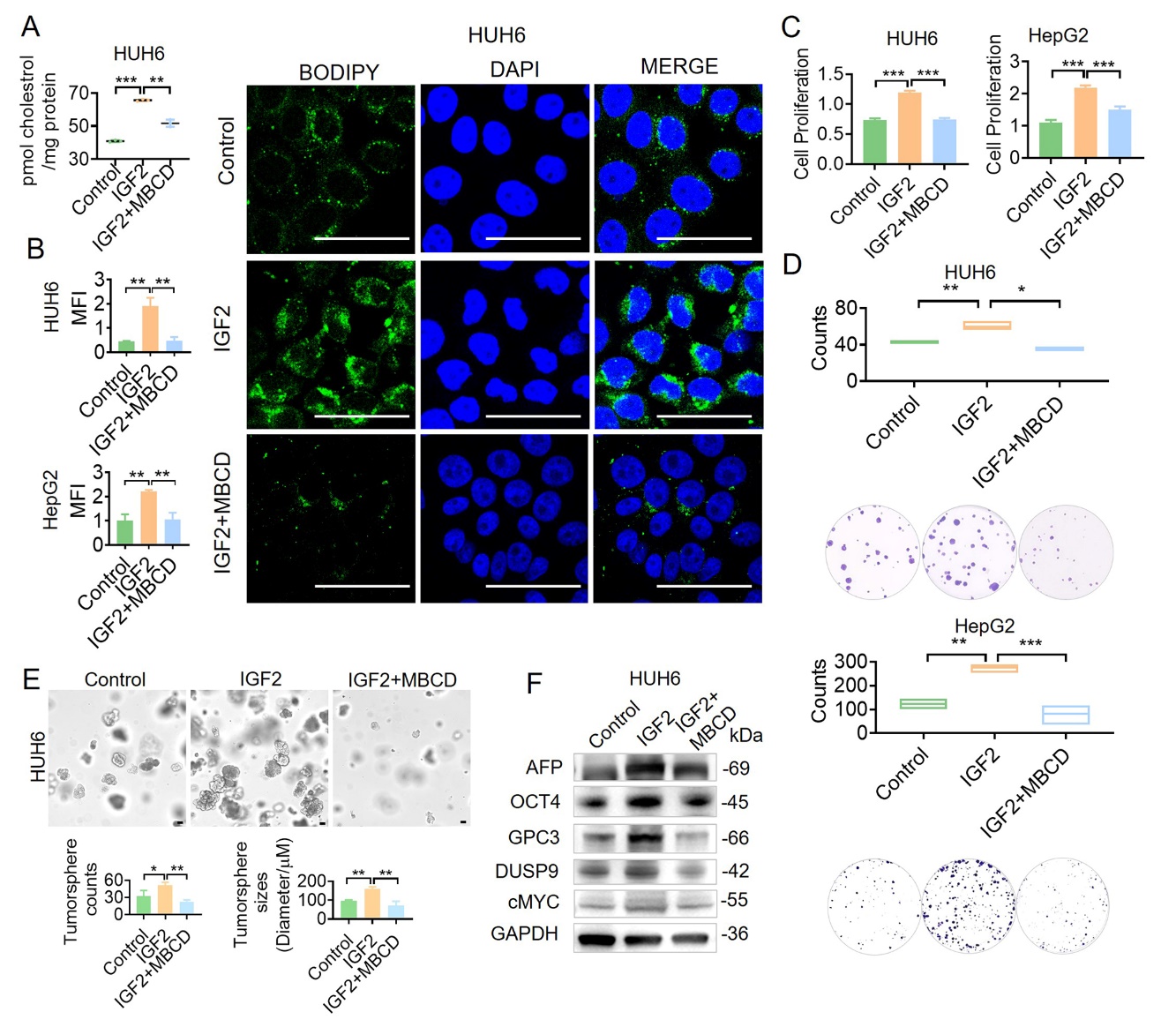


**Figure S13. IGF2 sustains stemness via abnormal accumulated cholesterol in hepatoblastoma.**

A. The cholesterol concentrations were tested in HUH6 cells with IGF2 treatment for 5 days. B. LDs were stained with BODIPY 493/503 in HUH6 and HepG2 cells with IGF2 (100mM) and MBCD (1mM) treatment. Scale bar, 50mm. C-D. Effects of IGF2 (100mM) and MBCD (1mM) on proliferation and viability as shown by CCK8 assay (C) and colony formation assays (D). Shown are means ± SD (n = 3). E. Tumorsphere formation assay comparing impacts of IGF2 (100mM) and MBCD (1mM) treatments of HUH6 cells. Shown are means ± SD (n = 3). Scale bar, 50mm. F. The expression of HB stemness related proteins were analyzed in HUH6 cells with IGF2 (100mM) and MBCD (1mM) treatment by western blot.


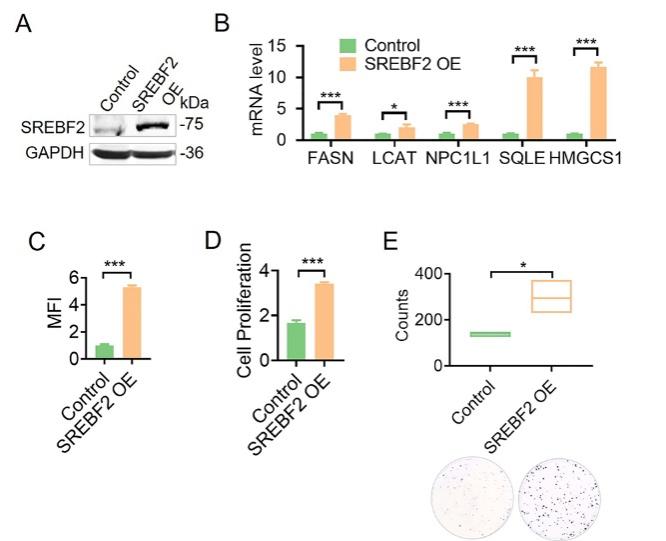


**Figure S14. SREBF2 over expression mediates the abnormal accumulated cholesterol and enhances stemness.**

A. SREBF2 overexpression in HepG2 cells was verified by western blot. B. RT-PCR analysis of SREBF2 target genes related with cholesterol metabolism in IGF2 (100mm) and SREBF2 siRNA treated HepG2 cells. C. The bars show MFI of LDs in HepG2 cells with SREBF2 overexpression. D-E. Effects of SREBF2 on proliferation and viability of HepG2 cells as shown by CCK8 assays (D) and colony formation assays (E). Shown are means ± SD (n = 3).


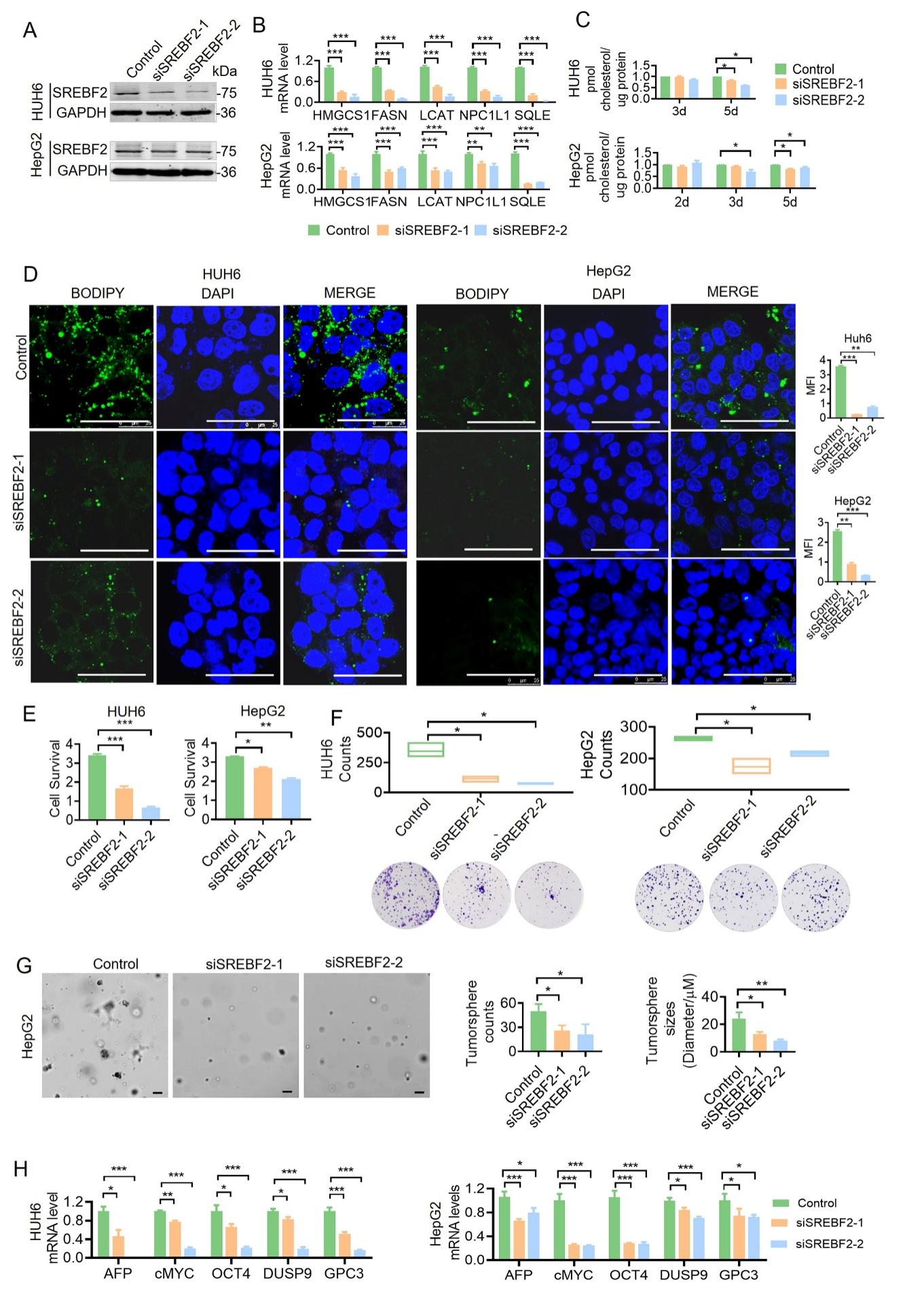


**Figure S15. SREBF2 knockdown abolished the abnormal accumulated cholesterol and stemness in hepatoblastoma cells.**

1. SREBF2 silencing efficiency using siRNAs in HUH6 and HepG2 cells was verified by western blot. B. RT-PCR analysis of SREBF2 target genes related with cholesterol metabolism in siRNAs transfected HUH6 and HepG2 cells. C. The cholesterol concentrations were tested in HUH6 and HepG2 cells with knockdown SREBF2. D. LDs were stained with BODIPY 493/503 in HUH6 and HepG2 cells with SREBF2 knockdown. Scale bar, 50mm. E-F. Effects of knockdown SREBF2 on proliferation and viability of HUH6 and HepG2 cells as shown by CCK8 assays (E) and colony formation assays (F). Shown are means ± SD (n = 3). G. Tumorsphere formation assay comparing impacts of knockdown SREBF2 on HepG2 cells. Shown are means ± SD (n = 3). H. The expression levels of HB stemness related genes were analyzed in HUH6 and HepG2 cells with knockdown SREBF2 by RT-PCR. * indicated p < 0.05, ** indicated p < 0.01, *** indicated p < 0.001.


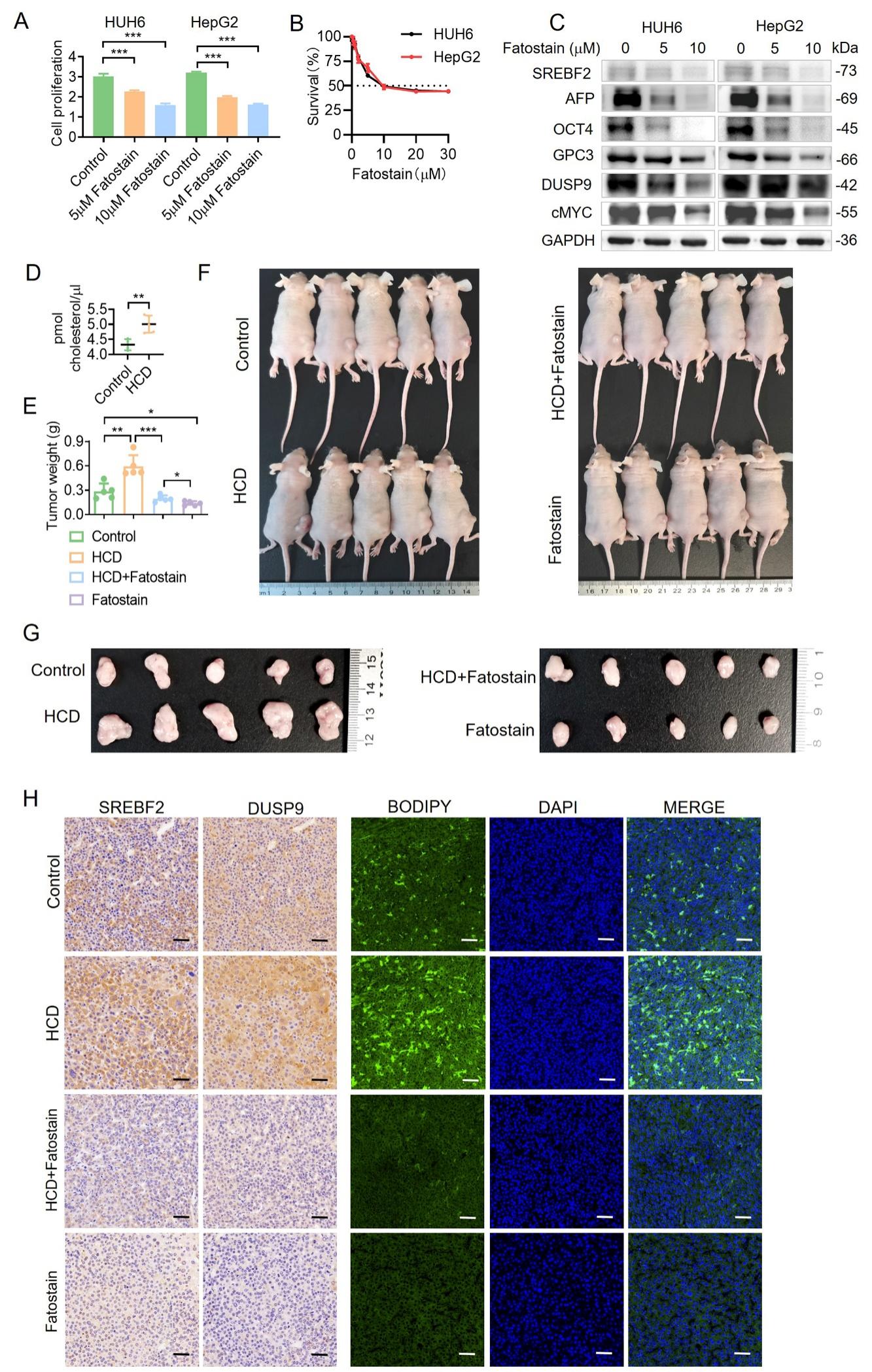


**Figure S16. Fatostain, an SREBF2 inhibitor, diminishes hepatoblastoma stemness and tumor growth in vitro and in vivo.**

1. The cell proliferation abilitys with Fatostain treatment for 72hs were shown by CCK8 assay. B. The IC50s of Fatostain for 72hs were shown. C. The expression level of SREBF2 and HB stemness related proteins were analyzed in HUH6 and HepG2 cells by western blot. D. Blood samples were collected from both HCD (high-cholesterol diet)-fed mice and control mice, and the serum cholesterol concentrations were measured. E-G. The represent images and weights of tumors from mice. H. The represent IHC image of SREBF2, DUSP9, and LDs in the mice tumors. Scale bar, 50 μm.


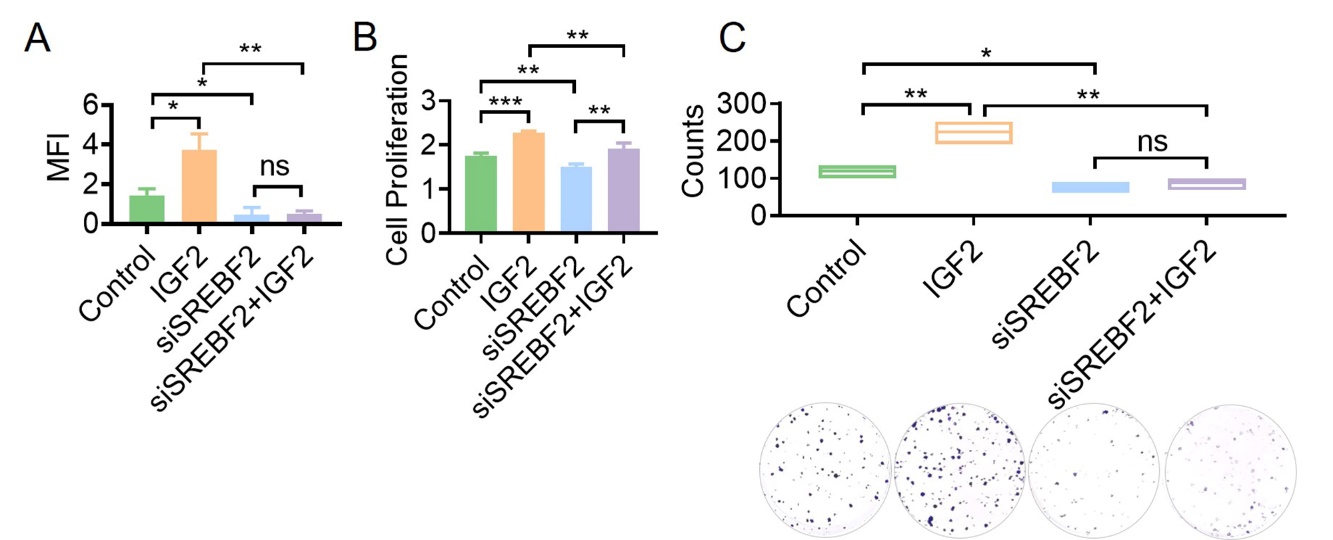


**Figure S17. SREBF2 mediate the abnormal accumulated cholesterol in activated IGF2 signaling pathway.**

A. The bars show MFI of LDs in HepG2 cells with IGF2 (100mm) and SREBF2 siRNA treatment. B-C. Effects of IGF2 (100mm) and SREBF2 siRNA treatment on proliferation and viability of HepG2 cells as shown by CCK8 assays (B) and colony formation assays (C).


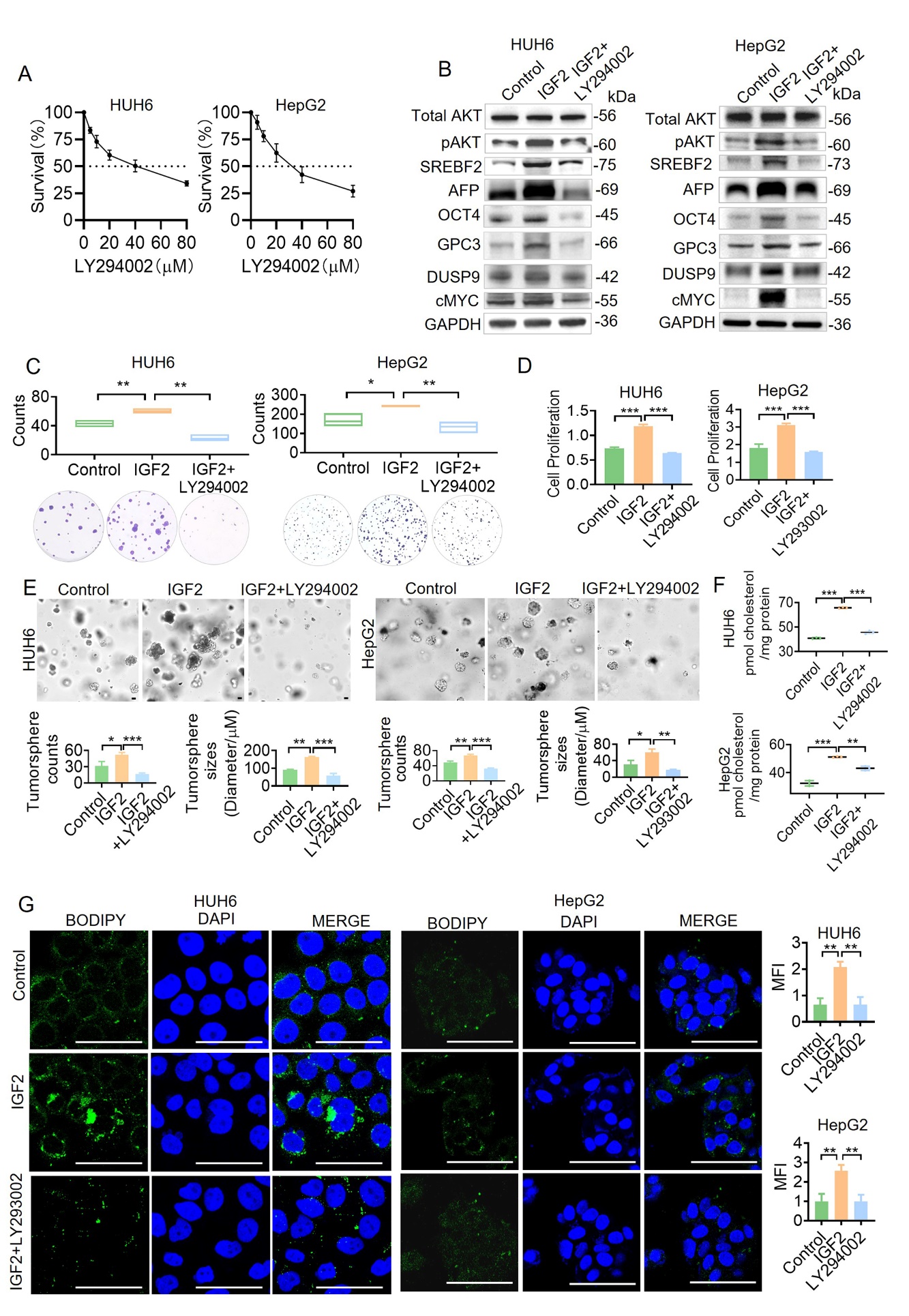


**Figure S18. IGF2 regulate SREBF2 through via pAKT signaling pathway in hepatoblastoma cells.** A. HUH6 and HepG2 cells survival rate in different concentrations of LY294002 for 48hs. B. The expression levels of AKT, SREBF2 and HB stemness related proteins were analyzed by western blot in HUH6 and HepG2 cells with IGF2 (100mM, 5ds) and LY294002 (20mM, 48hs) treatment for 5ds. C-D. The proliferation and viability of HUH6 and HepG2 cells were shown by colony formation assays (C) and CCK8 assay (D) at the indicated treatments. Shown are means ± SD (n = 3). E. Tumorsphere formation assays of HUH6 and HepG2 cells with the indicated treatments are shown (IGF2, 100mM, 5ds, and LY294002, 20mM, 48hs). Scale bar, 50mm. F. The cholesterol concentrations were tested in HUH6 and HepG2 cells with the indicated treatment (IGF2, 100mM, 5ds, and LY294002, 20mM, 48hs). G. LDs were stained with BODIPY 493/503 in HUH6 and HepG2 cells with IGF2 (100mM, 5ds) and LY294002 (20mM, 48hs) treatment. Scale bar, 50mm.

**
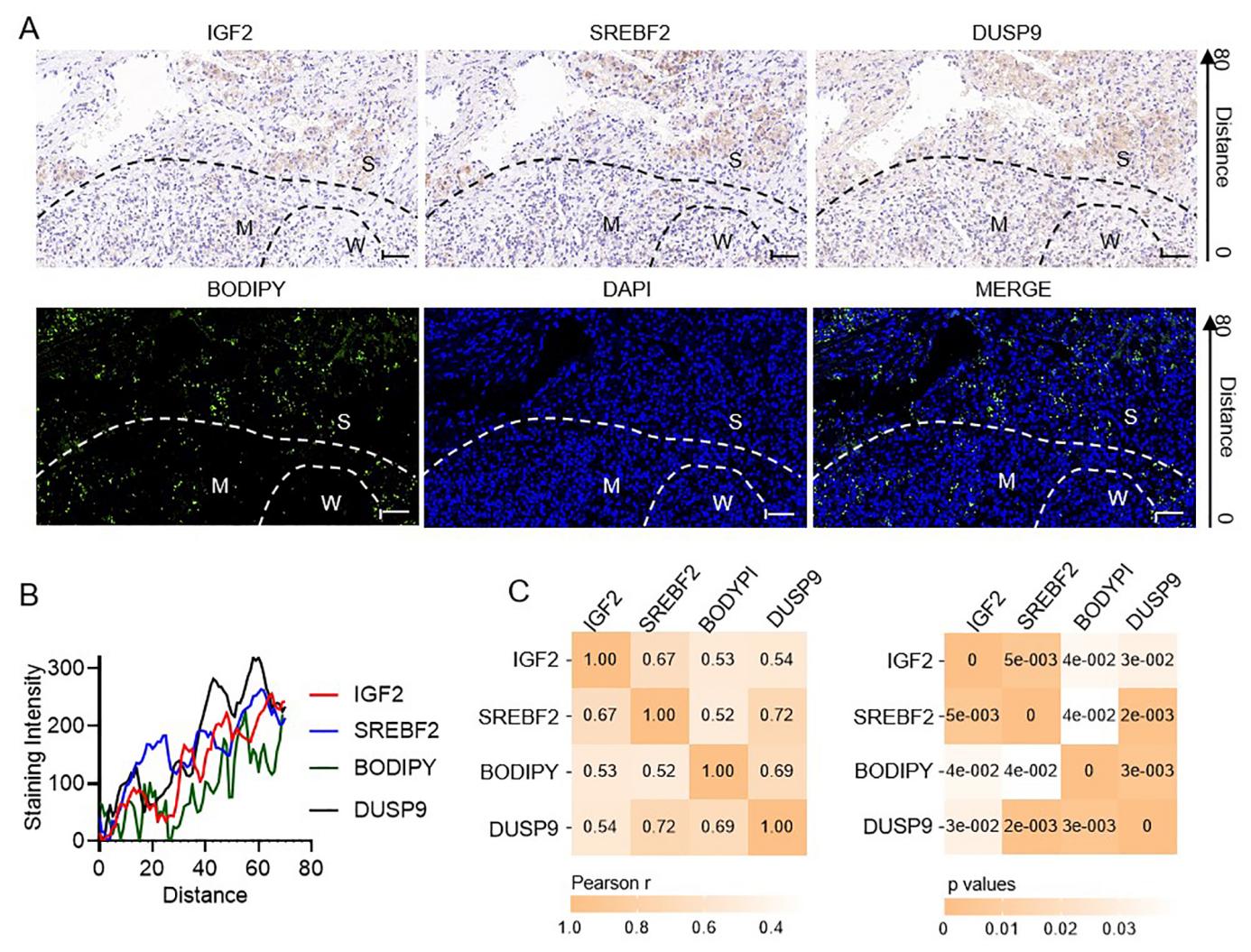
**

**Figure S19. Abnormal accumulated cholesterol is a relatively significant signature in malignant HB-like cells with activated IGF2 signaling pathway.**

A. The representative tissue images of IGF2, SREBF2, DUSP9, and LDs staining. The areas are classified into strong positive (Strong-S), moderate positive (Moderate-M), and weak positive (Weak-W) based on the intensity of the positive signals. B. The colocalization analysis of IGF2, SREBF2, DUSP9, and LDs in primary hepatoblastoma tumors by Image J. C. Pearson’s correlation analysis for IGF2, SREBF2, DUSP9, and LDs across the 16 hepatoblastoma patients’ tissues.


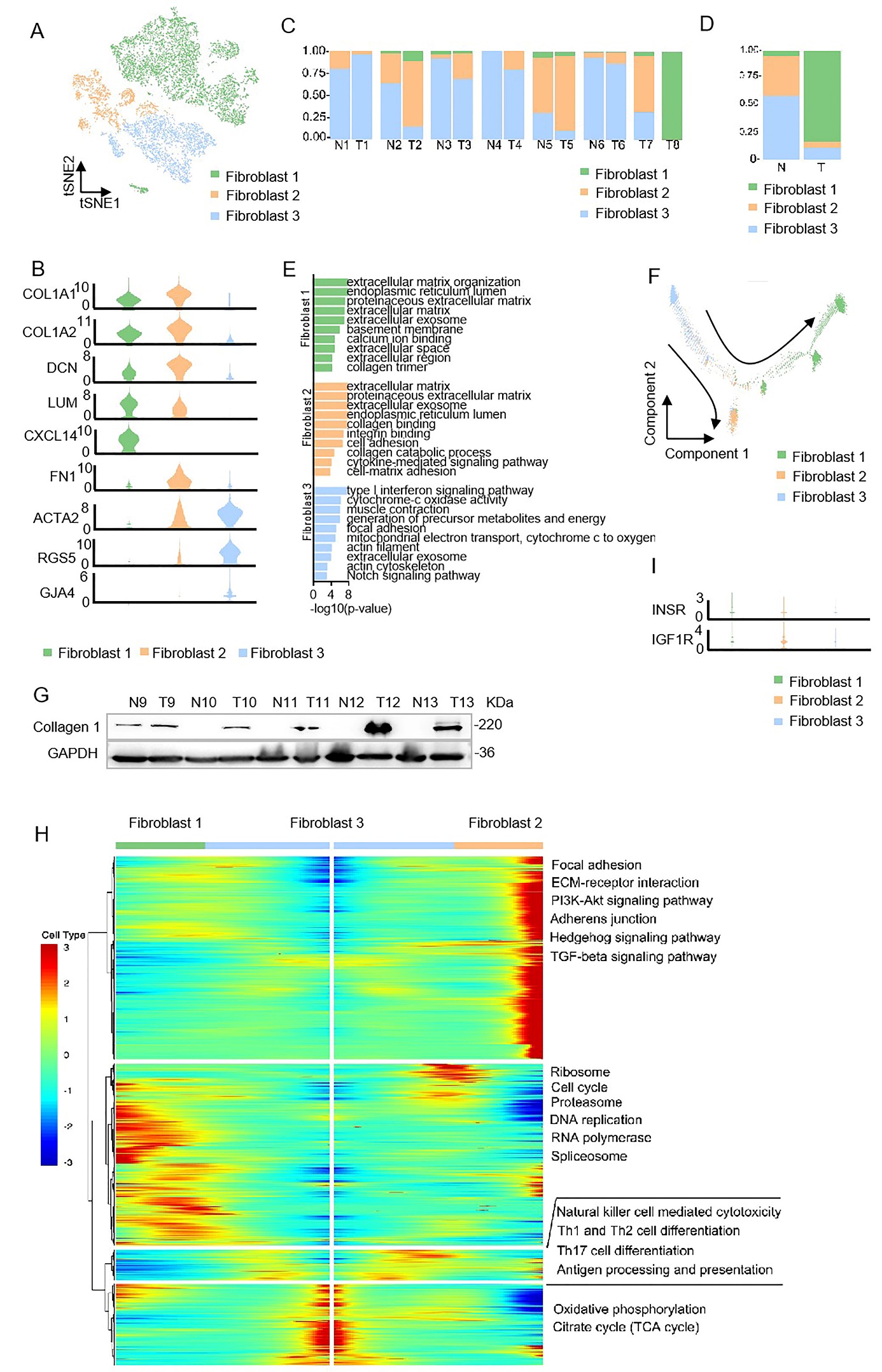
**Figure S20. Three different fibroblast subtypes are identified in hepatoblastoma.**

A. TSNE plotting showed of 10, 934 fibroblast cells from hepatoblastoma tissues revealing 3 fibroblast subtypes. B. Violin plotting showed the expression levels of classification marker genes in the indicated fibroblast subtypes. C. Proportion of 3 fibroblast subtypes showing in bar plots in different donors are shown. D. Proportion of 3 fibroblast subtypes showing in bar plot in hepatoblastoma tissues are shown. E. Marker genes enrichment analysis of three fibroblast subpopulations from hepatoblastoma tissues are shown. F. Developmental trajectory of fibroblast cells inferred by monocle2, colored by cell subtypes. G. The protein expression level of collagen 1 in 5 paired hepatoblastoma tissues. H. Representative signaling pathways of different states inferred by monocle2. Heatmap showing the dynamic changes in gene expression along the pseudotime in fibroblast 1 and fibroblast 2. I. Violin plotting showed the expression levels of INSR and IGF1R in the indicated fibroblast subtypes.


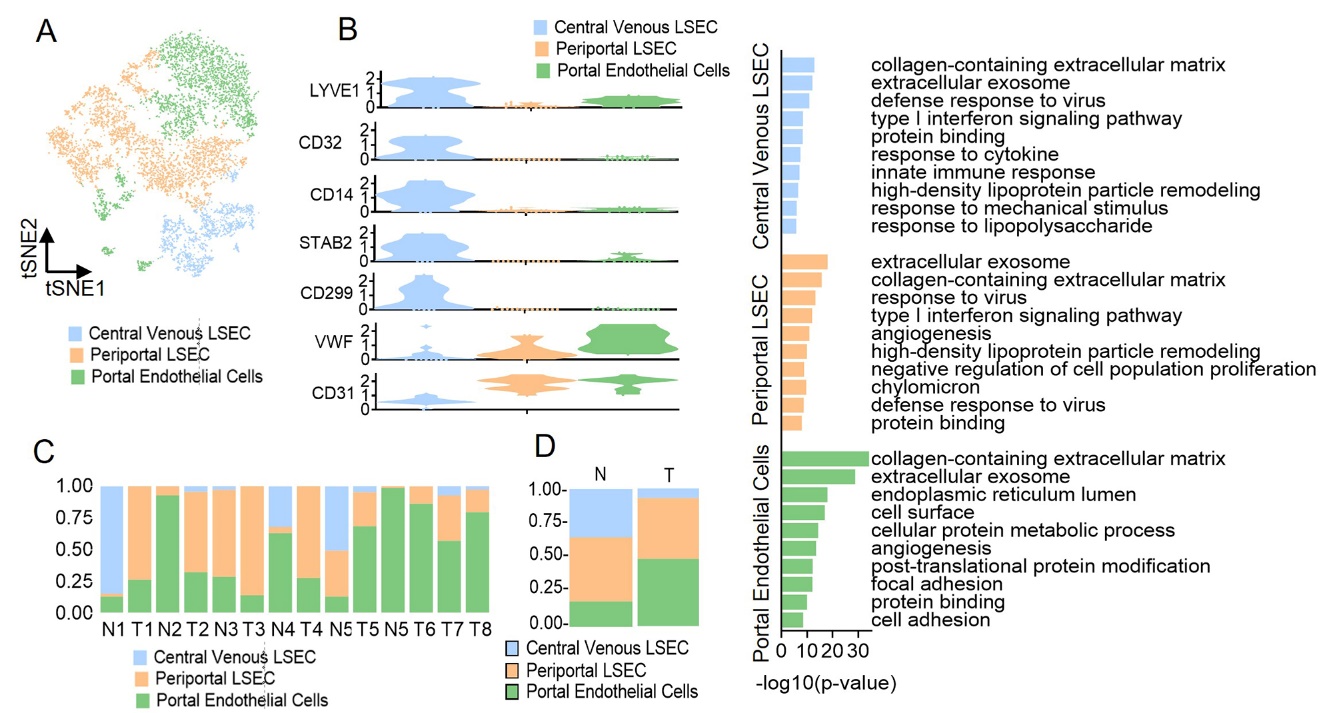


**Figure S21. Three different liver endothelial cells are identified in hepatoblastoma.**

1. TSNE plotting showed of 7, 757 endothelial cells from primary hepatoblastoma tumors and matched paracancerous liver tissues revealing 3 endothelial subtypes. B. Violin plotting showed the expression levels of classification marker genes in the indicated endothelial subtypes. C. Proportion of 3 endothelial subtypes are showed in bar plots in different donors. D. Proportion of 3 endothelial subtypes are showed in bar plot in hepatoblastoma tissues. E. Marker genes enrichment analysis of 3 endothelial subtypes from hepatoblastoma tissues.


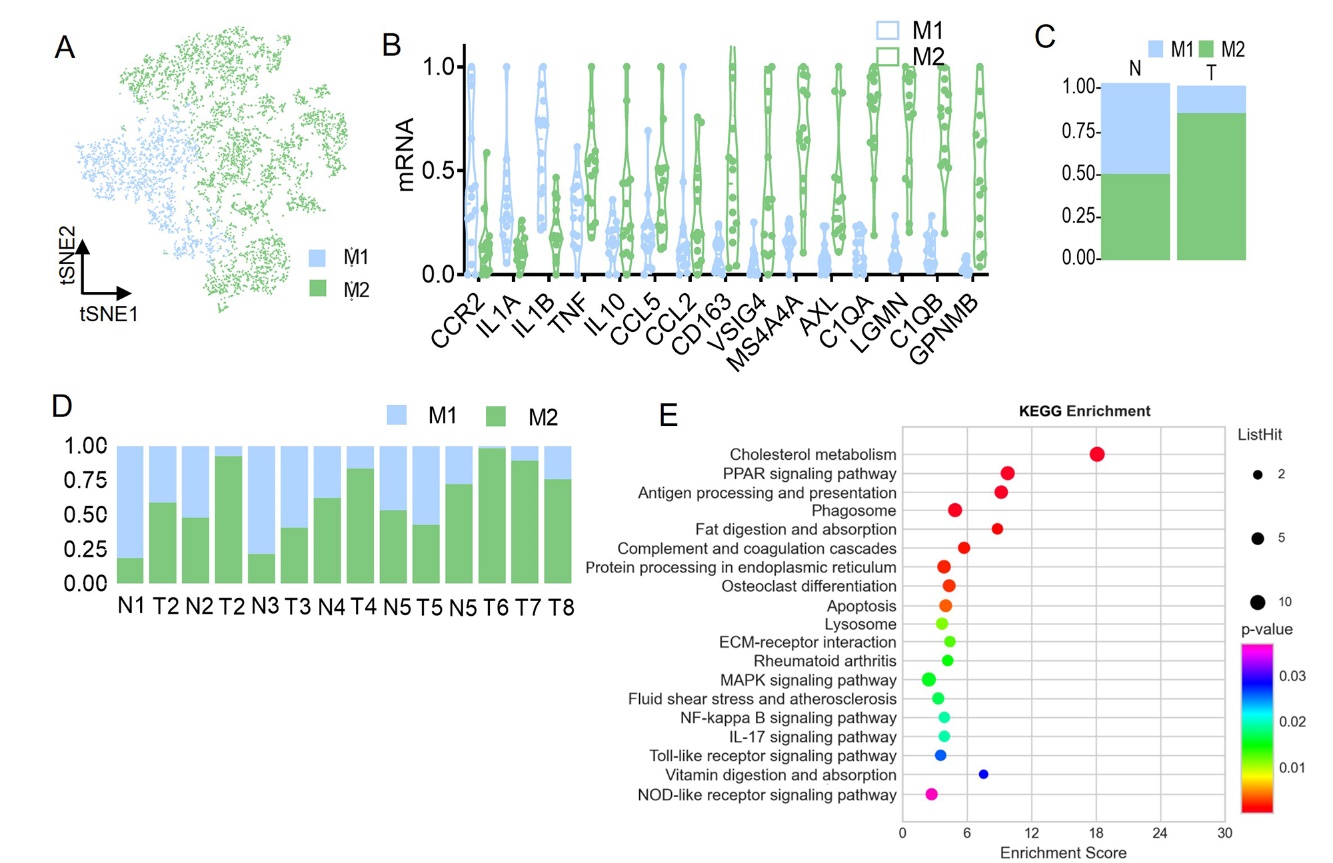


**Figure S22. The macrophages are in intermediate polarization states with pro-tumoral functions.**

1. TSNE plotting showed 6, 703 macrophages from hepatoblastoma tissues revealing 2 macrophage subtypes. B. Violin plotting showed the expression levels of marker genes for macrophage classification. C. Proportions of 2 macrophages subtypes showed in bar plots in different donors. D. Proportions of macrophages are showed in bar plot in tissues. E. Marker genes enrichment analysis of 2 macrophage subtypes from hepatoblastoma tissues.


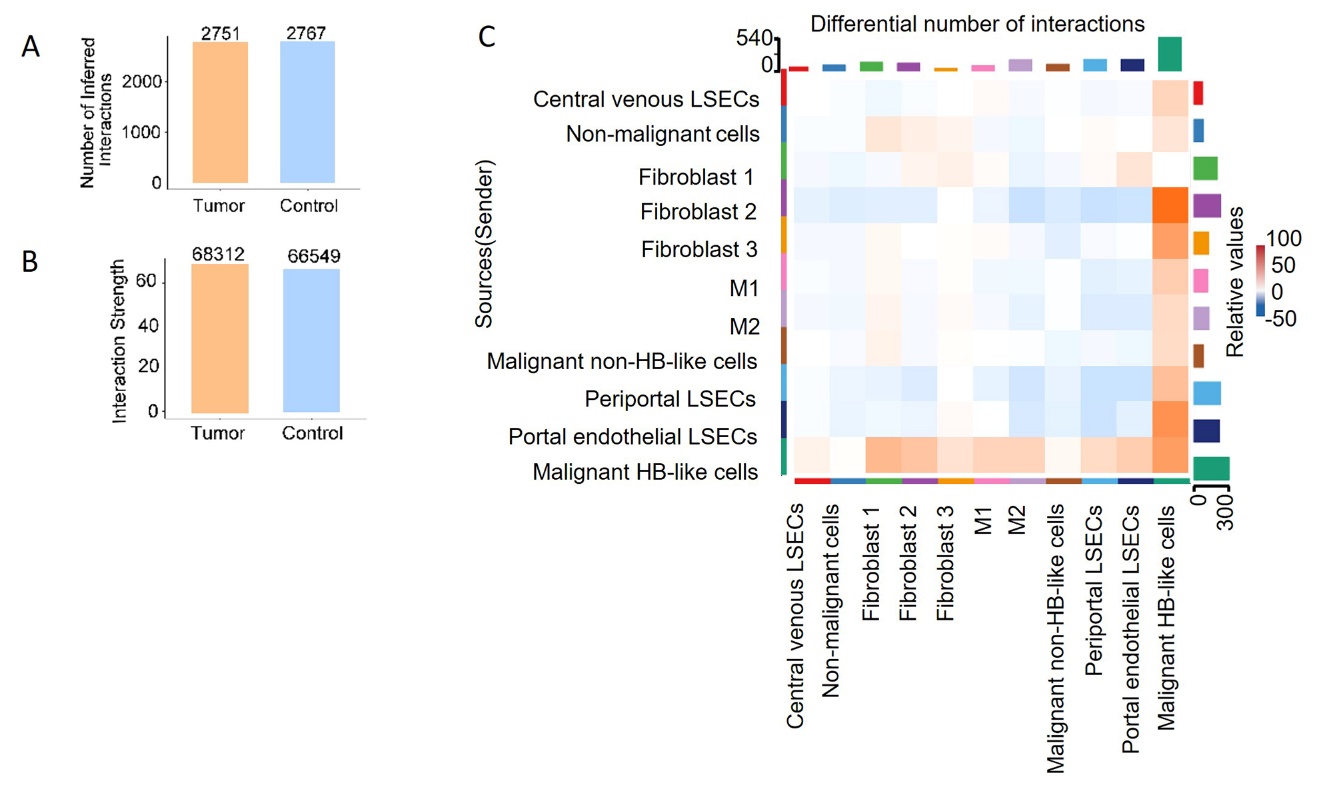


**Figure S23.** **Intercellular crosstalk in hepatoblastoma.**

A-B. Bar plotting showed the total number (A) and strength (B) of interactions between subcell types in hepatoblastoma tissues. C. Heatmap showed the number of interactions between subcell types in hepatoblastoma tissues. The color represents the communication probabilitys in hepatoblastoma tumors compared to these in paracancerous normal liver tissues.


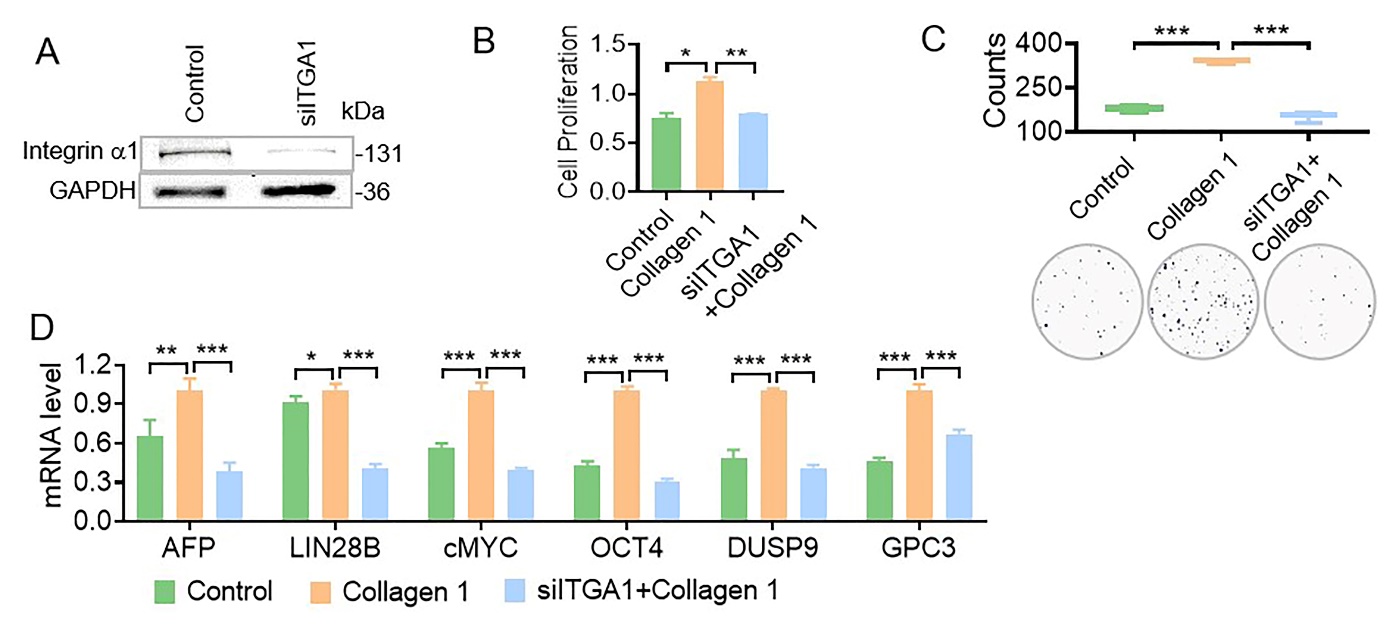
**Figure S24. Hepatoblastoma cells maintain malignancy through the collagen1/ integrin a1 axis.**A. Integrin a1 silencing efficiency using siRNAs in HepG2 cells was assessed by western blot. B-C. HepG2 cells with the indicated treatments were subjected to colony CCK8 assays (B) and tumorsphere formation assays (C) and to evaluate the cell proliferation and viability. Shown are means ± SD (n = 3). D. The expression levels of HB stemness related genes were analyzed in HepG2 cells with the indicated treatments by RT-PCR.


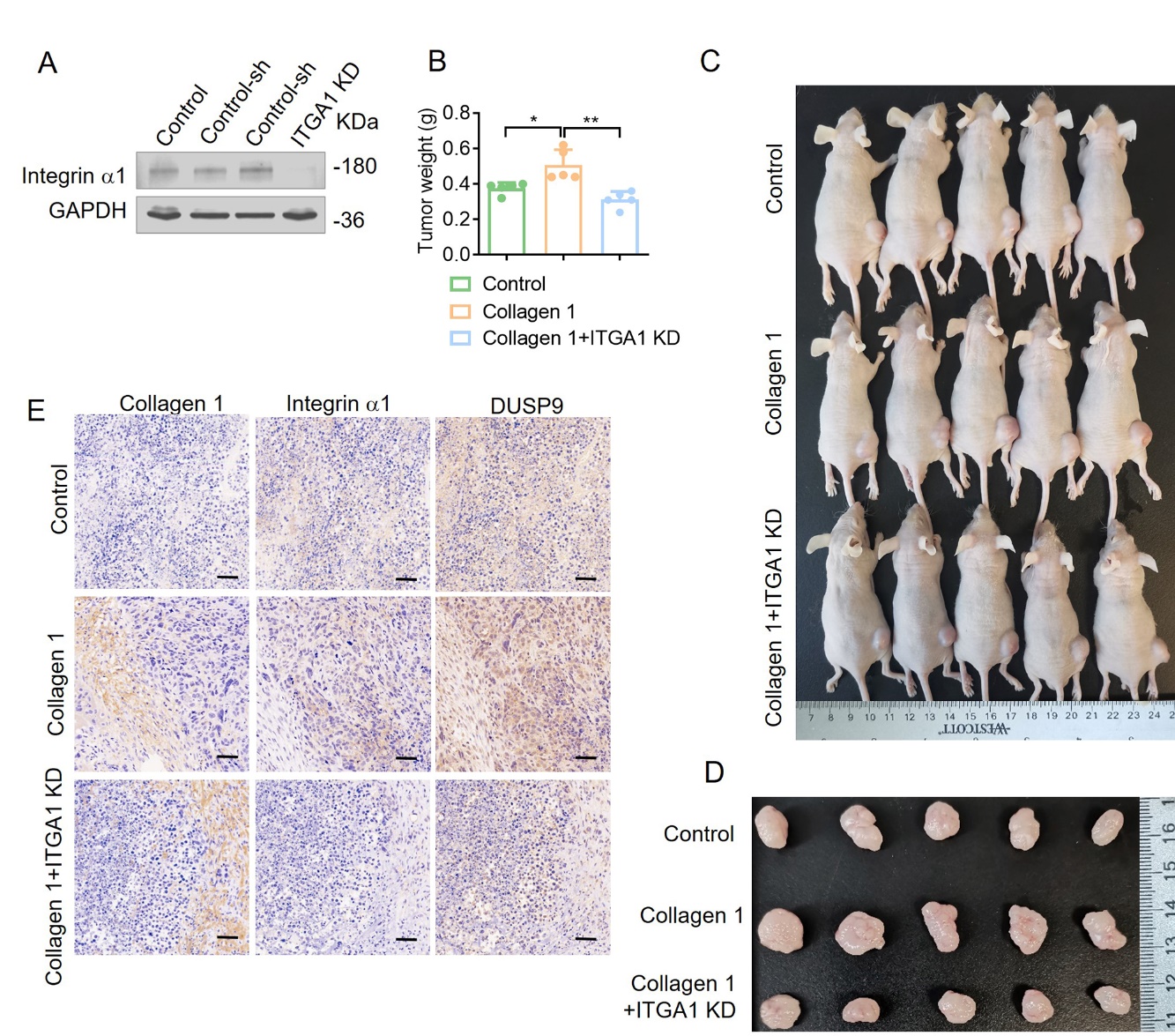


**Figure S25. Collagen1 promote tumor growth in vivo.**

A. The expression level of integrin a1 in HepG2-ITGA1 KD cells was assessed by western blot. B-D. The represent image and weights of tumors from mice. E. The represent IHC image of integrin a1, collagen 1 and DUSP9 in the mice tumors. Scale bar, 50 μm.

**Table S5. Statistical analysis of correlations between the proportions of heterogeneous DUSP9/ GPC3 positive cells and clinical course in hepatoblastoma tissues.**

|  | **Clinical Course** | | **Total (n=28, %)** | **p value** |
| --- | --- | --- | --- | --- |
|  | **Alive (n=18, %)** | **Dead (n=10, %)** |  |  |
| **DUSP9** |  | | | |
| heterogeneous positive | 4 (40.0) | 6 (60.0) | 10 (35.7) | 0.097 |
| non-heterogeneous positive | 14 (77.8) | 4 (22.2) | 18 (64.3) |  |
| **GPC3** |  | | | |
| heterogeneous positive | 5 (62.5) | 3 (37.5) | 8 （28.57） | 1.000 |
| non-heterogeneous positive | 13 (65.0) | 7 (35.0) | 20 （71.43） |  |
| **Total** | 18 (100%） | 10 (100%） | 28 （100%） |  |

**Table S15. Statistical analysis of correlations between clinicopathological characteristics and AFP expressions in serums from hepatoblastoma patients (Pearson c2 test).**

| **Hepatoblastoma (n=33)** | **AFP** | | **p value** |
| --- | --- | --- | --- |
|  | **Low（n=11）^(1)^** | **High（n=22）** |  |
| **Age** |  |  |  |
| ≤24 months | 6(54.5%) | 12(54.5%) | 1 |
| >24 months | 5(45.5%) | 10(45.5%) |  |
| **Gender** |  |  |  |
| Female | 4(21.1%) | 10(45.5%) | 0.719 |
| Male | 7(78.9%) | 12(54.4%) |  |
| **PRETEXT** |  |  |  |
| I-II | 7(63.6%) | 12(54.5%) | 0.719 |
| III-IV | 4(36.4%) | 10(45.4%) |  |
| **Metastasis** |  |  |  |
| Yes | 3(27.2%) | 7(31.8%) | 1 |
| No | 8(72.8%) | 15(68.2%) |  |
| **Histology** |  |  |  |
| Epithelial | 6(54.5) | 4(18.2%) | 0.134 |
| MIX | 4(21.1) | 14(63.6%) |  |
| NA | 1(24.4%) | 4(18.2%) |  |
| **Total** | 11(100%) | 22(100%) |  |

(1).AFP levels: high (≥2000 ng/ml) and low (<2000 ng/ml) groups

**Table S16. Primer sequences used in this study.**

| **Gene name** |  | **Primer sequences** |
| --- | --- | --- |
|  |  |  |
| 18s | Forward | 5’-AGCCACCCGAGATTGAGCA-3’ |
|  | Reverse | 5’-TAGTAGCGACGGGCGGTGTG-3’ |
|  |  |  |
| SREBF2 | Forward | 5’-GGCAGTGGTGGTAGTGGTAGC-3’ |
|  | Reverse | 5’-GCCGAGGGAGAGAAGGAAGG-3’ |
|  |  |  |
| SQLE | Forward | 5’- GGCATTGCCACTTTCACCTAT-3’ |
|  | Reverse | 5’- GGCCTGAGAGAATATCCGAGAAG-3’ |
|  |  |  |
| FASN | Forward | 5’- AAGGACCTGTCTAGGTTTGATGC-3’ |
|  | Reverse | 5’- TGGCTTCATAGGTGACTTCCA-3’ |
|  |  |  |
| LACT | Forward | 5’- ACCTGGTCAACAATGGCTACG-3’ |
|  | Reverse | 5’- TAGAGCAAGTGTAGACAGCCG-3’ |
|  |  |  |
| NPC1L1 | Forward | 5’- CTGGTATCACTGGAAGCGAGT-3’ |
|  | Reverse | 5’- CACGCGGGTCACATTGATGA-3’ |
|  |  |  |
| HMGCS1 | Forward | 5’-CATTAGACCGCTGCTATTCTGTC-3’ |
|  | Reverse | 5’-TTCAGCAACATCCGAGCTAGA-3’ |
|  |  |  |
| OCT4 | Forward | 5’- GATGTGGTCCGAGTGTGGTTCTG-3’ |
|  | Reverse | 5’- CCCAGCAGCCTCAAAATCCTCTC-3’ |
|  |  |  |
| cMYC | Forward | 5’- CGTCCTCGGATTCTCTGCTCTC-3’ |
|  | Reverse | 5’- TCCTCATCTTCTTGTTCCTCCTCAG-3’ |
|  |  |  |
| AFP | Forward | 5’- ACCCGAACTTTCCAAGCCATAAC-3’ |
|  | Reverse | 5’- CAGACAATCCAGCACATCTCCTC-3’ |
|  |  |  |
| DUSP9 | Forward | 5’- CGACTGCTCTGATGCGGAATC-3’ |
|  | Reverse | 5’- AGGATCTGGACAGGGAAGGATG-3’ |
|  |  |  |

**Table S17. SiRNA and shRNA sequences used in this study.**

| **Gene name** |  | **Sequences** |
| --- | --- | --- |
|  |  |  |
| SREBF2 siRNA-1 | Sense | 5’- UGCCUACUCUUCUCUUAUGUU-3’ |
|  | Antisense | 5’-CAUAAGAGAAGAGUAGGCAUC-3’ |
|  |  |  |
| SREBF2 siRNA-2 | Sense | 5’-CUGCAAUUUGUCAGUAAUUU-3’ |
|  | Antisense | 5’-UUACUGACAAAUUGCAGCUU-3’ |
|  |  |  |
| integrin a1 siRNA | Sense | 5’- GCCCUUAUAUGCCUAUAGAUU-3’ |
|  | Antisense | 5’-CUAUAGGCAUAUAAGGGCUU-3’ |
|  |  |  |
| IGF2 shRNA |  | 5’GATCCGCCAATATGACACCTGGAAGCACTCGAGTTGTTGATCAATTTGCTTCCGTTTTTT-2’ |
|  |  |  |
| integrin a1 shRNA |  | 5’-GATCCGCCCTTATATGCCTATAGACTCGAGTATTTGACCCAGTCGATGCACTTTTTT-3’ |
